# Supplementary material for: Antioxidant, insecticidal activity and chemical profiling of flower’s extract of Parthenium weed (Parthenium hysterophorus L.)
Source: PLoS One. 2024 Jun 7;19(6):e0296321. doi: 10.1371/journal.pone.0296321 (PMC11161021; doi:10.1371/journal.pone.0296321)
Supplement: Attachment — Submitted filename: Summary of Comments on PONE-D-22-14804_reviewer.pdf [file pone.0296321.s002.pdf]

Introduction

The World Conservation Union stated second utmost substantial threat to biodiversity and habitat are invasive alien species. Moreover, they exert devastating impacts on innate biota and deleteriously affecting ecosystems by destructions of native species. Likewise, such impacts on natural biodiversity could lead towards escalation of climate change pollution and destruction of habitat. However, identifying benefits and harmful impacts of being introduced invasive alien species, might lead towards better understanding and control of biological invasions. In brief, the benefits and drawbacks are contradictory towards the sides of the same coins. Invasive alien plant species are the worldwide risk to biodiversity, functioning of ecosystem and farmed production Adkins, Shabbir (1). Invasive weeds species are undesirable self-grown plants that compete with the cultivated and non-cultivated crops for nutrients, sunlight and water. They also produce a wide range of bioactive particles that are rich sources of various types of medicines. Today, most of the medications depend upon natural plants or in the traditional systems [2].

Parthenium hysterophorus L. is an extremely invasive plant species belonging to family Asteraceae that has invaded almost fifty countries in sub-continent Africa, Asia, and Oceania, infesting forests, feeding lands, and urban landscapes [1, 3]. *P. hysterophorus* is generally recognized by different names such as carrot grass, congress grass, Gajjar botti, white head and white top [4]. It is native to Gulf of Mexico and United States. However, it accidently introduced in Indian subcontinent [5]. This weed has already taken major rank as many indigenous weeds and is spreading at a much faster pace to takeover abandoned land, canal, watercourse banks, roads, and fields to challenge crop productivity.

Summary of Comments on PONE-D-22-14804_reviewer.pdf

Page: 9

Number: 1 Author: A Subject: Sticky Note Date: 22/07/2022 4:20:53 PM
innate of what? do you mean native?

Number: 2 Author: A Subject: Highlight Date: 22/07/2022 4:22:05 PM

Number: 3 Author: A Subject: Sticky Note Date: 22/07/2022 4:22:08 PM

Author: A Subject: Sticky Note Date: 22/07/2022 4:22:26 PM
need to explain how?

Number: 4 Author: A Subject: Cross-Out Date: 22/07/2022 4:23:27 PM

Number: 5 Author: A Subject: Highlight Date: 22/07/2022 4:25:53 PM
No clear, check the grammar

Number: 6 Author: A Subject: Inserted Text Date: 22/07/2022 4:24:46 PM
Again, this need to be explained how?

Number: 7 Author: A Subject: Cross-Out Date: 22/07/2022 4:27:32 PM

Number: 8 Author: A Subject: Highlight Date: 22/07/2022 4:27:14 PM
Is this correct referencing style?

Number: 9 Author: A Subject: Highlight Date: 22/07/2022 4:28:08 PM

Number: 10 Author: A Subject: Sticky Note Date: 22/07/2022 4:29:50 PM
not clear

Number: 11 Author: A Subject: Highlight Date: 22/07/2022 4:29:41 PM

Medicinal plants are the fundamentals of traditional therapeutic systems based on the trusts and observations comprised of hundreds of years [6]. The phytochemical constituents such as phenolic and flavonoid are secondary metabolites involved in therapeutic system, because of these functional phytochemicals [7]. Moreover, *P. hysterophorus* renowned to contain vital biochemical compounds such as alkaloids, tannins, and saponins, which are important from the medicinal viewpoint [8]. It also exhibited allelopathic effects and important phytochemicals such as parthe[illegible]on and coronopilin which are auto-toxic to its own seed germination and seedling growth [9].

Furthermore, serious consequences of health on human beings and livestock have been reported [10]. Agriculturists are associated with *P. hysterophorus* because; the pollen and dust produced by this weed provoke allergic contact dermatitis in human beings. Major chemical compounds, parthenin's contained by *P. hysterophorus* exhibited phytotoxic properties against a variety of plants, including weeds and various crops such as *Amaranthus viridis, Ageratum conyzoides, Avena fatua, Chenopodium murale, Cassia tora, Phaseolus aureus* [11, 12]. Moreover, parthenin is the main biochemical compounds showed insecticidal activity against termites, cockroaches and migratory grasshoppers, *Melanoplus sanguinipes* [4]. Similarly, Patel, Chitra [13] reported pesticidal activity of the ethanol extract of *P. hysterophorus* which showed substantial lethal effect against stored grain pest like *Tribolium sp.* and *Oryzaephilus sp.* Essential oils from the natural plants also have antioxidant effects. In recent years several reports have been breakout, the essential oil from Rosemary [14], *Mentha longifolia* var. *Calliantha [15], Curcuma caesia* [16] *Ammodaucus leucotrichus* [17] and *Piper nigrum* [18].

Pharmacologically, it is involved in treating numerous diseases like allergies, anemia, sores, skin diseases, facial neuralgia, fever, vermifuge, blood purifier, and

Page: 10

Number: 1 Author: A Subject: Cross-Out Date: 5/08/2022 10:36:41 AM

Number: 2 Author: A Subject: Cross-Out Date: 5/08/2022 10:36:31 AM

Number: 3 Author: A Subject: Sticky Note Date: 5/08/2022 10:38:13 AM
Do you mean parthenin?

Number: 4 Author: A Subject: Highlight Date: 5/08/2022 10:37:50 AM

Number: 5 Author: A Subject: Sticky Note Date: 5/08/2022 10:39:50 AM
move 'health' before 'consequences'

Number: 6 Author: A Subject: Cross-Out Date: 5/08/2022 10:39:02 AM

Number: 7 Author: A Subject: Highlight Date: 5/08/2022 10:39:13 AM

Number: 8 Author: A Subject: Highlight Date: 5/08/2022 10:41:10 AM
Do you mean "come in contact'

Number: 9 Author: A Subject: Sticky Note Date: 5/08/2022 10:42:41 AM
add author citations with the sci. names of all plants when mentioned first time

Number: 10 Author: A Subject: Highlight Date: 5/08/2022 10:41:33 AM

Number: 11 Author: A Subject: Cross-Out Date: 5/08/2022 10:43:27 AM

Number: 12 Author: A Subject: Highlight Date: 5/08/2022 10:43:49 AM

Number: 13 Author: A Subject: Highlight Date: 5/08/2022 10:44:26 AM

All calculated data was analysed by one way analysis of variance (ANOVA), mean difference between treatments was calculated for significance test by Duncan Multiple Range Test “DMRT ” at *P*> 0.05) with IBM-SPSS statistics 25.0 version software. Probit analysis was performed using EPA Probit analysis program version 1.5.

Results

The current study carried out on the flower’s extract of *Parthenium hysterophorus* revealed the existence of medicinally and agro-chemically active compounds. The phytochemical constituents of *P. hysterophorus* were qualitatively analyzed for antioxidant and insecticidal activities.

Extract Yield (%)

Yield of extract by the solvent extraction technique from *P. hysterophorus* flowers was calculated and presented in (Figure 1). The maximum extract was obtained by ethanol (4.34%) whereas; minimum extract quantity (2.39%) was afforded by dichloromethane. The extract obtained via ethanol and ethyl acetate was bluish green and greenish in color and exhibited consistency, respectively. The same oil was brownish in color and non-consistent via dichloromethane and distilled water, respectively, (Table 1).

Analysis for Phenol and Flavonoid and Antioxidant Activity

The total contents for phenol and flavonoid as well as DPPH scavenging activity afforded by *P. hysterophorus* flower’s extract via different solvents are presented in (Table 2). Results confirmed that the ~~maximum~~ total phenol were reported from distilled water 103.30 GAE/g followed by Ethyl acetate and Ethanol’ extract, 47.79

Page: 16

Number: 1 Author: A Subject: Sticky Note Date: 5/08/2022 10:52:51 AM
highest?

Number: 2 Author: A Subject: Cross-Out Date: 5/08/2022 10:52:35 AM

and 27.55 GAE/g, respectively. On the other hand maximum flavonoid was reported from Ethyl acetate extract 138.67 QE/g followed by Ethanol 14.23 QE/g respectively.

Inhibition (%) was determined by 1,1-Diphenyl-1-picrylhydrazyl (DPPH) displayed color absorption at 517nm on using spectrophotometer. When DPPH trap free radicals, the color of the solution changed to light yellow and, subsequently, resulted in decrease of the absorbance. Maximum DPPH radical scavenging activity was afforded by Dichloromethane extract 95.03% followed by Ethanol and Ethyl acetate 88.53% and 67.21%, respectively.

Correlation of Antioxidant Activity versus Phenols, Flavonoids and extract yield

In our study, the Pearson's correlation regarding antioxidant activity of *P. hysterophorus* showed positive relationship (Table 3). Results showed highly significant ($P<0.01$) negative effect of phenol with DPPH as well as DPPH showed strongly highly significant negatively correlation and similar results were found reciprocally in the studied parameters. However, yield extracted by different solvents did not affect the Antioxidant Activity versus phenols, flavonoids quantitatively.

Assessment of Insecticidal Activity

The mortality data of cabbage aphid *B. brassicae* at specific period of time using *P. hysterophorus* flower's extract is presented in (Figure 2). It was perceived that the percent mortality was directly correlated to the concentration and the time. The results demonstrated that the maximum mortality was recorded at 1000 µgmL^{-1} at 96 h and 72 h with 63.33% and 46.67%, respectively. Same trend of mortality was also observed at 800 µgmL^{-1} with 56.67% and 36.67%, respectively. Imidacloprid 25% WP being a positive control produced the highest rates of mortality after 96 h of

Page: 17

Number: 1 Author: A Subject: Highlight Date: 5/08/2022 10:53:02 AM

Appraisal of antioxidant and polyphenols activity of medicinal products has become vital device to recognize the medicinal possessions of natural plants. An antioxidant is a molecule which exhibited the ability to inhibit the oxidation of other chemical elements. Kumar, Mishra (44] reported that alkaloids, flavonoids, terpenoids, and cardiac glycosides from the extract of *Parthenium hysterophorus*. However, the phenolic contents in flower extracts were ranging from 86.69-320.17 mg propyl gallate equivalent (PGE)/g. Study established that bio-chemicals possessed by *P. hysterophorus* extracts bears significant antioxidant activity consistent to our current findings. Similarly, 50% radical scavenging potential of *P. hysterophorus* extract at 250 µgmL^{-1} using DPPH assay as well as 30% inhibition at 100 µgmL^{-1} via hydrogen per oxide assays was reported from *P. hysterophorus* leaves extract [45]. Antioxidant activities of hydro-ethanolic extracts of *P. hysterophorus* leaves was perhaps because of the existence of polyphenols contents [6, 46].

Likewise, Sinha and Paul [47] found that extract from *P. hysterophorus* possess valuable antioxidant activity with 54.5% scavenging property and 60.2 IC_{50} via DPPH assay while, 93.2% and 33.6 IC_{50} via ABTS assay which are consistent with our findings on antioxidant activity. Bashir (48] evaluated leaf extract of *P. hysterophorus* for the presence of total phenolic and flavonoid contents as 105.44mg GAE/g and 41.50mg RE/g respectively exhibited by 60% ethanolic extract with IC_{50} value of 87.55 µgmL^{-1} and 98.22 µgmL^{-1} for DPPH radical scavenging activity respectively supported our investigations on antioxidant as well as phenol and flavonoid contents from *P. hysterophorus*. Phytochemical profile of *P. hysterophorus* root, stem, leaf and phyllary identified terpenes, fatty acids, hydrocarbons and phytosterols however; root extracts showed highest antioxidant potential [49]. The results obtained on antioxidant activity of *P. hysterophorus* are in agreement with Sinha and Paul (47] who reported

Number: 1 Author: A Subject: Highlight Date: 5/08/2022 10:57:22 AM

insecticidal as well as nematicidal activity against the *Callosobruchus maculatus* adults and root knot nematode *Meloidogyne incognita*. Similarly, the pyrazoline was found highly effective being an insecticide, with LC_{50} value 32 mgl^{-1} with exposure of 72 h, and found extremely effective as nematicidal activity with LC_{50} values being 512 mgl^{-1} perceived for parthenin at 72 h exposure [32].

Parthenium Leave's extract used against mustard aphid, *Lipaphis erysimi* under field conditions exhibited incredible decrease of 29% of the infestation which may be because of the effect of phenolic acids [54]. Ahmad, Bagheri (49) reported the efficacy of *P. hysterophorus* extract with 10 and 20% concentration found 26.70% and 27% mortality at 21 day after treatment. Consequently, Wasu Yogesh H (55] in their study reported the activity of leaves and stem extract of *P. hysterophorus* against different larval instars of *Aedes aegypti*. Stored stock and freshly prepared solution caused 80% and 100% mortality, respectively with concentration prepared in 200g/500 ml. Another study reported that crude aqueous extracts of whole plant of *P. hysterophorus* caused a significant mortality with 73.33, 86.67 and 83.33% of *B. brassicae* as well as moderate mortality being 26.67, 46.67 and 63.33% of *Myzus persicae* at 10% concentration at 24, 48 and 72h exposure, respectively (Yadav and Patel 2017). Consequently, [55] in a study, predicted that crude extracts from fresh leaves of *P. hysterophorus* showed significant effects against *Spodoptera littura* and *Spodoptera littorallis* with LC_{50} values as 7.96% and 8.14%, respectively.

Gas Chromatographic- Mass Spectrometric (GC-MS) analyses offered the existence of various phytochemicals in different parts of the plants. In a systematic chemical study Wang, Gan (56], reported that solvent fraction of *P. hysterophorus*, contained fourteen chemical compounds were plant phytosterols, stigmasterol, daucosterol, dtigmasterol-3-O-glucopyranoside, and Stigmasteryl-3β-arachidate. The

Page: 21

Number: 1 Author: A Subject: Sticky Note Date: 5/08/2022 10:58:50 AM
leaves'

Number: 2 Author: A Subject: Sticky Note Date: 5/08/2022 10:59:38 AM
Either use Parthenium or Parthenium hysterophorus, throughout

Number: 3 Author: A Subject: Cross-Out Date: 5/08/2022 10:58:39 AM

Number: 4 Author: A Subject: Highlight Date: 5/08/2022 10:58:54 AM

Nextgenediting Template PLOS_One_Word_Template.docx
www.nextgenediting.com [double click header to delete]

Table 4. **Toxicity of *P. hysterophorus* extracts against *B. brassicae* at 12, 24, 48, 72 and 96 hours exposure**

Time	LC_{50} µgmL^{-1}	[illegible]5% C.L		Slope± S.E	χ2
		Lower	Upper		
12	3598	1421	35512	1.09±0.38	0.25
24	2569	1321	27758	1.51±0.46	0.99
48	2345	1153	25517	1.31±0.53	0.85
72	1446	852	21721	1.56±0.63	0.22
96	750	468	1276	1.89±0.58	0.04

Note: LC_{50} (Lethal concentration); S.E. (standard error); χ2 (chi-square); F.L. (Fiducial limit)

Page: 36

Number: 1 Author: A Subject: Sticky Note Date: 5/08/2022 11:05:58 AM

C.L stands for?

Number: 2 Author: A Subject: Highlight Date: 5/08/2022 11:05:42 AM

Fig 2. Insecticidal activity of extract of *Parthenium hysterophorus* flowers. Data in the bars is described as mean values ± standard error with various superscripts is significantly different according to DMRT" P> 0.05). Whereas; A (100 µgmL-1); B (200 µgmL-1); C (400 µgmL-1); D (800 µgmL-1); E (1000 µgmL-1); F (-iv Control); G (+iv Control).

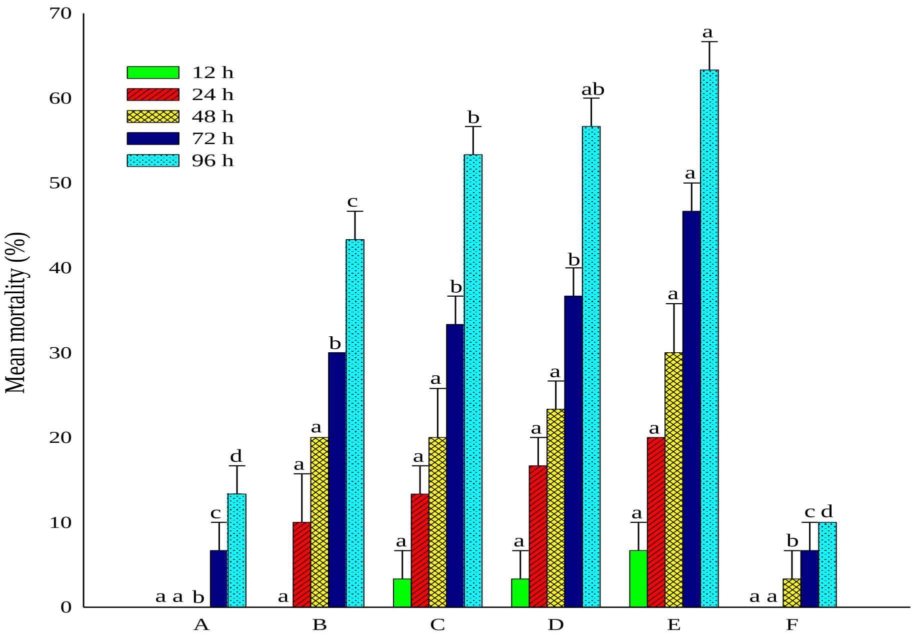

Page: 39

Number: 1 Author: A Subject: Sticky Note Date: 5/08/2022 11:09:04 AM

G = +ve control missing in Figure

Number: 2 Author: A Subject: Highlight Date: 5/08/2022 11:09:11 AM
