# Supplementary material for: Antioxidant, insecticidal activity and chemical profiling of flower’s extract of Parthenium weed (Parthenium hysterophorus L.)
Source: PLoS One. 2024 Jun 7;19(6):e0296321. doi: 10.1371/journal.pone.0296321 (PMC11161021; doi:10.1371/journal.pone.0296321)
Supplement: Attachment — Submitted filename: PONE-D-22-14804_R3_reviewer.pdf [file pone.0296321.s007.pdf]

PLOS ONE

Antioxidant, Insecticidal Activity and Chemical Profiling of Flower’s Extract of Carrot Grass (Parthenium hysterophorus L.)

--Manuscript Draft--

**Manuscript Number:**	PONE-D-22-14804R3
**Article Type:**	Research Article
**Full Title:**	Antioxidant, Insecticidal Activity and Chemical Profiling of Flower’s Extract of Carrot Grass (Parthenium hysterophorus L.)
**Short Title:**	Biological activities and chemical profiling of Parthenium hysterophorus L.
**Corresponding Author:**	Zumin Gu, Ph.D.<br>Shenyang Agricultural University<br>Shenyang, Liaoning CHINA
**Keywords:**	Parthenium hysterophorus; Phytochemicals; antioxidant activity; GCMS; DPPH
**Abstract:**	Parthenium hysterophorus L . an invasive alien species is considered a noxious weed and confers numerous benefits in medical and agrochemical industries. The present study evaluated the flower extract's antioxidant and insecticidal activity and chemical profiling to appraise the existence of phytoconstituents responsible for biological activities. Assay was performed by 1,1-diphenyl-2-picrylhydrazyl (DPPH) for antioxidant activity, whereas, chemical configuration was evaluated by Gas Chromatography Mass Spectrometry (GCMS) analysis. Our findings report that the extract of P. hysterophorus possesses potential radical scavenging activity (95.03%) when extracted via dichloromethane (DCM). Additionally, phytochemical analysis revealed a considerable amount of phenols and flavonoid contents when extracted via distilled water and ethyl acetate; (103.30 GAEg $^{-1}$ and 138.67 QE-g $^{-1}$) respectively. Maximum mortality 63.33% and 46.67% was observed at 1000 μgmL $^{-1}$ and 800 μgmL $^{-1}$ after 96 h exposure followed by 46.67% and 36.67% at 72 h at the same concentration. On the other hand, toxicity results displayed that P. hysterophorus extracts showed LC_{50} values of 1446 and 750 μgmL $^{-1}$ at 72 and 96 h exposure, respectively. However, Imidacloprid being a positive control afforded maximum mortality at 96 h and 72 h exposure 97.67% and 91.82%, respectively. Moreover, antioxidant activity of P. hysterophorus extracts displayed strong correlation versus phenols, flavonoids and extract yield. GCMS analysis revealed the existence of 13 chemical compounds corresponding to 99.99% of whole extract. Moreover, maximum extract was afforded by Ethanol followed by distilled water, ethyl acetate and dichloromethane as 4.34, 3.22, 3.17 and 2.39%, respectively. P. hysterophorus flower’s extract therefore showed good antioxidant and aphicidal activity which also exhibited valuable chemical compounds accountable for biological activity, making them suitable candidates for an alternative to synthetic agents. However, our results provide novel and fundamental basis for further exploration on purification of chemical compounds for their biological activities.
**Order of Authors:**	Maqsood Ahmed, PhD.
	Ansar Javeed
	Sami Ullah, M.Phil.
	Talfoor ul Hassan, M.Phil.
	Aatika Sikandar, PhD.
	Taswar Ahsan, PhD.
	Faqir Ahmad, M.Phil.
	Mingshan Ji, Post Doc.
	Xuejing Bai, PhD.
	Ying Li, PhD.
	Zumin Gu, Post Doc.

Powered by Editorial Manager® and ProduXion Manager® from Aries Systems Corporation

Response to Reviewers:	A file is uploaded ad rebuttal letter as well as Response to reviewers
Additional Information:	
**Question**	**Response**
**Financial Disclosure**<br><br>Enter a financial disclosure statement that describes the sources of funding for the work included in this submission. Review the submission guidelines for detailed requirements. View published research articles from *PLOS ONE* for specific examples.<br><br>This statement is required for submission and **will appear in the published article** if the submission is accepted. Please make sure it is accurate.<br><br>**Unfunded studies**<br>Enter: *The author(s) received no specific funding for this work.*<br><br>**Funded studies**<br>Enter a statement with the following details:<br>• Initials of the authors who received each award<br>• Grant numbers awarded to each author<br>• The full name of each funder<br>• URL of each funder website<br>• Did the sponsors or funders play any role in the study design, data collection and analysis, decision to publish, or preparation of the manuscript?<br>• **NO** - Include this sentence at the end of your statement: *The funders had no role in study design, data collection and analysis, decision to publish, or preparation of the manuscript.*<br>• **YES** - Specify the role(s) played.<br><br>* typeset	This research was supported by the Supported by National Key Research & Development Program of China (Grant No. (0201300). The funders had no role in study design, data collection and analysis, decision to publish, or preparation of the manuscript.
**Competing Interests**<br><br>Use the instructions below to enter a competing interest statement for this submission. On behalf of all authors, disclose any competing interests that could be perceived to bias this work—acknowledging all financial support and any other relevant financial or non-financial competing interests.	The authors declare no conflict of interest

This statement is required for submission and **will appear in the published article** if the submission is accepted. Please make sure it is accurate and that any funding sources listed in your Funding Information later in the submission form are also declared in your Financial Disclosure statement.<br><br>View published research articles from *PLOS ONE* for specific examples.<br><br>**NO authors have competing interests**<br><br>Enter: *The authors have declared that no competing interests exist.*<br><br>**Authors with competing interests**<br><br>Enter competing interest details beginning with this statement:<br><br>*I have read the journal's policy and the authors of this manuscript have the following competing interests: [insert competing interests here]*<br><br>* typeset	
**Ethics Statement**<br><br>Enter an ethics statement for this submission. This statement is required if the study involved:<br><br>• Human participants<br>• Human specimens or tissue<br>• Vertebrate animals or cephalopods<br>• Vertebrate embryos or tissues<br>• Field research<br><br>Write "N/A" if the submission does not require an ethics statement.<br><br>General guidance is provided below. Consult the submission guidelines for detailed instructions. **Make sure that all**	N/A

**information entered here is included in the Methods section of the manuscript.**<br><br>**Format for specific study types**<br><br>**Human Subject Research (involving human participants and/or tissue)**<br>• Give the name of the institutional review board or ethics committee that approved the study<br>• Include the approval number and/or a statement indicating approval of this research<br>• Indicate the form of consent obtained (written/oral) or the reason that consent was not obtained (e.g. the data were analyzed anonymously)<br><br>**Animal Research (involving vertebrate animals, embryos or tissues)**<br>• Provide the name of the Institutional Animal Care and Use Committee (IACUC) or other relevant ethics board that reviewed the study protocol, and indicate whether they approved this research or granted a formal waiver of ethical approval<br>• Include an approval number if one was obtained<br>• If the study involved *non-human primates*, add *additional details* about animal welfare and steps taken to ameliorate suffering<br>• If anesthesia, euthanasia, or any kind of animal sacrifice is part of the study, include briefly which substances and/or methods were applied<br><br>**Field Research**<br><br>Include the following details if this study involves the collection of plant, animal, or other materials from a natural setting:<br>• Field permit number<br>• Name of the institution or relevant body that granted permission	
**Data Availability**<br><br>Authors are required to make all data underlying the findings described fully available, without restriction, and from the time of publication. PLOS allows rare exceptions to address legal and ethical	Yes - all data are fully available without restriction

concerns. See the PLOS Data Policy and FAQ for detailed information.<br><br>A Data Availability Statement describing where the data can be found is required at submission. Your answers to this question constitute the Data Availability Statement and **will be published in the article**, if accepted.<br><br>**Important:** Stating 'data available on request from the author' is not sufficient. If your data are only available upon request, select 'No' for the first question and explain your exceptional situation in the text box.<br><br>Do the authors confirm that all data underlying the findings described in their manuscript are fully available without restriction?	
**Describe where the data may be found in full sentences. If you are copying our sample text, replace any instances of XXX with the appropriate details.**<br><br>• If the data are **held or will be held in a public repository**, include URLs, accession numbers or DOIs. If this information will only be available after acceptance, indicate this by ticking the box below. For example: *All XXX files are available from the XXX database (accession number(s) XXX, XXX.*).<br>• If the data are all contained **within the manuscript and/or Supporting Information files**, enter the following: *All relevant data are within the manuscript and its Supporting Information files.*<br>• If neither of these applies but you are able to provide **details of access elsewhere**, with or without limitations, please do so. For example:<br><br>*Data cannot be shared publicly because of [XXX]. Data are available from the XXX Institutional Data Access / Ethics Committee (contact via XXX) for researchers who meet the criteria for access to confidential data.*	All relevant data are within the manuscript .

The data underlying the results presented in the study are available from (include the name of the third party and contact information or URL).<br>• This text is appropriate if the data are owned by a third party and authors do not have permission to share the data.<br><br>* typeset	
Additional data availability information:	Tick here if the URLs/accession numbers/DOIs will be available only after acceptance of the manuscript for publication so that we can ensure their inclusion before publication.

Powered by Editorial Manager® and ProduXion Manager® from Aries Systems Corporation

Manuscript

Click here to access/download;Manuscript;PLOS_One_Maqsood Final

Original Manuscript

Antioxidant, Insecticidal Activity and Chemical Profiling of Flower's Extract of Carrot Grass (*Parthenium hysterophorus* L.)

Maqsood Ahmed[1,2], Ansar Javeed[3], Samiullah[2], Talfoor-ul-Hassan[4], Aatika Sikandar[1], Taswar Ahsan[5], Faqir Ahmed[6], Ji Mingshan[1], Xuejing Bai[7], Ying Li[8] and Zumin Gu[1,*]

[1]College of Plant Protection, Shenyang Agricultural University, Shenyang, 110866, P.R. China. aatika_sikander@yahoo.com (A.S.); jimingshan@syau.edu.cn (M.J.)
[2]Department of Agriculture (Plant Protection), Pest Warning and Quality Control of Pesticides, Gujrat, 50700, Pakistan. maqsoodahmed200@hotmail.com
[3]Henan University, Jinming Campus, Kaifeng City Henan China. ansarjaveed@henu.edu.cn (A.J)
[4]Department of Agriculture (Plant Protection), Pest Warning and Quality Control of Pesticides, Gujranwala, 52250, Pakistan. ddappgujranwala@gmail.com (T.u.H.)
[5]Institute of Plant Protection Liaoning Academy of Agricultural Sciences, Shenyang, 110866, P.R. China. taswarahsan@163.com (T.H)
[6]Directorate General of Agriculture, Pest Warning and Quality Control of Pesticides, Lahore, 54800, Pakistan. faqirahmad63@yahoo.com (F.A)
[7]Shenyang Academy of Landscape- Gardening Shenyang 110016, P.R. China. baixuejingnv@163.com (X.B.)
[8] Fushun Modern Agriculture and Poverty Alleviation Development Promotion Centre, Fushun 113006 P.R. China. liying_1973@163.com (Y.L.)

Corresponding author: *guzumin1212@163.com *(G.Z); Tel: +86-24- 8848 - 7148; Fax: +86-24-8834-2315*

Abstract

Parthenium hysterophorus L. an invasive alien species is considered a noxious weed and confers numerous benefits in medical and agrochemical industries. The present study evaluated the flower extract's antioxidant and insecticidal activity and chemical profiling to appraise the existence of phytoconstituents responsible for biological activities. Assay was performed by 1,1-diphenyl-2-picrylhydrazyl (DPPH) for antioxidant activity, whereas, chemical configuration was evaluated by Gas Chromatography Mass Spectrometry (GCMS) analysis. Our findings report that the extract of *P. hysterophorus* possesses potential radical scavenging activity (95.03%) when extracted via dichloromethane (DCM). Additionally, phytochemical analysis revealed a considerable amount of phenols and flavonoid contents when extracted via distilled water and ethyl acetate; (103.30 $GAEg^{-1}$ and 138.67 $QE\text{-}g^{-1}$) respectively. Maximum mortality 63.33% and 46.67% was observed at 1000 μgmL^{-1} and 800 μgmL^{-1} after 96 h exposure followed by 46.67% and 36.67% at 72 h at the same concentration. On the other hand, toxicity results displayed that *P. hysterophorus* extracts showed LC_{50} values of 1446 and 750 μgmL^{-1} at 72 and 96 h exposure, respectively. However, Imidacloprid being a positive control afforded maximum mortality at 96 h and 72 h exposure 97.67% and 91.82%, respectively. Moreover, antioxidant activity of *P. hysterophorus* extracts displayed strong correlation versus phenols, flavonoids and extract yield. GCMS analysis revealed the existence of 13 chemical compounds corresponding to 99.99% of whole extract. Moreover, maximum extract was afforded by Ethanol followed by distilled water, ethyl acetate and dichloromethane as 4.34, 3.22, 3.17 and 2.39%, respectively. *P. hysterophorus* flower's extract therefore showed good antioxidant and aphicidal activity which also exhibited valuable chemical compounds accountable for biological activity, making them suitable candidates for an alternative to synthetic agents. However, our results provide novel and fundamental basis for further exploration on purification of chemical compounds for their biological activities.

Keywords: *Parthenium hysterophorus*; phytochemicals; Antioxidant activity; GCMS; DPPH

Introduction

The invasive alien species exert devastating impacts on inherent biota and deleteriously affecting ecosystems by destructions of native species. Likewise, such distressing impacts on natural biodiversity could lead towards escalation of climate change, environmental pollution, and ecological destruction of natural habitat. However, identifying the beneficial and harmful impacts of *Parthenium hysterophorus* introduced as an invasive alien species, might lead towards control of biological invasions. Invasive alien plant species are the worldwide risk to biodiversity, functioning of ecosystem and farm production [1]. Invasive weeds species are undesirable self-grown plants that compete with the cultivated and non-cultivated crops for nutrients, sunlight and water. They also produce a wide range of bioactive particles that are rich sources of various types of medicines. Currently, most of the medications in the pharmaceutical industry depend upon natural plants and or in the herbal traditional systems [1].

Parthenium hysterophorus L. is an extremely invasive plant species belonging to family Asteraceae that has invaded almost fifty countries in sub-continent Africa, Asia, and Oceania, infesting forests, feeding lands, and urban landscapes [2, 3]. It is a devastating and hazardous weed of various economically important crops [4]. Moreover, it is amongst the top ten worst weeds in the world and is included in the world catalogue of destructive species [5, 6]. In Europe *P. hysterophorus* is considered as an ephemeral species. It is generally recognized by different names such as carrot grass, congress grass, Gajjar botti, white head and white top [7]. It is native to Gulf of Mexico and United States. However, it accidently introduced in Indian subcontinent [8]. This weed has been considered as a major weed of high rank

spreading at a much faster pace to takeover abandoned land, canal, watercourse banks, roads, and fields to challenge crop productivity [9, 10].

Medicinal plants are the fundamentals of traditional therapeutic systems based on the trusts and observations comprised of hundreds of years [11]. The phytochemical constituents such as phenolic and flavonoid are secondary metabolites involved in therapeutic system, because of the existence of these functional phytochemicals mainly parthenin [12]. Moreover, *P. hysterophorus* renowned to contain vital biochemical compounds such as alkaloids, tannins, and saponins, which are important from the medicinal viewpoint [13]. It also exhibited allelopathic effects and important phytochemicals such as parthenon and coronopilin which are auto-toxic to its own seed germination and seedling growth [14]. Furthermore, serious health consequences on human beings and livestock have been reported [15]. The agriculturists who are come in contact with *P. hysterophorus*; the pollen and dust produced by this weed provoke allergic contact dermatitis in human beings. Major chemical compounds, parthenin's contained by *P. hysterophorus* exhibited phytotoxic properties against a variety of plants, including weeds and various crops such as Slender amaranth (*Amaranthus viridis*), Billygoat weed (*Ageratum conyzoides*), common wild oat. (*Avena fatua*), nettleleaf goosefoot (*Chenopodium murale*), Foetid cassia (*Cassia tora*), mung bean *Phaseolus aureus* [16, 17]. Moreover, parthenin's the main biochemical compounds showed insecticidal activity against termites, cockroaches and migratory grasshoppers, *Melanoplus sanguinipes* [7]. Similarly, Patel, Chitra [18] reported pesticidal activity of the ethanol extract of *P. hysterophorus* which showed substantial lethal effect against stored grain pest like *Tribolium* sp. and *Oryzaephilus* sp. Essential oils from the natural plants also have antioxidant effects. In recent years several reports have been breakout, the essential oil from Rosemary [19], wild mint

(*Mentha longifolia* var. *Calliantha*) [20], black turmeric (*Curcuma caesia*) [21] *Ammodaucus leucotrichus* [22] and black pepper (*Piper nigrum*) [23].

Pharmacologically, it is involved in treating numerous diseases like allergies, anemia, sores, skin diseases, facial neuralgia, fever, vermifuge, blood purifier, and insecticide [7, 24]. This plant species is enriched with vital phytoconstituents however; sesquiterpene lactones are the most copious phytochemicals exhibited by this plant which are accountable for injurious effects such as strong allelopathic [25], toxic to livestock [26], It also possess many advantageous effects like antibacterial [27, 28], acaricidal [29], antioxidant [30, 31], cytotoxic [32, 33], larvicidal potential against *Aedes aegypti* L. [34, 35], skeletal muscle relaxant [36], pesticidal agent [37], producing bioethanol [38], as reducing agent in the formation of silver nanoparticles [39] and in production of zinc nanoparticles [40]. However, various factors affect the biological profile of the plants and from some recent studies it has been reported that nanoparticle influenced the physiological and biological properties of the plants. It has been reported that metal-based nanoparticle showed a decrease in protein and amino acid and micronutrient contents in wheat crop grown under such conditions [41]. Moreover, nitrogen doped titanium dioxide nanoparticle enhanced antioxidant activity via reducing power assay and percent inhibition of DPPH against *Streptomyces* [42]. Likewise, synthetically and biologically prepared nanoparticle significantly exerted effects on plant growth and secondary metabolites which ultimately triggered the biological activity of the plants [43].

As some earlier researches have been conducted on this plant species but, detailed studies on chemical and quantitative phytochemical analysis and assessment of antioxidant activities from different solvents extract as well as aphicidal activity against cabbage aphid *B. brassicae* from flower’s extracts has not been explored

before. Hence, keeping in view the diverse biological properties of *P. hysterophorus*, current study was designed to determine the antioxidant and insecticidal activity of flower's extract against *B. brassicae* with appraisal of quantitative phytochemical analysis as well as assessment of chemical profiling of extract.

Materials and Methods

Collection and Samples Preparation

The whole plant samples were collected from District Sialkot Punjab Pakistan during the month of June-July 2019 and were identified as *Parthenium hysterophorus* L., from Ayub Agricultural Research Institute (AARI) Faisalabad Pakistan by Dr. Dilbar Hussain. Flowers were manually separated from the plants and left over for 20 days to dry under shade. These dried flowers were powdered by electric grinder and then extracted with different solvents (ethanol, ethyl acetate, dichloromethane and distilled water) by cold extraction method for seventy two hours at fixed temperature 28°C and at 100 rpm in an incubator shaker (ZWY-1102C). Extracted contents were filtered and concentrated to lessen the volume on rotary evaporator model R-210 BUCHI Labortechnik AG, CH-9230 Flawil 1/Switzerland). Obtained extract's yield was measured using following Equation (Eq. 1).

$$\text{Yield (\%)} = \frac{\text{Weight of the extract}}{\text{Weight of the dried sample}} \times 100 \qquad \text{(Eq. 1)}$$

Quantitative Analysis for Total Phenolic and Flavonoids Content

To assess the phytochemicals quantitatively, 1 mg of extract from each solvent was dissolved in 1 mL Methanol separately. Consequent mixture was vortexed and separated for analysis. For the assessment of total content for phenols Folin–Ciocalteu reagent test was used. In brief, 1 mL solution (1 $mgmL^{-1}$) was added to 2.5 mL of (10%) Folin–Ciocalteu with supplementary addition of 2 mL solution of (2%) sodium carbonate (Na_2CO_3). The consequential blend was incubated for fifteen min in the

dark to measure absorbance at 765 nm in 96-well ELISA plate using (SpectraMax 190, Meigu molecular International Co. Ltd., Shanghai China). To build a standard curve, Gallic acid was used (1 $mgmL^{-1}$) at different concentrations such as 1, 0.50, 0.25, 0.10, 0.05, 0.02, 0.01, 0 mg mL^{-1} to describe the obtained results as Gallic acid equivalent (GAE) mg g^{-1} of total extract. Each treatment was replicated ten times to analyze the data.

To examine the total flavonoids content, aluminium chloride colourimetric method was adopted. Briefly, 1 mL solution (1$mgmL^{-1}$) was poured into 3 mL methanol, with addition of 0.2 mL of 1 M Potassium acetate (CH_3COOK), 0.2 mL of (10%) aluminium chloride ($AlCl_3$) and at the end, distilled water 5.6 mL was added to the consequent mixture. The incubation of solution was performed for half an hour in the darkness and absorbance at 420 nm was measured. A standard curve was built using quercetin at different concentrations such as 1, 0.50, 0.25, 0.10, 0.05, 0.02, 0.01, 0 $mgmL^{-1}$ to represent the results as quercetin equivalent (QE) mg g^{-1} of total extract. The procedure was replicated 10 times for each treatment.

Assessment of Free Radical Scavenging Activity

Antioxidant activity of extracts obtained by different solvents from *Parthenium hysterophorus* flowers was accomplished in 1% solution of tween 20 by using 1,1-diphenyl-2-picrylhydrazyl (DPPH) ($C_{18}H_{13}N_5O_6$) a free radical. In brief, to assess the free radical scavenging activity, all dried solvent extracts were mixed together in equal proportion (5 mg each) and were dissolved in HPLC grade methanol and filtered. The filtrate was formerly used to assess the antioxidant activity. Similarly, DPPH solution was prepared in same grade Methanol @ 0.001g 25 mL^{-1}. Then, into 3.5 mL freshly prepared DPPH solution and 0.25 mL extract prepared in Methanol was added. The consequent mixture was shacked for complete mixing and then the

resulting mixture was incubated in darkness at 28°C for 30 min [44]. After incubation of resulting mixture for stipulated period of time, the absorbance was assessed at 517nm by using (SpectraMax 190, Meigu molecular International Co. Ltd., Shanghai China). The inhibition percentage of primed DPPH solution was predicted on reduction of absorbance through following Equation (Eq. 2). Each treatment was replicated ten times to analyze the data.

$$\text{Inhibition (\%)} = \frac{A_{\text{blank}} - A_{\text{sample}}}{A_{\text{blank}}} \times 100 \qquad \text{(Eq. 2)}$$

Where: A_{blank} = (control absorbance); A_{Sample} = (samples absorbance).

Correlation of Antioxidant Activity versus Phenols, Flavonoids and extract yield

Correlation of antioxidant activity *P. hysterophorus* versus Phenols, Flavonoids and extract yield was carried out using SPSS statistics 25.0 version with significant values at ($P>0.05$) level.

Assessment of Insecticidal Activity

Residual toxicity method was adopted for the assessment of insecticidal activity of extract of *P. hysterophorus* flowers. Samples for bioassay study were prepared to obtain mother mixture by mixing 0.1g of each solvent's extract and dissolved in Acetone, because it is aprotic solvent having intermediate polarity comparable to polar parotic solvents and it also evaporates immediately from the extract when exposed to air. The resulting mixture was placed in the fume hood for six hours for complete drying of Acetone. Serial concentrations *viz*. 100, 200, 400, 800 and 1000 µg mL^{-1} was prepared in Tween-20 (1%) solution from mother mixture. Briefly, fresh cabbage leaf discs 5cm in diameter were cut off and dipped for 10 s in the respective concentration and then dried for 5 min at room temperature. Formerly, 10 adult wingless aphids were carefully released onto the leaf disc using a fine-haired brush contained in the Petri dish and incubated for 96 h at 65% R.H., 25°C and with a 16:8

(light: dark) photoperiod. Imidacloprid 25% WP was used as the positive control at a rate of 0.0025 mL mL^{-1} of water, and control (CK) with a 1% Tween 20 solution. Positive controls and CKs were placed in a greenhouse for 96 h. Mortality data was collected at 12, 24, 48, 72 and 96 h using binocular microscope and the response of aphids was observed by needle probing, and the aphids who offered no response on stimulation were considered dead. Each treatment was replicated five times.

Chemical Analysis

Samples for analysis were prepared by mixing 0.1 g of each solvent extract into 2 mL of HPLC grade methanol to obtain mother solution. Consequent mixture was vortexed for 1 min following the addition of graphitized carbon black "GCB" (0.005 g). Mixture was centrifuged for 10 min at 10000 rpm at 25°C and colorless material was separated and collected in (1.5 mL) centrifuges tubes. Work for GC-MS analysis was accomplished using an Agilent Model 6890-5973N accompanied with gas chromatograph established on HP1 capillary column TG-5MS polydimethylsiloxane (length 30m × diameter 250 μm × film thickness 0.25 μm) interfaced with mass selective detector Hewlett Packard (5973N). The established parameters were; preliminary temperature was adjusted at 70°C for 0 min however, final temperature was extended to 200 °C for 10°C min^{-1} whereas, inlet temperature was adjusted at 250°C with split ratio 10:1. MS quadruple temperatures were fixed at 150°C while thermal aux temperatures were 285°C. The Scan range for MS was 35–520 units however; as a carrier, helium gas was used bearing stream frequency of 1.0mLmin^{-1}. The existing compounds in the extract were identified by comparison of their retention time and the mass spectra of the NIST 08 and Wiley 7 of the data base and confirmed by comparing with GC-MS literature data at NIST and TMS database Wiley/NIST.1998.1 [45].

Statistical Analysis

All calculated data was analysed by one way analysis of variance (ANOVA), mean difference between treatments was calculated for significance test by Duncan Multiple Range Test “DMRT ” at $P> 0.05$) with IBM-SPSS statistics 25.0 version software. Probit analysis was performed using EPA Probit analysis program version 1.5.

Results

The current study carried out on the flower’s extract of *Parthenium hysterophorus* revealed the existence of medicinally and agro-chemically active compounds. The phytochemical constituents of *P. hysterophorus* were qualitatively analyzed for antioxidant and insecticidal activities.

Extract Yield (%)

Yield of extract by the solvent extraction technique from *P. hysterophorus* flowers was calculated and presented in (Figure 1). The maximum extract was obtained by ethanol (4.34%) whereas; minimum extract quantity (2.39%) was afforded by dichloromethane. The extract obtained via ethanol and ethyl acetate was bluish green and greenish in color and exhibited consistency, respectively. The same oil was brownish in color and non-consistent via dichloromethane and distilled water, respectively. (Table 1).

Analysis for Phenol and Flavonoid and Antioxidant Activity

The total contents for phenol and flavonoid as well as DPPH scavenging activity afforded by *P. hysterophorus* flower’s extract via different solvents are presented in (Table 2). Results confirmed that the highest total phenol were reported from distilled water 103.30 GAE/g followed by Ethyl acetate and Ethanol’ extract, 47.79 and 27.55 GAE/g, respectively. On the other hand highest flavonoid was reported from Ethyl acetate extract 138.67 QE/g followed by Ethanol 14.23 QE/g respectively.

Inhibition (%) was determined by 1,1-Diphenyl-1-picrylhydrazyl (DPPH) displayed color absorption at 517nm on using spectrophotometer. When DPPH trap free radicals, the color of the solution changed to light yellow and, subsequently, resulted in decrease of the absorbance. Maximum DPPH radical scavenging activity was afforded by Dichloromethane extract 95.03% followed by Ethanol and Ethyl acetate 88.53% and 67.21%, respectively.

Correlation of Antioxidant Activity versus Phenols, Flavonoids and extract yield

In our study, the Pearson's correlation regarding antioxidant activity of *P. hysterophorus* showed positive relationship (Table 3). Results showed highly significant ($P<0.01$) negative effect of phenol with DPPH as well as DPPH showed strongly highly significant negatively correlation and similar results were found reciprocally in the studied parameters. However, yield extracted by different solvents did not affect the Antioxidant Activity versus phenols, flavonoids quantitatively.

Assessment of Insecticidal Activity

The mortality data of cabbage aphid *B. brassicae* at specific period of time using *P. hysterophorus* flower's extract is presented in (Figure 2). It was perceived that the percent mortality was directly correlated to the concentration and the time. The results demonstrated that the maximum mortality was recorded at 1000 µgmL^{-1} at 96 h and 72 h with 63.33% and 46.67%, respectively. Same trend of mortality was also observed at 800 µgmL^{-1} with 56.67% and 36.67%, respectively. Imidacloprid 25% WP being a positive control produced the highest rates of mortality after 96 h of exposure 97.67%. Likewise, Imidacloprid also produced significant mortality after 72 h and 48 h exposure 91.82% and 87.38% mortality, respectively.

Analysis for probability of the data showed the LC_{50} values, slope, chi-square, and fiducial limits at confidence interval of 95% for *P. hysterophorus* extracts. Mortality response using plant extracts showed sensitivity of *B. brassicae* to various concentrations which is represented in (Table 4).

Chemical Analysis

The existence of chemical compounds in *P. hysterophorus* flower's extract was analyzed by GCMS techniques and data is presented in (Table 5). Thirteen biochemical compounds equivalent to 99.99% of entire extract by GC fraction were identified. Moreover,2-naphthalenemethanol,decahydro-.alpha.,.alpha.,4a-trimethyl-8-methylene-, [2R--(2.alpha., 4a.alpha.,8a.beta.)]-(25.93%), histidine, 1,N-dimethyl-4-nitro- (19.25%) and 3-methoxy-3, 4-dimethyl-1-heptyne (11.27%), were the major chemical compounds while, 10 others chemical compounds were identified in minor amounts having areas with peak range 2.04–3.7%. GC-MS chromatogram of *P. hysterophorus* flower extract is presented in (Figure 3).

Discussion

The chemical compounds include phenolic, steroid, alkaloids, flavonoids and terpenoids depends their concentration on ecological conditions and occurrence of the pathogens. They also possessed bio-pesticidal properties and are target specific against insect pests and pathogens. Moreover, these compounds are biodegradable, without triggering severe harm to the concerns of the environment [27, 46-48].

Appraisal of antioxidant and polyphenols activity of medicinal products has become vital device to recognize the medicinal possessions of natural plants. An antioxidant is a molecule which exhibited the ability to inhibit the oxidation of other chemical elements. Kumar and Mishra [44] reported that alkaloids, flavonoids, terpenoids, and cardiac glycosides from the extract of *P. hysterophorus*. However, the

phenolic contents in flower extracts were ranging from 86.69-320.17 mg propyl gallate equivalent (PGE)/g. Study established that bio-chemicals possessed by *P. hysterophorus* extracts bears significant antioxidant activity consistent to our current findings. Similarly, 50% radical scavenging potential of *P. hysterophorus* extract at 250 μgmL^{-1} using DPPH assay as well as 30% inhibition at 100 μgmL^{-1} via hydrogen per oxide assays was reported from *P. hysterophorus* leaves extract [49]. Antioxidant activities of hydro-ethanolic extracts of *P. hysterophorus* leaves was perhaps because of the existence of polyphenols contents [11, 50].

Likewise, Sinha and Paul [51] found that extract from *P. hysterophorus* possess valuable antioxidant activity with 54.5% scavenging property and 60.2 IC_{50} via DPPH assay while, 93.2% and 33.6 IC_{50} via ABTS assay which are consistent with our findings on antioxidant activity. Bashir [52] evaluated leaf extract of *P. hysterophorus* for the presence of total phenolic and flavonoid contents as 105.44mg GAE/g and 41.50mg RE/g respectively exhibited by 60% ethanolic extract with IC_{50} value of 87.55 μgmL^{-1} and 98.22 μgmL^{-1} for DPPH radical scavenging activity respectively supported our investigations on antioxidant as well as phenol and flavonoid contents from *P. hysterophorus*. Phytochemical profile of *P. hysterophorus* root, stem, leaf and phyllary identified terpenes, fatty acids, hydrocarbons and phytosterols however; root extracts showed highest antioxidant potential [53]. The results obtained on antioxidant activity of *P. hysterophorus* are in agreement with Sinha and Paul [51] who reported that its leaves crude extract bears considerable antioxidant activity being IC^{50} 60.24 and 33.60% by DPPH and ABTS method respectively.

As it is evident from previous findings that exposure of natural fauna to harmful chemicals or particle can altered the biological activities, consequently, sometimes estrogen compounds cause acute toxicity in edible crops and vegetables which may

occur via long-term application of estrogen-containing irrigation water. Comparable results were reported by Adeel, Zain [54] that uptake and accumulation of l7β-estradiol (17β-E2) and ethinylestradiol (EE2) in lettuce (*Lactua sativa*) grown under lab conditions.

The historic practice of using nicotine and pyrethrum for pest control measures has encouraged scientists to draw their attention to secondary metabolites like terpenoids, alkaloids, and flavonoids as pest regulator agents [55]. So, the outcomes of the current exploration are in agreement with other workers like [56] who evaluated different parts of *P. hysterophorus* extract against *Callosobruchus chinensis* and establish that 73.33% mortality was caused after 96 h exposure followed by 76.67% and 56.67% via inflorescence powder and stem powder respectively. Moreover, their leaves extract afforded repellent affects as 70–100% for oviposition performance of the *Aedes aegypti* adults. However, the diethyl ether extracts resulting in extreme repellency as 99.7%.

The insecticidal impact of plant extracts may attribute to the following belongings including repellency and stomach poisoning might decrease insect movement and also cause death through constriction of the insect spiracles [57]. A study reported the chemical examination of parthenium has specified that whole plant exhibited toxins called as sesquiterpene lactones responsible for insecticidal activities however; the major constituent of these toxins is parthenin derivatives. These derivatives showed insecticidal as well as nematicidal activity against the *Callosobruchus maculatus* adults and root knot nematode *Meloidogyne incognita*. Similarly, the pyrazoline was found highly effective being an insecticide, with LC_{50} value 32 mgl^{-1} with exposure of 72 h, and found extremely effective as nematicidal activity with LC_{50} values being 512 mgl^{-1} perceived for parthenin at 72 h exposure [37].

Parthenium *hysterophorus* extract used against mustard aphid, *Lipaphis erysimi* under field conditions exhibited incredible decrease of 29% of the infestation which may be because of the effect of phenolic acids [58]. Ahmad, Bagheri [53] reported the efficacy of *P. hysterophorus* extract with 10 and 20% concentration found 26.70% and 27% mortality at 21 day after treatment. Consequently, Wasu Yogesh H [59] in their study reported the activity of leaves and stem extract of *P. hysterophorus* against different larval instars of *Aedes aegypti*. Stored stock and freshly prepared solution caused 80% and 100% mortality, respectively with concentration prepared in 200g/500 ml. Another study reported that crude aqueous extracts of whole plant of *P. hysterophorus* caused a significant mortality with 73.33, 86.67 and 83.33% of *B. brassicae* as well as moderate mortality being 26.67, 46.67 and 63.33% of *Myzus persicae* at 10% concentration at 24, 48 and 72h exposure, respectively [55]. Consequently, Wasu Yogesh H [59] in a study, predicted that crude extracts from fresh leaves of *P. hysterophorus* showed significant effects against *Spodoptera littura* and *Spodoptera littorallis* with LC_{50} values as 7.96% and 8.14%, respectively.

Gas Chromatographic- Mass Spectrometric (GC-MS) analyses offered the existence of various phytochemicals in different parts of the plants. In a systematic chemical study Wang, Gan [60], reported that solvent fraction of *P. hysterophorus*, contained fourteen chemical compounds were plant phytosterols, stigmasterol, daucosterol, dtigmasterol-3-O-glucopyranoside, and Stigmasteryl-3β-arachidate. The other six isolated terpenoids were parthenin, oleanolic acid, oleanolic acid 28-O-β-D-glucopyranoside, and dichrocepholide D. Moreover, Białoń, Krzyśko-Łupicka [61] assessed the chemical composition of Lavender oil and found various types of terpenoids (monoterpene, monoterpenoids, sesquiterpene and sesquiterenoids) with valuable anti-bacterial as well as anti-fungal activities. Likewise, analytical aspects of

Satureja montana and tincture were evaluated by chromatographic techniques (HPTLC) which showed its richness in polyphenols, phenolic and flavonoids contents could indicates its valuable anti-microbial and antioxidant activities and also provides basis for further clinical studies [62]. However, for unknown compounds which are likely to be found in the extract/essential oil, it is simpler to start with the GC-MS, associated with (NIST) library will help to identify at least a part of compounds which are composing the extract and then it would be easier to separate and investigate the still unidentified compounds. Moreover, in a study, isolation and determination of phenolic compounds from fresh water algae *Cladofora glomerata* by using Clourimetric method, chromatographic and HPLC techniques and identified nine phenolic compounds along with phenolic acids and flavonoids [63].

Parthenin a chief component was found in profusion in leaf extracts of *P. hysterophorus* which was positively correlated with cytotoxicity but not with phytotoxicity. Bajwa, Weston [64] perceived two compounds like chlorogenic acid and ambrosin were positively correlated with germination inhibition however, among identified phytochemicals vatirenene was present in high concentration as 19.8% in root. In a study Koo, Thomas [65] reported the existence of vatirenene in breath test which may be the possible cause of aspergillosis. Moreover, caryophyllene oxide is considered as an effective fungicidal mediator [66], and creation of resistance in reproductive parts [67, 68]. Likewise, globulol displays strong antibacterial activity [69]. However, the chemical compounds assessed during our current investigations from flower's extract of *P. hysterophorus* responsible towards antioxidant and insecticidal activity.

Although different studies has been conducted on phytochemistery, pharmacology as well as other biological activities but, studies on antioxidant activity, insecticidal

activity against *B. brassicae* and chemical analysis of flower's extract is limited so for. The increasing interest for food, medical and agrochemical industries and to develop botanical based natural antioxidants led towards ne investigations. Thus, this research represents the first investigation on antioxidant and insecticidal activity of flower's extract of *P. hysterophorus* against *B. brassicae*.

Conclusions

Parthenium hysterophorus is a noxious weed but also possessed numerous beneficial aspects as medical and agrochemical industries. The flower's extract of *P. hysterophorus* identified significant antioxidant activities with appreciable amount of phenolic and flavonoid contents. Similarly, the extract displayed prominent insecticidal activity against *Brevicoryne brassicae* a notorious sucking pest worldwide. Toxicity results displayed that *P. hysterophorus* extracts showed LC_{50} values of 1446 and 750µgmL^{-1} at 72 and 96 h exposure, respectively showed its effectiveness against *B. brassicae* . Moreover, antioxidant activity of *P. hysterophorus* extracts displayed strong correlation versus phenols, flavonoids and extract yield. Chemical analysis revealed the existence of various chemical compounds accountable for biological activities. Therefore, extract of *P. hysterophorus* might be used as antioxidant and insecticidal purposes as an alternative to synthetic chemical agents. However, more detailed study is required on extraction and purification of bioactive compounds and their evaluation as biological perspectives.

Acknowledgements

The support and supervision provided by Professor Zumin Gu, Ji Mingshan and experimental guidelines of lab mates of Biopesticides Laboratory, Plant Protection College Shenyang is greatly acknowledged.

Conflicts of Interest: "The authors declare no conflict of interest."

Data Availability: All relevant data are within the manuscript

Funding: This research was supported by National Key Research & Development Program of China (Grant No. 0201300). However, the funders had no role in study design, data collection and analysis, decision to publish, or preparation of the manuscript. Moreover, No any support was received by any third party in conducting this research, analyzing the data or preparing the manuscript for submission

Author Contributions: Conceptualization, M.J. and Z.G.; methodology, M.A.; T.A.; A.J., S; A.S. and F.A.; software, X.B. and T.u.H; validation, Z.G. formal analysis, M.J., Y.L. and Z.G.; investigation, M.J. and Z.G; resources, Z.G.; data curation, M.A. Writing—original draft, M.A.; writing—review & editing, M.J. visualization, Z.G., supervision, Z.G. and M.J.; funding acquisition, Z.G.

All authors have read and agreed to the published version of the manuscript

References

1. Sukanya S, Sudisha J, Hariprasad P, Niranjana S, Prakash H, Fathima S. Antimicrobial activity of leaf extracts of Indian medicinal plants against clinical and phytopathogenic bacteria. Afr J. Biotechnol. 2009;8(23).

2. Adkins SW, Shabbir A, Dhileepan K. Parthenium weed: biology, ecology and management: CABI; 2018.

3. Adkins S, Shabbir A. Biology, ecology and management of the invasive parthenium weed (Parthenium hysterophorus L.). Pest Manage Sci. 2014;70(7):1023-9.

4. Ray P, Gour HN. Puja Ray and H.N. Gour (2012). Integrated Management of *Parthenium hysterophorus* L. (Asteraceae): A weed of worldwide significance. Ind J Mycol Pl Path. 2012;5:605–32.

5. Kapoor RT. Awareness related survey of an invasive alien weed, *Parthenium hysterophorus* L. in Gautam Budh Nagar district, Uttar Pradesh, India. J Agric Technol. 2012;8(3):1129-40.

6. Khan N, Bibi K, Ullah R. Distribution pattern and ecological determinants of an invasive plant *Parthenium hysterophorus* L., in Malakand division of Pakistan. J Mt Sci. 2020;17:1670-83.

7. Kushwaha VB, Maurya S. Biological utilities of Parthenium hysterophorus. J Appl Nat Sci. 2012;4(1):137-43.

8. Kaur M, Aggarwal NK, Kumar V, Dhiman R. Effects and management of Parthenium hysterophorus: A weed of global significance. Int Sch Res Notices. 2014;2014.

9. Javaid A, Jabeen T, Khan IH, Jabeen K, Akbar M. Herbicidal potential of *Alternaria citri* Ellis and Pierce metabolites against *Parthenium hysterophorus*. L. Allelopathy J. 2022;55(1):25-34.

10. Khan IH, Javaid A, Ahmad S. Metabolites of *Penicillium citrinum* as potent herbicides against parthenium weed. Pak J Weed Sci Res. 2021;28(1):77-85.

11. Raza S, Chaudhary A, Mumtaz M, Bashir S. Antioxidant, iron chelating, lipase inhibition activities and metabolite's prediction of hyrdoethanolic leaf extract of Conocarpus erectus. Food Res. 2019;4:482-7.

12. Seufi AM, Mohammed HA, Moussa FI, El-Din AAS, El-Saadani M, Taha TH, et al. Protective Effects of the Aqueous Extract of Black Mulberry Leaves, Morus nigra, on Chlorpyrifos Toxicity in Male Albino Rats. Jordan J Biol Sci. 2019;12(4).

13. Kapoor RT. Farmers'perception about *Parthenium hysterophorus* l.-a case study of handia and phulpur tehsils of allahabad district, uttar pradesh. 2016.

14. Picman AK, Elliott R, Towers G. Cardiac-inhibiting properties of the sesquiterpene lactone, parthenin, in the migratory grasshopper, Melanoplus sanguinipes. Can J Zool. 1981;59(2):285-92.

15. Lakshmi C, Srinivas C. Parthenium: A wide angle view. Indian J Dermatol Venereol Leprol. 2007;73(5):296.

16. Batish DR, Singh H, Kohli R, Saxena D, Kaur S. Allelopathic effects of parthenin against two weedy species, Avena fatua and Bidens pilosa. Environ Exp Bot. 2002;47(2):149-55.

17. Batish D, Singh H, Saxena D, Kohli R. Weed suppressing ability of parthenin-a sesquiterpene lactone from Parthenium hysterophorus. N Z Plant Prot. 2002;55:218-21.

18. Patel VS, Chitra V, Prasanna PL, Krishnaraju V. Hypoglycemic effect of aqueous extract of Parthenium hysterophorus L. in normal and alloxan induced diabetic rats. Indian J Pharmacol. 2008;40(4):183.

19. Selmi S, Rtibi K, Grami D, Sebai H, Marzouki L. Rosemary (Rosmarinus officinalis) essential oil components exhibit anti-hyperglycemic, anti-hyperlipidemic and antioxidant effects in experimental diabetes. Pathophysiology. 2017;24(4):297-303.

20. Asghari B, Zengin G, Bahadori MB, Abbas-Mohammadi M, Dinparast L. Amylase, glucosidase, tyrosinase, and cholinesterases inhibitory, antioxidant effects, and GC-MS analysis of wild mint (Mentha longifolia var. calliantha) essential oil: A natural remedy. Eur J Integr Med. 2018;22:44-9.

21. Borah A, Paw M, Gogoi R, Loying R, Sarma N, Munda S, et al. Chemical composition, antioxidant, anti-inflammatory, anti-microbial and in-vitro cytotoxic

efficacy of essential oil of Curcuma caesia Roxb. leaves: An endangered medicinal plant of North East India. Ind Crops Prod. 2019;129:448-54.

22. Manssouri M, Znini M, Majidi L. Studies on the antioxidant activity of essential oil and various extracts of Ammodaucus leucotrichus Coss. & Dur. Fruits from Morocco. J Taibah Uni Sci. 2020;14(1):124-30.

23. Zhang C, Zhao J, Famous E, Pan S, Peng X, Tian J. Antioxidant, hepatoprotective and antifungal activities of black pepper (Piper nigrum L.) essential oil. Food Chem. 2021;346:128845.

24. Dwivedi S, Dwivedi A, Dwivedi S. Folk lore uses of some plants by the tribes of Madhya Pradesh with special reference to their conservation. Ethnobot leafl. 2008;2008(1):105.

25. Shabbir A, Javaid A. Effect of aqueous extracts of alien weed Parthenium hysterophorus and two native asteraceous species on germination and growth of mungbean, Vigna radiata L. wilczek. J Agric Res. 2010;48(4):483-8.

26. Towers G, Subba Rao P, editors. Impact of the pan-tropical weed, Parthenium hysterophorus L. on human affairs. Proceedings of the first international weed control congress; 1992: Weed Science Society of Victoria Melbourne.

27. Espinosa-Rivero J, Rendón-Huerta E, Romero I. Inhibition of Helicobacter pylori growth and its colonization factors by Parthenium hysterophorus extracts. J Ethnopharmacol. 2015;174:253-60.

28. Tanvir R, Sajid I, Hasnain S. Screening of endophytic Streptomycetes isolated from Parthenium hysterophorus L. against nosocomial pathogens. Pak J Pharm Sci. 2013;26(2):277-83.

29. Deka B, Jiten M, Bhattacharyya P. Acaricidal activities of Parthenium hysterophorus L. against red spider mite, Oligonychus coffeae Nietner (Acarina: Tetranychidae) of tea. Int J Sci Res. 2013;4(5):2319-7064.

30. Krishnaveni M. Air pollution tolerance index and antioxidant activity of Parthenium hysterophorus. J Pharm Res. 2013;7(4):296-8.

31. Nabi SA, Kasetti RB, Sirasanagandla S, Tilak TK, Kumar MVJ, Rao CA. Antidiabetic and antihyperlipidemic activity of Piper longum root aqueous extract in STZ induced diabetic rats. BMC Complement Altern Med. 2013;13(1):37.

32. Panwar R, Sharma AK, Dutt D, Pruthi V. Phenolic acids from Parthenium hysterophorus: evaluation of bioconversion potential as free radical scavengers and anticancer agents. Advances in Bioscience and Biotechnology. 2015;6(01):11.

33. Anup R, Sainath P, Padma S, Sukumaran M. In vitro cytotoxic activity of methanolic extract of Parthenium hysterophorus flowers on MCF-7 and HeLa cell lines. Int J Curr Microbiol Appl Sci. 2015;4(8):710-5.

34. Kumar S, Nair G, Singh AP, Batra S, Wahab N, Warikoo R. Evaluation of the larvicidal efficiency of stem, roots and leaves of the weed, Parthenium hysterophorus (Family: Asteraceae) against Aedes aegypti L. Asian Pac J Trop Dis. 2012;2(5):395-400.

35. Kumar VP, Chauhan NS, Padh H, Rajani M. Search for antibacterial and antifungal agents from selected Indian medicinal plants. J Ethnopharmacol. 2006;107(2):182-8.

36. Jha U, Chhajed PJ, Oswal RJ, Shelke TT. Skeletal muscle relaxant activity of methanolic extract of Parthenium hysterophorus L. leaves in swiss albino mice. Int J Pharm Life Sci. 2011;2(11).

37. Datta S, Saxena DB. Pesticidal properties of parthenin (from Parthenium hysterophorus) and related compounds. Pest Manag Sci. 2001;57(1):95-101.

38. Bharadwaja S, Singh S, Moholkar VS. Design and optimization of a sono-hybrid process for bioethanol production from Parthenium hysterophorus. J Taiwan Inst Chem Eng. 2015;51:71-8.

39. Parashar V, Parashar R, Sharma B, Pandey AC. Parthenium leaf extract mediated synthesis of silver nanoparticles: a novel approach towards weed utilization. Dig J Nanomater Biostructures. 2009;4(1).

40. Rajiv P, Rajeshwari S, Venckatesh R. Bio-Fabrication of zinc oxide nanoparticles using leaf extract of Parthenium hysterophorus L. and its size-dependent antifungal activity against plant fungal pathogens. Spectrochimica Acta Part A: Mol Biomol Spectrosc. 2013;112:384-7.

41. Yang J, Jiang F, Ma C, Rui Y, Rui M, Adeel M, et al. Alteration of crop yield and quality of wheat upon exposure to silver nanoparticles in a life cycle study. J Agric Food Chem. 2018;66(11):2589-97.

42. Ahmad MA, Yuesuo Y, Ao Q, Adeel M, Hui ZY, Javed R. Appraisal of comparative therapeutic potential of undoped and nitrogen-doped titanium dioxide nanoparticles. Molecules. 2019;24(21):3916.

43. Javed R, Ahmad MA, Gul A, Ahsan T, Cheema M. Comparison of chemically and biologically synthesized nanoparticles for the production of secondary metabolites, and growth and development of plants. Compr Anal Chem. 2021;94:303-29.

44. Ahmed M, Ji M, Qin P, Gu Z, Liu Y, Sikandar A, et al. Determination of phytochemicals, antioxidant activity and biochemical composition of chinese

mugwort (*Artemisia argyi* l.) leaf extract from northeast China. Appl Ecol Environ Res. 2019;17(6):15349-62.

45. Sparkman OD. Identification of essential oil components by gas chromatography/quadrupole mass spectroscopy Robert P. Adams. J Am Soc Mass Spectrom. 2005;16(11):1902.

46. McConnachie A, Strathie L, Mersie W, Gebrehiwot L, Zewdie K, Abdurehim A, et al. Current and potential geographical distribution of the invasive plant Parthenium hysterophorus (Asteraceae) in eastern and southern Africa. Weed Res. 2011;51(1):71-84.

47. Narasimhan T, Murthy BK, Harindranath N, Rao PS. Characterization of a toxin fromParthenium hysterophorus and its mode of excretion in animals. J Bios. 1984;6(5):729-38.

48. Hasan N, Jain R. Biotoxicity of Parthenium hysterophorus extracts against Meloidogyne javanica and Helicotylenchus dihystera. Nematol Mediterr. 1984.

49. Kalaiselvi M, Subbaiya R, Selvam M. Synthesis and characterization of silver nanoparticles from leaf extract of Parthenium hysterophorus and its anti-bacterial and antioxidant activity. Int J Curr Microbiol Appl Sci. 2013;2(6):220-7.

50. Arshad M, Mumtaz MW, Chaudhary AR, Rashid U, Ali M, Mukhtar H, et al. Metabolite profiling of Cycas revoluta leaf extract and docking studies on alpha-glucosidase inhibitory molecular targets by phytochemicals. PakJ Pharm Sci. 2019;32(2 (Supplementary)):871-4.

51. Sinha SN, Paul D. Antioxidant potentials of parthenium hysterophorus L. leaf extracts. Sci Res J India. 2014;3(2):80-6.

52. Bashir S, Raza, S.A., Ahmad, M., Farhan, M., Ali, S. and William, J. Hydroethanolic leaf extract of Parthenium hysterophorus attenuates blood glucose in alloxan induced diabetic mice. Food Res 2021;5(1):72-7.

53. Ahmad J, Bagheri R, Bashir H, Baig MA, Al-Huqail A, Ibrahim MM, et al. Organ-specific phytochemical profiling and antioxidant analysis of Parthenium hysterophorus L. BioMed Res Int. 2018;2018.

54. Adeel M, Zain M, Fahad S, Rizwan M, Ameen A, Yi H, et al. Natural and synthetic estrogens in leafy vegetable and their risk associated to human health. Environ Sci Pollut Res. 2018;25(36):36712-23.

55. Rajiv P, Rajeshwari S, Yadav RH, Rajendran V. Vermiremediation: Detoxification of Parthenin toxin from Parthenium weeds. J Hazard Mater. 2013;262:489-95.

56. Tesfu F, Emana G. Evaluation of Parthenium hysterophorus L. powder against Callosobruchus chinensis L.(Coleoptera: Bruchidae) on chickpea under laboratory conditions. Afri J Agri Res. 2013;8(44):5405-10.

57. Shaheen FA, Khaliq A. Management of Pulse Beetle, Callosobruchus chinensis L.(Coleoptera: Bruchidae) in stored Chickpea using ashes, red soil powder and turpentine oil. Pak Entomol. 2005;27(2):19-24.

58. Yadav SK, Patel S. Insecticidal and repellent activity of some plant extracts against Myzus persicae (Sulzer) and Brevicoryne brassicae(Linnaeus). J Entomol Zool Stud. 2017;5(2):1434-143.

59. Wasu Yogesh H GD, Patel Sagar B. Larvicidal effects of *Parthenium hysterophorus* against polyphagus pests *Spodoptera Litura* and *Spodoptera littoralis*. Indian J Appl Res. 2019;9(2):1-19.

60. Wang M-H, Gan J-J, Chen W-H, Guan Y-L. Chemical Constituents of Parthenium hysterophorus. Chem Nat Comp. 2020;56:556-8.
61. Białoń M, Krzyśko-Łupicka T, Nowakowska-Bogdan E, Wieczorek PP. Chemical composition of two different lavender essential oils and their effect on facial skin microbiota. Molecules. 2019;24(18):3270.
62. Hudz N, Makowicz E, Shanaida M, Białoń M, Jasicka-Misiak I, Yezerska O, et al. Phytochemical Evaluation of Tinctures and Essential Oil Obtained from Satureja montana Herb. Molecules. 2020;25(20):4763.
63. Korzeniowska K, Łęska B, Wieczorek PP. Isolation and determination of phenolic compounds from freshwater Cladophora glomerata. Algal Res. 2020;48:101912.
64. Bajwa AA, Weston PA, Gurusinghe S, Latif S, Adkins SW, Weston LA. Toxic Potential and Metabolic Profiling of Two Australian Biotypes of the Invasive Plant Parthenium Weed (Parthenium hysterophorus L.). Toxins. 2020;12(7):447.
65. Koo S, Thomas HR, Daniels SD, Lynch RC, Fortier SM, Shea MM, et al. A breath fungal secondary metabolite signature to diagnose invasive aspergillosis. Clin Infect Dis. 2014;59(12):1733-40.
66. Yang D, Michel L, Chaumont J-P, Millet-Clerc J. Use of caryophyllene oxide as an antifungal agent in an in vitro experimental model of onychomycosis. Mycopathologia. 2000;148(2):79-82.
67. Saifullah K. Callus induction and cell suspension culture production of Catharanthus roseus for biotransformation studies of (-)-caryophyllene oxide. Pak J Bot. 2011;43(1):467-73.

68. Halim SA, Uddin R, Madura JD. Benchmarking docking and scoring protocol for the identification of potential acetylcholinesterase inhibitors. J Mol Graph Model. 2010;28(8):870-82.

69. Mulyaningsih S, Sporer F, Reichling J, Wink M. Antibacterial activity of essential oils from Eucalyptus and of selected components against multidrug-resistant bacterial pathogens. Pharm Biol. 2011;49(9):893-9.

Tables

Table 1. Physical appearance of the flower's extract of *Parthenium hysterophorus*.

Extract	Visibility	Physical Nature	Consistency
EtOH	Bluish green	Oily	Consistent
EA	Greenish	Oily	Consistent
DCM	Brownish	Oily/Gummy	Non-consistent
DW	Brownish	Oily/Gummy	Non-consistent

EtOH; (Ethanol), EA; (Ethyl Acetate), DCM; (Dichloromethane), DW; (Distilled water).

Table 2. Total Phenol, Flavonoid content and DPPH radical scavenging activity of the flower's extract of *Parthenium hysterophorus*.

Extracts	Phenol (GAE/g)	Flavonoid (QE/g)	DPPH Inhibition (%)
EtOH	27.55±1.46^{c}	14.23±0.10^{b}	88.53±0.11^{b}
EA	47.79±0.85^{b}	138.67±2.20^{a}	67.21±0.19^{c}
DCM	30.50±0.00^{d}	15.38±0.04^{c}	95.03±1.58^{a}
DW	103.30±1.16^{a}	4.81±0.01^{c}	18.73±0.89^{d}
Statics			
	S.S= 24268	S.S= 248063	S.S= 13880
	M.S=8069	M.S= 82687	M.S= 4626
	Df= 3	Df= 3	M.S= 3
	f= 2564***	*f*= 22488***	*f*= 1031***

Data in the columns is described as mean values ± standard deviations with various superscripts are significantly different according to DMRT" $P > 0.05$). S.S (Sum of square); M.S (Mean square); Df (Degree of freedom); *f* (Significance); *** (significance level).

Table 3. Correlation of the antioxidant activity of *P. hysterophorus* extract versus Phenols, Flavonoids and extract yield.

Treatments	Phenols	Flavonoids	DPPH	Yield
Phenols	1	-0.16	-0.99**	-0.11
Flavonoids	-0.16	1	-0.71	-0.90
DPPH	-0.99**	-0.71	1	-0.10
Yield	-0.11	-0.09	-0.10	1

Note: * Correlation is significant at 0.05 levels; ** Correlation is highly significant at 0.05 levels

Table 4. Toxicity of *P. hysterophorus* extracts against *B. brassicae* at 12, 24, 48, 72 and 96 hours exposure

Time	LC_{50} µgmL^{-1}	95% F.L		Slope± S.E	χ2
		Lower	Upper		
12	3598	1421	35512	1.09±0.38	0.25
24	2569	1321	27758	1.51±0.46	0.99
48	2345	1153	25517	1.31±0.53	0.85
72	1446	852	21721	1.56±0.63	0.22
96	750	468	1276	1.89±0.58	0.04

Note: LC_{50} (Lethal concentration); S.E. (standard error); χ2 (chi-square); F.L. (Fiducial limit)

Table 5. Chemical composition of the flower's extract of *Parthenium hysterophorus*.

Peak #	R. T	Area %	Chemical Compounds	M.F	M.W g/mol
1	3.22	7.05	Benzene, 1,3-dimethyl-	C_8H_{10}	106.17
2	3.92	3.47	2,4-Dimethylamphetamine	$C_{11}H_{17}N$	163.26
3	4.43	2.04	3-Piperidinol	$C_5H_{11}NO$	101.15
4	5.05	2.74	2-Pentene, 4,4-dimethyl-, (E)-	C_7H_{14}	98.186
5	5.52	3.55	2-Butanamine	C_6H_5N	101.19
6	12.96	25.93	2-Naphthalenemethanol,decahydro.alpha.,.α.,4a-trimethyl-8-methylene-,[2R (2 α.,4a.α.,8a.beta.)]-	$C_{15}H_{26}O$	222.36
7	14.29	11.27	3-Methoxy-3,4-dimethyl-1-heptyne	$C_{10}H_{18}O$	154.25
8	15.21	4.76	Undecane	$C_{11}H_{24}$	156.31
9	15.85	3.82	2-Pyrrolidinone, 1-methyl-	C_5H_9NO	99.13
10	16.62	7.26	Glycinamide hydrochloride	$C_2H_7C_lN_2O$	110.54
11	17.58	2.18	1,2-Benzenedicarboxylic acid, butyl 2-ethylhexyl ester	$C_{20}H_{30}O_4$	334.45
12	18.1	6.67	Hexadecanoic acid, ethyl ester	$C_{18}H_{36}O_2$	284.48
13	21.85	19.25	Histidine, 1,N-dimethyl-4-nitro-	$C_8H_{10}N_2O_2$	166.18

R.T (Retention time); M.F (Molecular formula); M.W (Molecular Weight).

Manuscript

Click here to access/download;Manuscript;Final version 27.02.23.docx

Original Manuscript

Antioxidant, Insecticidal Activity and Chemical Profiling of Flower's Extract of Parthenium weed (*Parthenium hysterophorus* L.)

Maqsood Ahmed[1,2], Ansar Javeed[3], Samiullah[2], Talfoor-ul-Hassan[4], Aatika Sikandar[1], Taswar Ahsan[5], Faqir Ahmed[6], Ji Mingshan[1], Xuejing Bai[7], Ying Li[8] and Zumin Gu[1,*]

[1]College of Plant Protection, Shenyang Agricultural University, Shenyang, 110866, P.R. China. aatika_sikander@yahoo.com (A.S.); jimingshan@syau.edu.cn (M.J.)
[2]Department of Agriculture (Plant Protection), Pest Warning and Quality Control of Pesticides, Gujrat, 50700, Pakistan. maqsoodahmed200@hotmail.com
[3]Henan University, Jinming Campus, Kaifeng City Henan China. ansarjaveed@henu.edu.cn (A.J)
[4]Department of Agriculture (Plant Protection), Pest Warning and Quality Control of Pesticides, Gujranwala, 52250, Pakistan. ddappgujranwala@gmail.com (T.u.H.)
[5]Institute of Plant Protection Liaoning Academy of Agricultural Sciences, Shenyang, 110866, P.R. China. taswarahsan@163.com (T.H)
[6]Directorate General of Agriculture, Pest Warning and Quality Control of Pesticides, Lahore, 54800, Pakistan. faqirahmad63@yahoo.com (F.A)
[7]Shenyang Academy of Landscape- Gardening Shenyang 110016, P.R. China. baixuejingnv@163.com (X.B.)
[8] Fushun Modern Agriculture and Poverty Alleviation Development Promotion Centre, Fushun 113006 P.R. China. liying_1973@163.com (Y.L.)

Corresponding author: *guzumin1212@163.com *(G.Z); Tel: +86-24- 8848 - 7148; Fax: +86-24-8834-2315*

Abstract

Parthenium hysterophorus L. an invasive alien species is considered a noxious weed and confers numerous benefits in medical and agrochemical industries. The present study evaluated the flower extract's antioxidant and insecticidal activity and chemical profiling to appraise the existence of phytoconstituents responsible for biological activities. Assay was performed by 1,1-diphenyl-2-picrylhydrazyl (DPPH) for antioxidant activity, whereas, chemical configuration was evaluated by Gas Chromatography Mass Spectrometry (GCMS) analysis. Our findings report that the extract of *P. hysterophorus* possesses potential radical scavenging activity (95.03%) when extracted via dichloromethane (DCM). Additionally, phytochemical analysis revealed a considerable amount of phenols and flavonoid contents when extracted via distilled water and ethyl acetate; (103.30 $GAEg^{-1}$ and 138.67 $QE\text{-}g^{-1}$) respectively. Maximum mortality 63.33% and 46.67% was observed at 1000 μgmL^{-1} and 800 μgmL^{-1} after 96 h exposure followed by 46.67% and 36.67% at 72 h at the same concentration. On the other hand, toxicity results displayed that *P. hysterophorus* extracts showed LC_{50} values of 1446 and 750 μgmL^{-1} at 72 and 96 h exposure, respectively. However, Imidacloprid being a positive control afforded maximum mortality at 96 h and 72 h exposure 97.67% and 91.82%, respectively. Moreover, antioxidant activity of *P. hysterophorus* extracts displayed strong correlation versus phenols, flavonoids and extract yield. GCMS analysis revealed the existence of 13 chemical compounds corresponding to 99.99% of whole extract. Moreover, maximum extract was afforded by Ethanol followed by distilled water, ethyl acetate and dichloromethane as 4.34, 3.22, 3.17 and 2.39%, respectively. *P. hysterophorus* flower's extract therefore showed good antioxidant and aphicidal activity which also exhibited valuable chemical compounds accountable for biological activity, making them suitable candidates for an alternative to synthetic agents. However, our results provide novel and fundamental basis for further exploration on purification of chemical compounds for their biological activities.

Keywords: *Parthenium hysterophorus*; phytochemicals; Antioxidant activity; GCMS; DPPH

Introduction

The invasive alien species exert devastating impacts on inherent biota and deleteriously affecting ecosystems by destructions of native species. Likewise, such distressing impacts on natural biodiversity could lead towards escalation of climate change, environmental pollution, and ecological destruction of natural habitat. However, identifying the beneficial and harmful impacts of *Parthenium hysterophorus* introduced as an invasive alien species, might lead towards control of biological invasions. Invasive alien plant species are the worldwide risk to biodiversity, functioning of ecosystem and farm production [1]. Invasive weeds species are undesirable self-grown plants that compete with the cultivated and non-cultivated crops for nutrients, sunlight and water. They also produce a wide range of bioactive particles that are rich sources of various types of medicines. Currently, most of the medications in the pharmaceutical industry depend upon natural plants and or in the herbal traditional systems [1].

Parthenium hysterophorus L. is an extremely invasive plant species belonging to family Asteraceae that has invaded almost fifty countries in sub-continent Africa, Asia, and Oceania, infesting forests, feeding lands, and urban landscapes [2, 3]. It is a devastating and hazardous weed of various economically important crops [4]. Moreover, it is amongst the top ten worst weeds in the world and is included in the world catalogue of destructive species [5, 6]. In Europe *P. hysterophorus* is considered as an ephemeral species. It is generally recognized by different names such as parthenium weed, congress grass, Gajjar botti, white head and white top [7]. It is native to Gulf of Mexico and United States. However, it accidently introduced in Indian subcontinent [8]. This weed has been considered as a major weed of high rank

spreading at a much faster pace to takeover abandoned land, canal, watercourse banks, roads, and fields to challenge crop productivity [9, 10].

Medicinal plants are the fundamentals of traditional therapeutic systems based on the trusts and observations comprised of hundreds of years [11]. The phytochemical constituents such as phenolic and flavonoid are secondary metabolites involved in therapeutic system, because of the existence of these functional phytochemicals mainly parthenin [12]. Moreover, *P. hysterophorus* renowned to contain vital biochemical compounds such as alkaloids, tannins, and saponins, which are important from the medicinal viewpoint [13]. It also exhibited allelopathic effects and important phytochemicals such as parthenon and coronopilin which are auto-toxic to its own seed germination and seedling growth [14]. Furthermore, serious health consequences on human beings and livestock have been reported [15]. The agriculturists who are come in contact with *P. hysterophorus*; the pollen and dust produced by this weed provoke allergic contact dermatitis in human beings. Major chemical compounds, parthenin's contained by *P. hysterophorus* exhibited phytotoxic properties against a variety of plants, including weeds and various crops such as Slender amaranth (*Amaranthus viridis*), Billygoat weed (*Ageratum conyzoides*), common wild oat. (*Avena fatua*), nettleleaf goosefoot (*Chenopodium murale*), Foetid cassia (*Cassia tora*), mung bean *Phaseolus aureus* [16, 17]. Moreover, parthenin's the main biochemical compounds showed insecticidal activity against termites, cockroaches and migratory grasshoppers, *Melanoplus sanguinipes* [7]. Similarly, Patel, Chitra [18] reported pesticidal activity of the ethanol extract of *P. hysterophorus* which showed substantial lethal effect against stored grain pest like *Tribolium* sp. and *Oryzaephilus* sp. Essential oils from the natural plants also have antioxidant effects. In recent years several reports have been breakout, the essential oil from Rosemary [19], wild mint

(*Mentha longifolia* var. *Calliantha*) [20], black turmeric (*Curcuma caesia*) [21] *Ammodaucus leucotrichus* [22] and black pepper (*Piper nigrum*) [23].

Pharmacologically, it is involved in treating numerous diseases like allergies, anemia, sores, skin diseases, facial neuralgia, fever, vermifuge, blood purifier, and insecticide [7, 24]. This plant species is enriched with vital phytoconstituents however; sesquiterpene lactones are the most copious phytochemicals exhibited by this plant which are accountable for injurious effects such as strong allelopathic [25], toxic to livestock [26], It also possess many advantageous effects like antibacterial [27, 28], acaricidal [29], antioxidant [30, 31], cytotoxic [32, 33], larvicidal potential against *Aedes aegypti* L. [34, 35], skeletal muscle relaxant [36], pesticidal agent [37], producing bioethanol [38], as reducing agent in the formation of silver nanoparticles [39] and in production of zinc nanoparticles [40]. However, various factors affect the biological profile of the plants and from some recent studies it has been reported that nanoparticle influenced the physiological and biological properties of the plants. It has been reported that metal-based nanoparticle showed a decrease in protein and amino acid and micronutrient contents in wheat crop grown under such conditions [41]. Moreover, nitrogen doped titanium dioxide nanoparticle enhanced antioxidant activity via reducing power assay and percent inhibition of DPPH against *Streptomyces* [42]. Likewise, synthetically and biologically prepared nanoparticle significantly exerted effects on plant growth and secondary metabolites which ultimately triggered the biological activity of the plants [43].

As some earlier researches have been conducted on this plant species but, detailed studies on chemical and quantitative phytochemical analysis and assessment of antioxidant activities from different solvents extract as well as aphicidal activity against cabbage aphid *B. brassicae* from flower's extracts has not been explored

before. Hence, keeping in view the diverse biological properties of *P. hysterophorus*, current study was designed to determine the antioxidant and insecticidal activity of flower's extract against *B. brassicae* with appraisal of quantitative phytochemical analysis as well as assessment of chemical profiling of extract.

Materials and Methods

Collection and Samples Preparation

The whole plant samples were collected from District Sialkot Punjab Pakistan during the month of June-July 2019 and were identified as *Parthenium hysterophorus* L., from Ayub Agricultural Research Institute (AARI) Faisalabad Pakistan by Dr. Dilbar Hussain. Flowers were manually separated from the plants and left over for 20 days to dry under shade. These dried flowers were powdered by electric grinder and then extracted with different solvents (ethanol, ethyl acetate, dichloromethane and distilled water) by cold extraction method for seventy two hours at fixed temperature 28°C and at 100 rpm in an incubator shaker (ZWY-1102C). Extracted contents were filtered and concentrated to lessen the volume on rotary evaporator model R-210 BUCHI Labortechnik AG, CH-9230 Flawil 1/Switzerland). Obtained extract's yield was measured using following Equation (Eq. 1).

$$\text{Yield (\%)} = \frac{\text{Weight of the extract}}{\text{Weight of the dried sample}} \times 100 \qquad \text{(Eq. 1)}$$

Quantitative Analysis for Total Phenolic and Flavonoids Content

To assess the phytochemicals quantitatively, 1 mg of extract from each solvent was dissolved in 1 mL Methanol separately. Consequent mixture was vortexed and separated for analysis. For the assessment of total content for phenols Folin–Ciocalteu reagent test was used. In brief, 1 mL solution (1 $mgmL^{-1}$) was added to 2.5 mL of (10%) Folin–Ciocalteu with supplementary addition of 2 mL solution of (2%) sodium carbonate (Na_2CO_3). The consequential blend was incubated for fifteen min in the

dark to measure absorbance at 765 nm in 96-well ELISA plate using (SpectraMax 190, Meigu molecular International Co. Ltd., Shanghai China). To build a standard curve, Gallic acid was used (1 $mgmL^{-1}$) at different concentrations such as 1, 0.50, 0.25, 0.10, 0.05, 0.02, 0.01, 0 mg mL^{-1} to describe the obtained results as Gallic acid equivalent (GAE) mg g^{-1} of total extract. Each treatment was replicated ten times to analyze the data.

To examine the total flavonoids content, aluminium chloride colourimetric method was adopted. Briefly, 1 mL solution (1$mgmL^{-1}$) was poured into 3 mL methanol, with addition of 0.2 mL of 1 M Potassium acetate (CH_3COOK), 0.2 mL of (10%) aluminium chloride ($AlCl_3$) and at the end, distilled water 5.6 mL was added to the consequent mixture. The incubation of solution was performed for half an hour in the darkness and absorbance at 420 nm was measured. A standard curve was built using quercetin at different concentrations such as 1, 0.50, 0.25, 0.10, 0.05, 0.02, 0.01, 0 $mgmL^{-1}$ to represent the results as quercetin equivalent (QE) mg g^{-1} of total extract. The procedure was replicated 10 times for each treatment.

Assessment of Free Radical Scavenging Activity

Antioxidant activity of extracts obtained by different solvents from *Parthenium hysterophorus* flowers was accomplished in 1% solution of tween 20 by using 1,1-diphenyl-2-picrylhydrazyl (DPPH) ($C_{18}H_{13}N_5O_6$) a free radical. In brief, to assess the free radical scavenging activity, all dried solvent extracts were mixed together in equal proportion (5 mg each) and were dissolved in HPLC grade methanol and filtered. The filtrate was formerly used to assess the antioxidant activity. Similarly, DPPH solution was prepared in same grade Methanol @ 0.001g 25 mL^{-1}. Then, into 3.5 mL freshly prepared DPPH solution and 0.25 mL extract prepared in Methanol was added. The consequent mixture was shacked for complete mixing and then the

resulting mixture was incubated in darkness at 28°C for 30 min [44]. After incubation of resulting mixture for stipulated period of time, the absorbance was assessed at 517nm by using (SpectraMax 190, Meigu molecular International Co. Ltd., Shanghai China). The inhibition percentage of primed DPPH solution was predicted on reduction of absorbance through following Equation (Eq. 2). Each treatment was replicated ten times to analyze the data.

$$\text{Inhibition (\%)} = \frac{A_{\text{blank}} - A_{\text{sample}}}{A_{\text{blank}}} \times 100 \qquad \text{(Eq. 2)}$$

Where: A_{blank} = (control absorbance); A_{Sample} = (samples absorbance).

Correlation of Antioxidant Activity versus Phenols, Flavonoids and extract yield

Correlation of antioxidant activity *P. hysterophorus* versus Phenols, Flavonoids and extract yield was carried out using SPSS statistics 25.0 version with significant values at ($P>0.05$) level.

Assessment of Insecticidal Activity

Residual toxicity method was adopted for the assessment of insecticidal activity of extract of *P. hysterophorus* flowers. Samples for bioassay study were prepared to obtain mother mixture by mixing 0.1g of each solvent's extract and dissolved in Acetone, because it is aprotic solvent having intermediate polarity comparable to polar parotic solvents and it also evaporates immediately from the extract when exposed to air. The resulting mixture was placed in the fume hood for six hours for complete drying of Acetone. Serial concentrations *viz*. 100, 200, 400, 800 and 1000 µg mL^{-1} was prepared in Tween-20 (1%) solution from mother mixture. Briefly, fresh cabbage leaf discs 5cm in diameter were cut off and dipped for 10 s in the respective concentration and then dried for 5 min at room temperature. Formerly, 10 adult wingless aphids were carefully released onto the leaf disc using a fine-haired brush contained in the Petri dish and incubated for 96 h at 65% R.H., 25°C and with a 16:8

(light: dark) photoperiod. Imidacloprid 25% WP was used as the positive control at a rate of 0.0025 mL mL^{-1} of water, and control (CK) with a 1% Tween 20 solution. Positive controls and CKs were placed in a greenhouse for 96 h. Mortality data was collected at 12, 24, 48, 72 and 96 h using binocular microscope and the response of aphids was observed by needle probing, and the aphids who offered no response on stimulation were considered dead. Each treatment was replicated five times.

Chemical Analysis

Samples for analysis were prepared by mixing 0.1 g of each solvent extract into 2 mL of HPLC grade methanol to obtain mother solution. Consequent mixture was vortexed for 1 min following the addition of graphitized carbon black “GCB” (0.005 g). Mixture was centrifuged for 10 min at 10000 rpm at 25°C and colorless material was separated and collected in (1.5 mL) centrifuges tubes. Work for GC-MS analysis was accomplished using an Agilent Model 6890-5973N accompanied with gas chromatograph established on HP1 capillary column TG-5MS polydimethylsiloxane (length 30m × diameter 250 μm × film thickness 0.25 μm) interfaced with mass selective detector Hewlett Packard (5973N). The established parameters were; preliminary temperature was adjusted at 70°C for 0 min however, final temperature was extended to 200 °C for 10°C min^{-1} whereas, inlet temperature was adjusted at 250°C with split ratio 10:1. MS quadruple temperatures were fixed at 150°C while thermal aux temperatures were 285°C. The Scan range for MS was 35–520 units however; as a carrier, helium gas was used bearing stream frequency of $1.0 mLmin^{-1}$. The existing compounds in the extract were identified by comparison of their retention time and the mass spectra of the NIST 08 and Wiley 7 of the data base and confirmed by comparing with GC-MS literature data at NIST and TMS database Wiley/NIST.1998.1 [45].

Statistical Analysis

All calculated data was analysed by one way analysis of variance (ANOVA), mean difference between treatments was calculated for significance test by Duncan Multiple Range Test "DMRT " at $P > 0.05$) with IBM-SPSS statistics 25.0 version software. Probit analysis was performed using EPA Probit analysis program version 1.5.

Results

The current study carried out on the flower's extract of *Parthenium hysterophorus* revealed the existence of medicinally and agro-chemically active compounds. The phytochemical constituents of *P. hysterophorus* were qualitatively analyzed for antioxidant and insecticidal activities.

Extract Yield (%)

Yield of extract by the solvent extraction technique from *P. hysterophorus* flowers was calculated and presented in (Figure 1). The maximum extract was obtained by ethanol (4.34%) whereas; minimum extract quantity (2.39%) was afforded by dichloromethane. The extract obtained via ethanol and ethyl acetate was bluish green and greenish in color and exhibited consistency, respectively. The same oil was brownish in color and non-consistent via dichloromethane and distilled water, respectively, (Table 1).

Analysis for Phenol and Flavonoid and Antioxidant Activity

The total contents for phenol and flavonoid as well as DPPH scavenging activity afforded by *P. hysterophorus* flower's extract via different solvents are presented in (Table 2). Results confirmed that the highest total phenol were reported from distilled water 103.30 GAE/g followed by Ethyl acetate and Ethanol' extract, 47.79 and 27.55 GAE/g, respectively. On the other hand highest flavonoid was reported from Ethyl acetate extract 138.67 QE/g followed by Ethanol 14.23 QE/g respectively.

Inhibition (%) was determined by 1,1-Diphenyl-1-picrylhydrazyl (DPPH) displayed color absorption at 517nm on using spectrophotometer. When DPPH trap free radicals, the color of the solution changed to light yellow and, subsequently, resulted in decrease of the absorbance. Maximum DPPH radical scavenging activity was afforded by Dichloromethane extract 95.03% followed by Ethanol and Ethyl acetate 88.53% and 67.21%, respectively.

Correlation of Antioxidant Activity versus Phenols, Flavonoids and extract yield

In our study, the Pearson's correlation regarding antioxidant activity of *P. hysterophorus* showed positive relationship (Table 3). Results showed highly significant ($P<0.01$) negative effect of phenol with DPPH as well as DPPH showed strongly highly significant negatively correlation and similar results were found reciprocally in the studied parameters. However, yield extracted by different solvents did not affect the Antioxidant Activity versus phenols, flavonoids quantitatively.

Assessment of Insecticidal Activity

The mortality data of cabbage aphid *B. brassicae* at specific period of time using *P. hysterophorus* flower's extract is presented in (Figure 2). It was perceived that the percent mortality was directly correlated to the concentration and the time. The results demonstrated that the maximum mortality was recorded at 1000 µgmL^{-1} at 96 h and 72 h with 63.33% and 46.67%, respectively. Same trend of mortality was also observed at 800 µgmL^{-1} with 56.67% and 36.67%, respectively. Imidacloprid 25% WP being a positive control produced the highest rates of mortality after 96 h of exposure 97.67%. Likewise, Imidacloprid also produced significant mortality after 72 h and 48 h exposure 91.82% and 87.38% mortality, respectively.

Analysis for probability of the data showed the LC_{50} values, slope, chi-square, and fiducial limits at confidence interval of 95% for *P. hysterophorus* extracts. Mortality response using plant extracts showed sensitivity of *B. brassicae* to various concentrations which is represented in (Table 4).

Chemical Analysis

The existence of chemical compounds in *P. hysterophorus* flower's extract was analyzed by GCMS techniques and data is presented in (Table 5). Thirteen biochemical compounds equivalent to 99.99% of entire extract by GC fraction were identified. Moreover,2-naphthalenemethanol,decahydro-.alpha.,.alpha.,4a-trimethyl-8-methylene-, [2R--(2.alpha., 4a.alpha.,8a.beta.)]-(25.93%), histidine, 1,N-dimethyl-4-nitro- (19.25%) and 3-methoxy-3, 4-dimethyl-1-heptyne (11.27%), were the major chemical compounds while, 10 others chemical compounds were identified in minor amounts having areas with peak range 2.04–3.7%. GC-MS chromatogram of *P. hysterophorus* flower extract is presented in (Figure 3).

Discussion

The chemical compounds include phenolic, steroid, alkaloids, flavonoids and terpenoids depends their concentration on ecological conditions and occurrence of the pathogens. They also possessed bio-pesticidal properties and are target specific against insect pests and pathogens. Moreover, these compounds are biodegradable, without triggering severe harm to the concerns of the environment [27, 46-48].

Appraisal of antioxidant and polyphenols activity of medicinal products has become vital device to recognize the medicinal possessions of natural plants. An antioxidant is a molecule which exhibited the ability to inhibit the oxidation of other chemical elements. Kumar and Mishra [44] reported that alkaloids, flavonoids, terpenoids, and cardiac glycosides from the extract of *P. hysterophorus*. However, the

phenolic contents in flower extracts were ranging from 86.69-320.17 mg propyl gallate equivalent (PGE)/g. Study established that bio-chemicals possessed by *P. hysterophorus* extracts bears significant antioxidant activity consistent to our current findings. Similarly, 50% radical scavenging potential of *P. hysterophorus* extract at 250 μgmL^{-1} using DPPH assay as well as 30% inhibition at 100 μgmL^{-1} via hydrogen per oxide assays was reported from *P. hysterophorus* leaves extract [49]. Antioxidant activities of hydro-ethanolic extracts of *P. hysterophorus* leaves was perhaps because of the existence of polyphenols contents [11, 50].

Likewise, Sinha and Paul [51] found that extract from *P. hysterophorus* possess valuable antioxidant activity with 54.5% scavenging property and 60.2 IC_{50} via DPPH assay while, 93.2% and 33.6 IC_{50} via ABTS assay which are consistent with our findings on antioxidant activity. Bashir [52] evaluated leaf extract of *P. hysterophorus* for the presence of total phenolic and flavonoid contents as 105.44mg GAE/g and 41.50mg RE/g respectively exhibited by 60% ethanolic extract with IC_{50} value of 87.55 μgmL^{-1} and 98.22 μgmL^{-1} for DPPH radical scavenging activity respectively supported our investigations on antioxidant as well as phenol and flavonoid contents from *P. hysterophorus*. Phytochemical profile of *P. hysterophorus* root, stem, leaf and phyllary identified terpenes, fatty acids, hydrocarbons and phytosterols however; root extracts showed highest antioxidant potential [53]. The results obtained on antioxidant activity of *P. hysterophorus* are in agreement with Sinha and Paul [51] who reported that its leaves crude extract bears considerable antioxidant activity being IC^{50} 60.24 and 33.60% by DPPH and ABTS method respectively.

As it is evident from previous findings that exposure of natural fauna to harmful chemicals or particle can altered the biological activities, consequently, sometimes estrogen compounds cause acute toxicity in edible crops and vegetables which may

occur via long-term application of estrogen-containing irrigation water. Comparable results were reported by Adeel, Zain [54] that uptake and accumulation of l7β-estradiol (17β-E2) and ethinylestradiol (EE2) in lettuce (*Lactua sativa*) grown under lab conditions.

The historic practice of using nicotine and pyrethrum for pest control measures has encouraged scientists to draw their attention to secondary metabolites like terpenoids, alkaloids, and flavonoids as pest regulator agents [55]. So, the outcomes of the current exploration are in agreement with other workers like Tesfu and Emana [56] evaluated different parts of *P. hysterophorus* extract against *Callosobruchus chinensis* and establish that 73.33% mortality was caused after 96 h exposure followed by 76.67% and 56.67% via inflorescence powder and stem powder respectively. Moreover, their leaves extract afforded repellent affects as 70–100% for oviposition performance of the *Aedes aegypti* adults. However, the diethyl ether extracts resulting in extreme repellency as 99.7%.

The insecticidal impact of plant extracts may attribute to the following belongings including repellency and stomach poisoning might decrease insect movement and also cause death through constriction of the insect spiracles [57]. A study reported the chemical examination of parthenium has specified that whole plant exhibited toxins called as sesquiterpene lactones responsible for insecticidal activities however; the major constituent of these toxins is parthenin derivatives. These derivatives showed insecticidal as well as nematicidal activity against the *Callosobruchus maculatus* adults and root knot nematode *Meloidogyne incognita*. Similarly, the pyrazoline was found highly effective being an insecticide, with LC_{50} value 32 mgl^{-1} with exposure of 72 h, and found extremely effective as nematicidal activity with LC_{50} values being 512 mgl^{-1} perceived for parthenin at 72 h exposure [37].

Parthenium *hysterophorus* extract used against mustard aphid, *Lipaphis erysimi* under field conditions exhibited incredible decrease of 29% of the infestation which may be because of the effect of phenolic acids [58]. Ahmad, Bagheri [53] reported the efficacy of *P. hysterophorus* extract with 10 and 20% concentration found 26.70% and 27% mortality at 21 day after treatment. Consequently, Wasu Yogesh H [59] in their study reported the activity of leaves and stem extract of *P. hysterophorus* against different larval instars of *Aedes aegypti*. Stored stock and freshly prepared solution caused 80% and 100% mortality, respectively with concentration prepared in 200g/500 ml. Another study reported that crude aqueous extracts of whole plant of *P. hysterophorus* caused a significant mortality with 73.33, 86.67 and 83.33% of *B. brassicae* as well as moderate mortality being 26.67, 46.67 and 63.33% of *Myzus persicae* at 10% concentration at 24, 48 and 72h exposure, respectively [55]. Consequently, Wasu Yogesh H [59] in a study, predicted that crude extracts from fresh leaves of *P. hysterophorus* showed significant effects against *Spodoptera littura* and *Spodoptera littorallis* with LC_{50} values as 7.96% and 8.14%, respectively.

Gas Chromatographic- Mass Spectrometric (GC-MS) analyses offered the existence of various phytochemicals in different parts of the plants. In a systematic chemical study Wang, Gan [60], reported that solvent fraction of *P. hysterophorus*, contained fourteen chemical compounds were plant phytosterols, stigmasterol, daucosterol, dtigmasterol-3-O-glucopyranoside, and Stigmasteryl-3β-arachidate. The other six isolated terpenoids were parthenin, oleanolic acid, oleanolic acid 28-O-β-D-glucopyranoside, and dichrocepholide D. Moreover, Białoń, Krzyśko-Łupicka [61] assessed the chemical composition of Lavender oil and found various types of terpenoids (monoterpene, monoterpenoids, sesquiterpene and sesquiterenoids) with valuable anti-bacterial as well as anti-fungal activities. Likewise, analytical aspects of

Satureja montana and tincture were evaluated by chromatographic techniques (HPTLC) which showed its richness in polyphenols, phenolic and flavonoids contents could indicates its valuable anti-microbial and antioxidant activities and also provides basis for further clinical studies [62]. However, for unknown compounds which are likely to be found in the extract/essential oil, it is simpler to start with the GC-MS, associated with (NIST) library will help to identify at least a part of compounds which are composing the extract and then it would be easier to separate and investigate the still unidentified compounds. Moreover, in a study, isolation and determination of phenolic compounds from fresh water algae *Cladofora glomerata* by using Clourimetric method, chromatographic and HPLC techniques and identified nine phenolic compounds along with phenolic acids and flavonoids [63].

Parthenin a chief component was found in profusion in leaf extracts of *P. hysterophorus* which was positively correlated with cytotoxicity but not with phytotoxicity. Bajwa, Weston [64] perceived two compounds like chlorogenic acid and ambrosin were positively correlated with germination inhibition however, among identified phytochemicals vatirenene was present in high concentration as 19.8% in root. In a study Koo, Thomas [65] reported the existence of vatirenene in breath test which may be the possible cause of aspergillosis. Moreover, caryophyllene oxide is considered as an effective fungicidal mediator [66], and creation of resistance in reproductive parts [67, 68]. Likewise, globulol displays strong antibacterial activity [69]. However, the chemical compounds assessed during our current investigations from flower's extract of *P. hysterophorus* responsible towards antioxidant and insecticidal activity.

Although different studies has been conducted on phytochemistery, pharmacology as well as other biological activities but, studies on antioxidant activity, insecticidal

activity against *B. brassicae* and chemical analysis of flower's extract is limited so for. The increasing interest for food, medical and agrochemical industries and to develop botanical based natural antioxidants led towards ne investigations. Thus, this research represents the first investigation on antioxidant and insecticidal activity of flower's extract of *P. hysterophorus* against *B. brassicae*.

Conclusions

Parthenium hysterophorus is a noxious weed but also possessed numerous beneficial aspects as medical and agrochemical industries. The flower's extract of *P. hysterophorus* identified significant antioxidant activities with appreciable amount of phenolic and flavonoid contents. Similarly, the extract displayed prominent insecticidal activity against *Brevicoryne brassicae* a notorious sucking pest worldwide. Toxicity results displayed that *P. hysterophorus* extracts showed LC_{50} values of 1446 and 750µgmL^{-1} at 72 and 96 h exposure, respectively showed its effectiveness against *B. brassicae* . Moreover, antioxidant activity of *P. hysterophorus* extracts displayed strong correlation versus phenols, flavonoids and extract yield. Chemical analysis revealed the existence of various chemical compounds accountable for biological activities. Therefore, extract of *P. hysterophorus* might be used as antioxidant and insecticidal purposes as an alternative to synthetic chemical agents. However, more detailed study is required on extraction and purification of bioactive compounds and their evaluation as biological perspectives.

Acknowledgements

The support and supervision provided by Professor Zumin Gu, Ji Mingshan and experimental guidelines of lab mates of Biopesticides Laboratory, Plant Protection College Shenyang is greatly acknowledged.

Conflicts of Interest: "The authors declare no conflict of interest."

Data Availability: All relevant data are within the manuscript

Funding: This research was supported by National Key Research & Development Program of China (Grant No. 0201300). However, the funders had no role in study design, data collection and analysis, decision to publish, or preparation of the manuscript. Moreover, No any support was received by any third party in conducting this research, analyzing the data or preparing the manuscript for submission

Author Contributions: Conceptualization, M.J. and Z.G.; methodology, M.A.; T.A.; A.J., S; A.S. and F.A.; software, X.B. and T.u.H; validation, Z.G. formal analysis, M.J., Y.L. and Z.G.; investigation, M.J. and Z.G; resources, Z.G.; data curation, M.A. Writing—original draft, M.A.; writing—review & editing, M.J. visualization, Z.G., supervision, Z.G. and M.J.; funding acquisition, Z.G.

All authors have read and agreed to the published version of the manuscript

References

1. Sukanya S, Sudisha J, Hariprasad P, Niranjana S, Prakash H, Fathima S. Antimicrobial activity of leaf extracts of Indian medicinal plants against clinical and phytopathogenic bacteria. Afr J. Biotechnol. 2009;8(23).

2. Adkins SW, Shabbir A, Dhileepan K. Parthenium weed: biology, ecology and management: CABI; 2018.

3. Adkins S, Shabbir A. Biology, ecology and management of the invasive parthenium weed (Parthenium hysterophorus L.). Pest Manage Sci. 2014;70(7):1023-9.

4. Ray P, Gour HN. Puja Ray and H.N. Gour (2012). Integrated Management of *Parthenium hysterophorus* L. (Asteraceae): A weed of worldwide significance. Ind J Mycol Pl Path. 2012;5:605–32.

5. Kapoor RT. Awareness related survey of an invasive alien weed, *Parthenium hysterophorus* L. in Gautam Budh Nagar district, Uttar Pradesh, India. J Agric Technol. 2012;8(3):1129-40.

6. Khan N, Bibi K, Ullah R. Distribution pattern and ecological determinants of an invasive plant *Parthenium hysterophorus* L., in Malakand division of Pakistan. J Mt Sci. 2020;17:1670-83.

7. Kushwaha VB, Maurya S. Biological utilities of Parthenium hysterophorus. J Appl Nat Sci. 2012;4(1):137-43.

8. Kaur M, Aggarwal NK, Kumar V, Dhiman R. Effects and management of Parthenium hysterophorus: A weed of global significance. Int Sch Res Notices. 2014;2014.

9. Javaid A, Jabeen T, Khan IH, Jabeen K, Akbar M. Herbicidal potential of *Alternaria citri* Ellis and Pierce metabolites against *Parthenium hysterophorus*. L. Allelopathy J. 2022;55(1):25-34.

10. Khan IH, Javaid A, Ahmad S. Metabolites of *Penicillium citrinum* as potent herbicides against parthenium weed. Pak J Weed Sci Res. 2021;28(1):77-85.

11. Raza S, Chaudhary A, Mumtaz M, Bashir S. Antioxidant, iron chelating, lipase inhibition activities and metabolite’s prediction of hyrdoethanolic leaf extract of Conocarpus erectus. Food Res. 2019;4:482-7.

12. Seufi AM, Mohammed HA, Moussa FI, El-Din AAS, El-Saadani M, Taha TH, et al. Protective Effects of the Aqueous Extract of Black Mulberry Leaves, Morus nigra, on Chlorpyrifos Toxicity in Male Albino Rats. Jordan J Biol Sci. 2019;12(4).

13. Kapoor RT. Farmers’perception about *Parthenium hysterophorus* l.-a case study of handia and phulpur tehsils of allahabad district, uttar pradesh. 2016.

14. Picman AK, Elliott R, Towers G. Cardiac-inhibiting properties of the sesquiterpene lactone, parthenin, in the migratory grasshopper, Melanoplus sanguinipes. Can J Zool. 1981;59(2):285-92.

15. Lakshmi C, Srinivas C. Parthenium: A wide angle view. Indian J Dermatol Venereol Leprol. 2007;73(5):296.

16. Batish DR, Singh H, Kohli R, Saxena D, Kaur S. Allelopathic effects of parthenin against two weedy species, Avena fatua and Bidens pilosa. Environ Exp Bot. 2002;47(2):149-55.

17. Batish D, Singh H, Saxena D, Kohli R. Weed suppressing ability of parthenin-a sesquiterpene lactone from Parthenium hysterophorus. N Z Plant Prot. 2002;55:218-21.

18. Patel VS, Chitra V, Prasanna PL, Krishnaraju V. Hypoglycemic effect of aqueous extract of Parthenium hysterophorus L. in normal and alloxan induced diabetic rats. Indian J Pharmacol. 2008;40(4):183.

19. Selmi S, Rtibi K, Grami D, Sebai H, Marzouki L. Rosemary (Rosmarinus officinalis) essential oil components exhibit anti-hyperglycemic, anti-hyperlipidemic and antioxidant effects in experimental diabetes. Pathophysiology. 2017;24(4):297-303.

20. Asghari B, Zengin G, Bahadori MB, Abbas-Mohammadi M, Dinparast L. Amylase, glucosidase, tyrosinase, and cholinesterases inhibitory, antioxidant effects, and GC-MS analysis of wild mint (Mentha longifolia var. calliantha) essential oil: A natural remedy. Eur J Integr Med. 2018;22:44-9.

21. Borah A, Paw M, Gogoi R, Loying R, Sarma N, Munda S, et al. Chemical composition, antioxidant, anti-inflammatory, anti-microbial and in-vitro cytotoxic

efficacy of essential oil of Curcuma caesia Roxb. leaves: An endangered medicinal plant of North East India. Ind Crops Prod. 2019;129:448-54.

22. Manssouri M, Znini M, Majidi L. Studies on the antioxidant activity of essential oil and various extracts of Ammodaucus leucotrichus Coss. & Dur. Fruits from Morocco. J Taibah Uni Sci. 2020;14(1):124-30.

23. Zhang C, Zhao J, Famous E, Pan S, Peng X, Tian J. Antioxidant, hepatoprotective and antifungal activities of black pepper (Piper nigrum L.) essential oil. Food Chem. 2021;346:128845.

24. Dwivedi S, Dwivedi A, Dwivedi S. Folk lore uses of some plants by the tribes of Madhya Pradesh with special reference to their conservation. Ethnobot leafl. 2008;2008(1):105.

25. Shabbir A, Javaid A. Effect of aqueous extracts of alien weed Parthenium hysterophorus and two native asteraceous species on germination and growth of mungbean, Vigna radiata L. wilczek. J Agric Res. 2010;48(4):483-8.

26. Towers G, Subba Rao P, editors. Impact of the pan-tropical weed, Parthenium hysterophorus L. on human affairs. Proceedings of the first international weed control congress; 1992: Weed Science Society of Victoria Melbourne.

27. Espinosa-Rivero J, Rendón-Huerta E, Romero I. Inhibition of Helicobacter pylori growth and its colonization factors by Parthenium hysterophorus extracts. J Ethnopharmacol. 2015;174:253-60.

28. Tanvir R, Sajid I, Hasnain S. Screening of endophytic Streptomycetes isolated from Parthenium hysterophorus L. against nosocomial pathogens. Pak J Pharm Sci. 2013;26(2):277-83.

29. Deka B, Jiten M, Bhattacharyya P. Acaricidal activities of Parthenium hysterophorus L. against red spider mite, Oligonychus coffeae Nietner (Acarina: Tetranychidae) of tea. Int J Sci Res. 2013;4(5):2319-7064.

30. Krishnaveni M. Air pollution tolerance index and antioxidant activity of Parthenium hysterophorus. J Pharm Res. 2013;7(4):296-8.

31. Nabi SA, Kasetti RB, Sirasanagandla S, Tilak TK, Kumar MVJ, Rao CA. Antidiabetic and antihyperlipidemic activity of Piper longum root aqueous extract in STZ induced diabetic rats. BMC Complement Altern Med. 2013;13(1):37.

32. Panwar R, Sharma AK, Dutt D, Pruthi V. Phenolic acids from Parthenium hysterophorus: evaluation of bioconversion potential as free radical scavengers and anticancer agents. Advances in Bioscience and Biotechnology. 2015;6(01):11.

33. Anup R, Sainath P, Padma S, Sukumaran M. In vitro cytotoxic activity of methanolic extract of Parthenium hysterophorus flowers on MCF-7 and HeLa cell lines. Int J Curr Microbiol Appl Sci. 2015;4(8):710-5.

34. Kumar S, Nair G, Singh AP, Batra S, Wahab N, Warikoo R. Evaluation of the larvicidal efficiency of stem, roots and leaves of the weed, Parthenium hysterophorus (Family: Asteraceae) against Aedes aegypti L. Asian Pac J Trop Dis. 2012;2(5):395-400.

35. Kumar VP, Chauhan NS, Padh H, Rajani M. Search for antibacterial and antifungal agents from selected Indian medicinal plants. J Ethnopharmacol. 2006;107(2):182-8.

36. Jha U, Chhajed PJ, Oswal RJ, Shelke TT. Skeletal muscle relaxant activity of methanolic extract of Parthenium hysterophorus L. leaves in swiss albino mice. Int J Pharm Life Sci. 2011;2(11).

37. Datta S, Saxena DB. Pesticidal properties of parthenin (from Parthenium hysterophorus) and related compounds. Pest Manag Sci. 2001;57(1):95-101.

38. Bharadwaja S, Singh S, Moholkar VS. Design and optimization of a sono-hybrid process for bioethanol production from Parthenium hysterophorus. J Taiwan Inst Chem Eng. 2015;51:71-8.

39. Parashar V, Parashar R, Sharma B, Pandey AC. Parthenium leaf extract mediated synthesis of silver nanoparticles: a novel approach towards weed utilization. Dig J Nanomater Biostructures. 2009;4(1).

40. Rajiv P, Rajeshwari S, Venckatesh R. Bio-Fabrication of zinc oxide nanoparticles using leaf extract of Parthenium hysterophorus L. and its size-dependent antifungal activity against plant fungal pathogens. Spectrochimica Acta Part A: Mol Biomol Spectrosc. 2013;112:384-7.

41. Yang J, Jiang F, Ma C, Rui Y, Rui M, Adeel M, et al. Alteration of crop yield and quality of wheat upon exposure to silver nanoparticles in a life cycle study. J Agric Food Chem. 2018;66(11):2589-97.

42. Ahmad MA, Yuesuo Y, Ao Q, Adeel M, Hui ZY, Javed R. Appraisal of comparative therapeutic potential of undoped and nitrogen-doped titanium dioxide nanoparticles. Molecules. 2019;24(21):3916.

43. Javed R, Ahmad MA, Gul A, Ahsan T, Cheema M. Comparison of chemically and biologically synthesized nanoparticles for the production of secondary metabolites, and growth and development of plants. Compr Anal Chem. 2021;94:303-29.

44. Ahmed M, Ji M, Qin P, Gu Z, Liu Y, Sikandar A, et al. Determination of phytochemicals, antioxidant activity and biochemical composition of chinese

mugwort (*Artemisia argyi* l.) leaf extract from northeast China. Appl Ecol Environ Res. 2019;17(6):15349-62.

45. Sparkman OD. Identification of essential oil components by gas chromatography/quadrupole mass spectroscopy Robert P. Adams. J Am Soc Mass Spectrom. 2005;16(11):1902.

46. McConnachie A, Strathie L, Mersie W, Gebrehiwot L, Zewdie K, Abdurehim A, et al. Current and potential geographical distribution of the invasive plant Parthenium hysterophorus (Asteraceae) in eastern and southern Africa. Weed Res. 2011;51(1):71-84.

47. Narasimhan T, Murthy BK, Harindranath N, Rao PS. Characterization of a toxin fromParthenium hysterophorus and its mode of excretion in animals. J Bios. 1984;6(5):729-38.

48. Hasan N, Jain R. Biotoxicity of Parthenium hysterophorus extracts against Meloidogyne javanica and Helicotylenchus dihystera. Nematol Mediterr. 1984.

49. Kalaiselvi M, Subbaiya R, Selvam M. Synthesis and characterization of silver nanoparticles from leaf extract of Parthenium hysterophorus and its anti-bacterial and antioxidant activity. Int J Curr Microbiol Appl Sci. 2013;2(6):220-7.

50. Arshad M, Mumtaz MW, Chaudhary AR, Rashid U, Ali M, Mukhtar H, et al. Metabolite profiling of Cycas revoluta leaf extract and docking studies on alpha-glucosidase inhibitory molecular targets by phytochemicals. PakJ Pharm Sci. 2019;32(2 (Supplementary)):871-4.

51. Sinha SN, Paul D. Antioxidant potentials of parthenium hysterophorus L. leaf extracts. Sci Res J India. 2014;3(2):80-6.

52. Bashir S, Raza, S.A., Ahmad, M., Farhan, M., Ali, S. and William, J. Hydroethanolic leaf extract of Parthenium hysterophorus attenuates blood glucose in alloxan induced diabetic mice. Food Res 2021;5(1):72-7.

53. Ahmad J, Bagheri R, Bashir H, Baig MA, Al-Huqail A, Ibrahim MM, et al. Organ-specific phytochemical profiling and antioxidant analysis of Parthenium hysterophorus L. BioMed Res Int. 2018;2018.

54. Adeel M, Zain M, Fahad S, Rizwan M, Ameen A, Yi H, et al. Natural and synthetic estrogens in leafy vegetable and their risk associated to human health. Environ Sci Pollut Res. 2018;25(36):36712-23.

55. Rajiv P, Rajeshwari S, Yadav RH, Rajendran V. Vermiremediation: Detoxification of Parthenin toxin from Parthenium weeds. J Hazard Mater. 2013;262:489-95.

56. Tesfu F, Emana G. Evaluation of Parthenium hysterophorus L. powder against Callosobruchus chinensis L.(Coleoptera: Bruchidae) on chickpea under laboratory conditions. Afri J Agri Res. 2013;8(44):5405-10.

57. Shaheen FA, Khaliq A. Management of Pulse Beetle, Callosobruchus chinensis L.(Coleoptera: Bruchidae) in stored Chickpea using ashes, red soil powder and turpentine oil. Pak Entomol. 2005;27(2):19-24.

58. Yadav SK, Patel S. Insecticidal and repellent activity of some plant extracts against Myzus persicae (Sulzer) and Brevicoryne brassicae(Linnaeus). J Entomol Zool Stud. 2017;5(2):1434-143.

59. Wasu Yogesh H GD, Patel Sagar B. Larvicidal effects of *Parthenium hysterophorus* against polyphagus pests *Spodoptera Litura* and *Spodoptera littoralis*. Indian J Appl Res. 2019;9(2):1-19.

60. Wang M-H, Gan J-J, Chen W-H, Guan Y-L. Chemical Constituents of Parthenium hysterophorus. Chem Nat Comp. 2020;56:556-8.

61. Białoń M, Krzyśko-Łupicka T, Nowakowska-Bogdan E, Wieczorek PP. Chemical composition of two different lavender essential oils and their effect on facial skin microbiota. Molecules. 2019;24(18):3270.

62. Hudz N, Makowicz E, Shanaida M, Białoń M, Jasicka-Misiak I, Yezerska O, et al. Phytochemical Evaluation of Tinctures and Essential Oil Obtained from Satureja montana Herb. Molecules. 2020;25(20):4763.

63. Korzeniowska K, Łęska B, Wieczorek PP. Isolation and determination of phenolic compounds from freshwater Cladophora glomerata. Algal Res. 2020;48:101912.

64. Bajwa AA, Weston PA, Gurusinghe S, Latif S, Adkins SW, Weston LA. Toxic Potential and Metabolic Profiling of Two Australian Biotypes of the Invasive Plant Parthenium Weed (Parthenium hysterophorus L.). Toxins. 2020;12(7):447.

65. Koo S, Thomas HR, Daniels SD, Lynch RC, Fortier SM, Shea MM, et al. A breath fungal secondary metabolite signature to diagnose invasive aspergillosis. Clin Infect Dis. 2014;59(12):1733-40.

66. Yang D, Michel L, Chaumont J-P, Millet-Clerc J. Use of caryophyllene oxide as an antifungal agent in an in vitro experimental model of onychomycosis. Mycopathologia. 2000;148(2):79-82.

67. Saifullah K. Callus induction and cell suspension culture production of Catharanthus roseus for biotransformation studies of (-)-caryophyllene oxide. Pak J Bot. 2011;43(1):467-73.

68. Halim SA, Uddin R, Madura JD. Benchmarking docking and scoring protocol for the identification of potential acetylcholinesterase inhibitors. J Mol Graph Model. 2010;28(8):870-82.

69. Mulyaningsih S, Sporer F, Reichling J, Wink M. Antibacterial activity of essential oils from Eucalyptus and of selected components against multidrug-resistant bacterial pathogens. Pharm Biol. 2011;49(9):893-9.

Tables

Table 1. Physical appearance of the flower's extract of *Parthenium hysterophorus*.

Extract	Visibility	Physical Nature	Consistency
EtOH	Bluish green	Oily	Consistent
EA	Greenish	Oily	Consistent
DCM	Brownish	Oily/Gummy	Non-consistent
DW	Brownish	Oily/Gummy	Non-consistent

EtOH; (Ethanol), EA; (Ethyl Acetate), DCM; (Dichloromethane), DW; (Distilled water).

Table 2. Total Phenol, Flavonoid content and DPPH radical scavenging activity of the flower's extract of *Parthenium hysterophorus*.

Extracts	Phenol (GAE/g)	Flavonoid (QE/g)	DPPH Inhibition (%)
EtOH	27.55 ± 1.46^{c}	14.23 ± 0.10^{b}	88.53 ± 0.11^{b}
EA	47.79 ± 0.85^{b}	138.67 ± 2.20^{a}	67.21 ± 0.19^{c}
DCM	30.50 ± 0.00^{d}	15.38 ± 0.04^{c}	95.03 ± 1.58^{a}
DW	103.30 ± 1.16^{a}	4.81 ± 0.01^{c}	18.73 ± 0.89^{d}
Statics	S.S= 24268	S.S= 248063	S.S= 13880
	M.S=8069	M.S= 82687	M.S= 4626
	Df= 3	Df= 3	M.S= 3
	f= 2564***	*f*= 22488***	*f*= 1031***

Data in the columns is described as mean values ± standard deviations with various superscripts are significantly different according to DMRT" $P > 0.05$). S.S (Sum of square); M.S (Mean square); Df (Degree of freedom); *f* (Significance); *** (significance level).

Table 3. Correlation of the antioxidant activity of *P. hysterophorus* extract versus Phenols, Flavonoids and extract yield.

Treatments	Phenols	Flavonoids	DPPH	Yield
Phenols	1	-0.16	-0.99**	-0.11
Flavonoids	-0.16	1	-0.71	-0.90
DPPH	-0.99**	-0.71	1	-0.10
Yield	-0.11	-0.09	-0.10	1

Note: * Correlation is significant at 0.05 levels; ** Correlation is highly significant at 0.05 levels

Table 4. **Toxicity of *P. hysterophorus* extracts against *B. brassicae* at 12, 24, 48, 72 and 96 hours exposure**

Time	LC_{50} µgmL^{-1}	95% F.L		Slope± S.E	χ2
		Lower	Upper		
12	3598	1421	35512	1.09±0.38	0.25
24	2569	1321	27758	1.51±0.46	0.99
48	2345	1153	25517	1.31±0.53	0.85
72	1446	852	21721	1.56±0.63	0.22
96	750	468	1276	1.89±0.58	0.04

Note: LC_{50} (Lethal concentration); S.E. (standard error); χ2 (chi-square); F.L. (Fiducial limit)

Table 5. Chemical composition of the flower's extract of *Parthenium hysterophorus*.

Peak #	R. T	Area %	Chemical Compounds	M.F	M.W g/mol
1	3.22	7.05	Benzene, 1,3-dimethyl-	C_8H_{10}	106.17
2	3.92	3.47	2,4-Dimethylamphetamine	$C_{11}H_{17}N$	163.26
3	4.43	2.04	3-Piperidinol	$C_5H_{11}NO$	101.15
4	5.05	2.74	2-Pentene, 4,4-dimethyl-, (E)-	C_7H_{14}	98.186
5	5.52	3.55	2-Butanamine	C_6H_5N	101.19
6	12.96	25.93	2-Naphthalenemethanol,decahydro.alpha.,.α.,4a-trimethyl-8-methylene-,[2R (2 α.,4a.α.,8a.beta.)]-	$C_{15}H_{26}O$	222.36
7	14.29	11.27	3-Methoxy-3,4-dimethyl-1-heptyne	$C_{10}H_{18}O$	154.25
8	15.21	4.76	Undecane	$C_{11}H_{24}$	156.31
9	15.85	3.82	2-Pyrrolidinone, 1-methyl-	C_5H_9NO	99.13
10	16.62	7.26	Glycinamide hydrochloride	$C_2H_7C_lN_2O$	110.54
11	17.58	2.18	1,2-Benzenedicarboxylic acid, butyl 2-ethylhexyl ester	$C_{20}H_{30}O_4$	334.45
12	18.1	6.67	Hexadecanoic acid, ethyl ester	$C_{18}H_{36}O_2$	284.48
13	21.85	19.25	Histidine, 1,N-dimethyl-4-nitro-	$C_8H_{10}N_2O_2$	166.18

R.T (Retention time); M.F (Molecular formula); M.W (Molecular Weight).

Manuscript

Click here to access/download;Manuscript;Final version 26.05.2023.docx

Original Manuscript

Antioxidant, Insecticidal Activity and Chemical Profiling of Flower's Extract of Parthenium weed (*Parthenium hysterophorus* L.)

Maqsood Ahmed[1,2], Ansar Javeed[3], Samiullah[2], Talfoor-ul-Hassan[4], Aatika Sikandar[1], Taswar Ahsan[5], Faqir Ahmed[6], Ji Mingshan[1], Xuejing Bai[7], Ying Li[8] and Zumin Gu[1,*]

[1]College of Plant Protection, Shenyang Agricultural University, Shenyang, 110866, P.R. China. aatika_sikander@yahoo.com (A.S.); jimingshan@syau.edu.cn (M.J.)
[2]Department of Agriculture (Plant Protection), Pest Warning and Quality Control of Pesticides, Gujrat, 50700, Pakistan. maqsoodahmed200@hotmail.com
[3]Henan University, Jinming Campus, Kaifeng City Henan China. ansarjaveed@henu.edu.cn (A.J)
[4]Department of Agriculture (Plant Protection), Pest Warning and Quality Control of Pesticides, Gujranwala, 52250, Pakistan. ddappgujranwala@gmail.com (T.u.H.)
[5]Institute of Plant Protection Liaoning Academy of Agricultural Sciences, Shenyang, 110866, P.R. China. taswarahsan@163.com (T.H)
[6]Directorate General of Agriculture, Pest Warning and Quality Control of Pesticides, Lahore, 54800, Pakistan. faqirahmad63@yahoo.com (F.A)
[7]Shenyang Academy of Landscape- Gardening Shenyang 110016, P.R. China. baixuejingnv@163.com (X.B.)
[8] Fushun Modern Agriculture and Poverty Alleviation Development Promotion Centre, Fushun 113006 P.R. China. liying_1973@163.com (Y.L.)

Corresponding author: *guzumin1212@163.com *(G.Z); Tel: +86-24- 8848- 7148; Fax: +86-24-8834-2315*

Abstract

Parthenium hysterophorus L., an invasive alien species and notorious weed, offers various benefits to the medical and agrochemical industries. This study aimed to evaluate the antioxidant and insecticidal activities of *P. hysterophorus* flower extract and conduct chemical profiling to identify the phytoconstituents responsible for these biological effects. The antioxidant activity was assessed using the 1,1-diphenyl-2-picrylhydrazyl (DPPH) assay, while gas chromatography mass spectrometry (GCMS) analysis was employed for chemical configuration evaluation. Our findings demonstrate that the dichloromethane (DCM) extract of *P. hysterophorus* exhibits potent radical scavenging activity (95.03%). Additionally, phytochemical analysis revealed significant amounts of phenols and flavonoids in the distilled water and ethyl acetate extracts (103.30 GAEg-1 and 138.67 QE-g-1, respectively). In terms of insecticidal activity, the flower extract displayed maximum mortality rates of 63.33% and 46.67% after 96 hours of exposure at concentrations of 1000 µg/mL and 800 µg/mL, respectively, with similar trends observed at 72 hours. Furthermore, the *P. hysterophorus* extracts exhibited LC50 values of 1446 µg/mL at 72 hours and 750 µg/mL at 96 hours. Imidacloprid, the positive control, demonstrated higher mortality rates at 96 hours (97.67%) and 72 hours (91.82%). Moreover, the antioxidant activity of *P. hysterophorus* extracts exhibited a strong correlation with phenols, flavonoids, and extract yield. GCMS analysis identified 13 chemical compounds, accounting for 99.99% of the whole extract. Ethanol extraction yielded the highest percentage of extract (4.34%), followed by distilled water (3.22%), ethyl acetate (3.17%), and dichloromethane (2.39%). The flower extract of *P. hysterophorus* demonstrated significant antioxidant and insecticidal activities, accompanied by the presence of valuable chemical compounds responsible for these biological effects, making it a

promising alternative to synthetic agents. These findings provide a novel and fundamental basis for further exploration in purifying the chemical compounds for their biological activities.

Keywords: *Parthenium hysterophorus*; phytochemicals; Antioxidant activity; GCMS; DPPH

Introduction

The introduction of invasive alien species, such as Parthenium hysterophorus, has devastating consequences for native biota and ecosystems, contributing to climate change, pollution, and habitat destruction. Understanding the positive and negative impacts of this species can aid in controlling biological invasions, which pose a global risk to biodiversity and agricultural production. Invasive weeds not only compete with crops for resources but also produce bioactive compounds that hold potential for medicinal purposes, highlighting the importance of natural plants in pharmaceutical and herbal systems [1].

Parthenium hysterophorus L. is an extremely invasive plant species belonging to family Asteraceae that has invaded almost fifty countries in sub-continent Africa, Asia, and Oceania, infesting forests, feeding lands, and urban landscapes [2, 3]. It is a devastating and hazardous weed of various economically important crops [4]. Moreover, it is amongst the top ten worst weeds in the world and is included in the world catalogue of destructive species [5, 6]. In Europe *P. hysterophorus* is considered as an ephemeral species. It is generally recognized by different names such as parthenium weed, congress grass, Gajjar botti, white head and white top [7]. It is native to Gulf of Mexico and United States. However, it accidently introduced in Indian subcontinent [8]. This weed has been considered as a major weed of high rank spreading at a much faster pace to takeover abandoned land, canal, watercourse banks, roads, and fields to challenge crop productivity [9, 10].

Medicinal plants are the fundamentals of traditional therapeutic systems based on the trusts and observations comprised of hundreds of years [11]. The phytochemical constituents such as phenolic and flavonoid are secondary metabolites involved in therapeutic system, because of the existence of these functional phytochemicals

mainly parthenin [12]. Moreover, *P. hysterophorus* renowned to contain vital biochemical compounds such as alkaloids, tannins, and saponins, which are important from the medicinal viewpoint [13]. It also exhibited allelopathic effects and important phytochemicals such as parthenon and coronopilin which are auto-toxic to its own seed germination and seedling growth [14]. Furthermore, serious health consequences on human beings and livestock have been reported [15]. The agriculturists who are come in contact with *P. hysterophorus*; the pollen and dust produced by this weed provoke allergic contact dermatitis in human beings. Major chemical compounds, parthenin's contained by *P. hysterophorus* exhibited phytotoxic properties against a variety of plants, including weeds and various crops such as Slender amaranth (*Amaranthus viridis*), Billygoat weed (*Ageratum conyzoides*), common wild oat. (*Avena fatua*), nettleleaf goosefoot (*Chenopodium murale*), Foetid cassia (*Cassia tora*), mung bean *Phaseolus aureus* [16, 17]. Moreover, parthenin's the main biochemical compounds showed insecticidal activity against termites, cockroaches and migratory grasshoppers, *Melanoplus sanguinipes* [7]. Similarly, Patel, Chitra [18] reported pesticidal activity of the ethanol extract of *P. hysterophorus* which showed substantial lethal effect against stored grain pest like *Tribolium* sp. and *Oryzaephilus* sp. Essential oils from the natural plants also have antioxidant effects. In recent years several reports have been breakout, the essential oil from Rosemary [19], wild mint (*Mentha longifolia* var. *Calliantha*) [20], black turmeric (*Curcuma caesia*) [21] *Ammodaucus leucotrichus* [22] and black pepper (*Piper nigrum*) [23].

Pharmacologically, it is involved in treating numerous diseases like allergies, anemia, sores, skin diseases, facial neuralgia, fever, vermifuge, blood purifier, and insecticide [7, 24]. This plant species is enriched with vital phytoconstituents however; sesquiterpene lactones are the most copious phytochemicals exhibited by

this plant which are accountable for injurious effects such as strong allelopathic [25], toxic to livestock [26], It also possess many advantageous effects like antibacterial [27, 28], acaricidal [29], antioxidant [30, 31], cytotoxic [32, 33], larvicidal potential against *Aedes aegypti* L. [34, 35], skeletal muscle relaxant [36], pesticidal agent [37], producing bioethanol [38], as reducing agent in the formation of silver nanoparticles [39] and in production of zinc nanoparticles [40]. However, various factors affect the biological profile of the plants and from some recent studies it has been reported that nanoparticle influenced the physiological and biological properties of the plants. It has been reported that metal-based nanoparticle showed a decrease in protein and amino acid and micronutrient contents in wheat crop grown under such conditions [41]. Moreover, nitrogen doped titanium dioxide nanoparticle enhanced antioxidant activity via reducing power assay and percent inhibition of DPPH against *Streptomyces* [42]. Likewise, synthetically and biologically prepared nanoparticle significantly exerted effects on plant growth and secondary metabolites which ultimately triggered the biological activity of the plants [43].

As some earlier researches have been conducted on this plant species but, detailed studies on chemical and quantitative phytochemical analysis and assessment of antioxidant activities from different solvents extract as well as aphicidal activity against cabbage aphid *B. brassicae* from flower’s extracts has not been explored before. Hence, keeping in view the diverse biological properties of *P. hysterophorus*, current study was designed to determine the antioxidant and insecticidal activity of flower’s extract against *B. brassicae* with appraisal of quantitative phytochemical analysis as well as assessment of chemical profiling of extract.

Materials and Methods

Collection and Samples Preparation

The whole plant samples were collected from District Sialkot Punjab Pakistan during the month of June-July 2019 and were identified as *Parthenium hysterophorus* L., from Ayub Agricultural Research Institute (AARI) Faisalabad Pakistan by Dr. Dilbar Hussain. Flowers were manually separated from the plants and left over for 20 days to dry under shade. These dried flowers were powdered by electric grinder and then extracted with different solvents (ethanol, ethyl acetate, dichloromethane and distilled water) by cold extraction method for seventy two hours at fixed temperature 28°C and at 100 rpm in an incubator shaker (ZWY-1102C). Extracted contents were filtered and concentrated to lessen the volume on rotary evaporator model R-210 BUCHI Labortechnik AG, CH-9230 Flawil 1/Switzerland). Obtained extract's yield was measured using following Equation (Eq. 1).

$$\text{Yield (\%)} = \frac{\text{Weight of the extract}}{\text{Weight of the dried sample}} \times 100 \qquad \text{(Eq. 1)}$$

Quantitative Analysis for Total Phenolic and Flavonoids Content

To assess the phytochemicals quantitatively, 1 mg of extract from each solvent was dissolved in 1 mL Methanol separately. Consequent mixture was vortexed and separated for analysis. For the assessment of total content for phenols Folin–Ciocalteu reagent test was used. In brief, 1 mL solution (1 $mgmL^{-1}$) was added to 2.5 mL of (10%) Folin–Ciocalteu with supplementary addition of 2 mL solution of (2%) sodium carbonate (Na_2CO_3). The consequential blend was incubated for fifteen min in the dark to measure absorbance at 765 nm in 96-well ELISA plate using (SpectraMax 190, Meigu molecular International Co. Ltd., Shanghai China). To build a standard curve, Gallic acid was used (1 $mgmL^{-1}$) at different concentrations such as 1, 0.50, 0.25, 0.10, 0.05, 0.02, 0.01, 0 mg mL^{-1} to describe the obtained results as Gallic acid equivalent (GAE) mg g^{-1} of total extract. Each treatment was replicated ten times to analyze the data.

To examine the total flavonoids content, aluminium chloride colourimetric method was adopted. Briefly, 1 mL solution ($1mgmL^{-1}$) was poured into 3 mL methanol, with addition of 0.2 mL of 1 M Potassium acetate (CH_3COOK), 0.2 mL of (10%) aluminium chloride ($AlCl_3$) and at the end, distilled water 5.6 mL was added to the consequent mixture. The incubation of solution was performed for half an hour in the darkness and absorbance at 420 nm was measured. A standard curve was built using quercetin at different concentrations such as 1, 0.50, 0.25, 0.10, 0.05, 0.02, 0.01, 0 $mgmL^{-1}$ to represent the results as quercetin equivalent (QE) mg g^{-1} of total extract. The procedure was replicated 10 times for each treatment.

Assessment of Free Radical Scavenging Activity

Antioxidant activity of extracts obtained by different solvents from *Parthenium hysterophorus* flowers was accomplished in 1% solution of tween 20 by using 1,1-diphenyl-2-picrylhydrazyl (DPPH) ($C_{18}H_{13}N_5O_6$) a free radical. In brief, to assess the free radical scavenging activity, all dried solvent extracts were mixed together in equal proportion (5 mg each) and were dissolved in HPLC grade methanol and filtered. The filtrate was formerly used to assess the antioxidant activity. Similarly, DPPH solution was prepared in same grade Methanol @ 0.001g 25 mL^{-1}. Then, into 3.5 mL freshly prepared DPPH solution and 0.25 mL extract prepared in Methanol was added. The consequent mixture was shacked for complete mixing and then the resulting mixture was incubated in darkness at 28°C for 30 min [44]. After incubation of resulting mixture for stipulated period of time, the absorbance was assessed at 517nm by using (SpectraMax 190, Meigu molecular International Co. Ltd., Shanghai China). The inhibition percentage of primed DPPH solution was predicted on reduction of absorbance through following Equation (Eq. 2). Each treatment was replicated ten times to analyze the data.

$$\text{Inhibition (\%)} = \frac{A_{\text{blank}} - A_{\text{sample}}}{A_{\text{blank}}} \times 100 \qquad \text{(Eq. 2)}$$

Where: A_{blank} = (control absorbance); A_{Sample} = (samples absorbance).

Correlation of Antioxidant Activity versus Phenols, Flavonoids and extract yield

Correlation of antioxidant activity *P. hysterophorus* versus Phenols, Flavonoids and extract yield was carried out using SPSS statistics 25.0 version with significant values at ($P>0.05$) level.

Assessment of Insecticidal Activity

Residual toxicity method was adopted for the assessment of insecticidal activity of extract of *P. hysterophorus* flowers. Samples for bioassay study were prepared to obtain mother mixture by mixing 0.1g of each solvent's extract and dissolved in Acetone, because it is aprotic solvent having intermediate polarity comparable to polar parotic solvents and it also evaporates immediately from the extract when exposed to air. The resulting mixture was placed in the fume hood for six hours for complete drying of Acetone. Serial concentrations *viz*. 100, 200, 400, 800 and 1000 µg mL^{-1} was prepared in Tween-20 (1%) solution from mother mixture. Briefly, fresh cabbage leaf discs 5cm in diameter were cut off and dipped for 10 s in the respective concentration and then dried for 5 min at room temperature. Formerly, 10 adult wingless aphids were carefully released onto the leaf disc using a fine-haired brush contained in the Petri dish and incubated for 96 h at 65% R.H., 25°C and with a 16:8 (light: dark) photoperiod. Imidacloprid 25% WP was used as the positive control at a rate of 0.0025 mL mL^{-1} of water, and control (CK) with a 1% Tween 20 solution. Positive controls and CKs were placed in a greenhouse for 96 h. Mortality data was collected at 12, 24, 48, 72 and 96 h using binocular microscope and the response of aphids was observed by needle probing, and the aphids who offered no response on stimulation were considered dead. Each treatment was replicated five times.

Chemical Analysis

Samples for analysis were prepared by mixing 0.1 g of each solvent extract into 2 mL of HPLC grade methanol to obtain mother solution. Consequent mixture was vortexed for 1 min following the addition of graphitized carbon black “GCB” (0.005 g). Mixture was centrifuged for 10 min at 10000 rpm at 25°C and colorless material was separated and collected in (1.5 mL) centrifuges tubes. Work for GC-MS analysis was accomplished using an Agilent Model 6890-5973N accompanied with gas chromatograph established on HP1 capillary column TG-5MS polydimethylsiloxane (length 30m × diameter 250 μm × film thickness 0.25 μm) interfaced with mass selective detector Hewlett Packard (5973N). The established parameters were; preliminary temperature was adjusted at 70°C for 0 min however, final temperature was extended to 200 °C for 10°C min^{-1} whereas, inlet temperature was adjusted at 250°C with split ratio 10:1. MS quadruple temperatures were fixed at 150°C while thermal aux temperatures were 285°C. The Scan range for MS was 35–520 units however; as a carrier, helium gas was used bearing stream frequency of 1.0mLmin^{-1}. The existing compounds in the extract were identified by comparison of their retention time and the mass spectra of the NIST 08 and Wiley 7 of the data base and confirmed by comparing with GC-MS literature data at NIST and TMS database Wiley/NIST.1998.1 [45].

Statistical Analysis

All calculated data was analysed by one way analysis of variance (ANOVA), mean difference between treatments was calculated for significance test by Duncan Multiple Range Test “DMRT ” at $P > 0.05$) with IBM-SPSS statistics 25.0 version software. Probit analysis was performed using EPA Probit analysis program version 1.5.

Results

The current study carried out on the flower's extract of *Parthenium hysterophorus* revealed the existence of medicinally and agro-chemically active compounds. The phytochemical constituents of *P. hysterophorus* were qualitatively analyzed for antioxidant and insecticidal activities.

Extract Yield (%)

Yield of extract by the solvent extraction technique from *P. hysterophorus* flowers was calculated and presented in (Figure 1). The maximum extract was obtained by ethanol (4.34%) whereas; minimum extract quantity (2.39%) was afforded by dichloromethane. The extract obtained via ethanol and ethyl acetate was bluish green and greenish in color and exhibited consistency, respectively. The same oil was brownish in color and non-consistent via dichloromethane and distilled water, respectively, (Table 1).

Analysis for Phenol and Flavonoid and Antioxidant Activity

The total contents for phenol and flavonoid as well as DPPH scavenging activity afforded by *P. hysterophorus* flower's extract via different solvents are presented in (Table 2). Results confirmed that the highest total phenol were reported from distilled water 103.30 GAE/g followed by Ethyl acetate and Ethanol' extract, 47.79 and 27.55 GAE/g, respectively. On the other hand highest flavonoid was reported from Ethyl acetate extract 138.67 QE/g followed by Ethanol 14.23 QE/g respectively.

Inhibition (%) was determined by 1,1-Diphenyl-1-picrylhydrazyl (DPPH) displayed color absorption at 517nm on using spectrophotometer. When DPPH trap free radicals, the color of the solution changed to light yellow and, subsequently, resulted in decrease of the absorbance. Maximum DPPH radical scavenging activity was afforded by Dichloromethane extract 95.03% followed by Ethanol and Ethyl acetate 88.53% and 67.21%, respectively.

Correlation of Antioxidant Activity versus Phenols, Flavonoids and extract yield

In our study, the Pearson's correlation regarding antioxidant activity of *P. hysterophorus* showed positive relationship (Table 3). Results showed highly significant (P<0.01) negative effect of phenol with DPPH as well as DPPH showed strongly highly significant negatively correlation and similar results were found reciprocally in the studied parameters. However, yield extracted by different solvents did not affect the Antioxidant Activity versus phenols, flavonoids quantitatively.

Assessment of Insecticidal Activity

The mortality data of cabbage aphid *B. brassicae* at specific period of time using *P. hysterophorus* flower's extract is presented in (Figure 2). It was perceived that the percent mortality was directly correlated to the concentration and the time. The results demonstrated that the maximum mortality was recorded at 1000 µgmL^{-1} at 96 h and 72 h with 63.33% and 46.67%, respectively. Same trend of mortality was also observed at 800 µgmL^{-1} with 56.67% and 36.67%, respectively. Imidacloprid 25% WP being a positive control produced the highest rates of mortality after 96 h of exposure 97.67%. Likewise, Imidacloprid also produced significant mortality after 72 h and 48 h exposure 91.82% and 87.38% mortality, respectively.

Analysis for probability of the data showed the LC_{50} values, slope, chi-square, and fiducial limits at confidence interval of 95% for *P. hysterophorus* extracts. Mortality response using plant extracts showed sensitivity of *B. brassicae* to various concentrations which is represented in (Table 4).

Chemical Analysis

The existence of chemical compounds in *P. hysterophorus* flower's extract was analyzed by GCMS techniques and data is presented in (Table 5). Thirteen

biochemical compounds equivalent to 99.99% of entire extract by GC fraction were identified. Moreover,2-naphthalenemethanol,decahydro-.alpha.,.alpha.,4a-trimethyl-8-methylene-, [2R--(2.alpha., 4a.alpha.,8a.beta.)]-(25.93%), histidine, 1,N-dimethyl-4-nitro- (19.25%) and 3-methoxy-3, 4-dimethyl-1-heptyne (11.27%), were the major chemical compounds while, 10 others chemical compounds were identified in minor amounts having areas with peak range 2.04–3.7%. GC-MS chromatogram of *P. hysterophorus* flower extract is presented in (Figure 3).

Discussion

Parthenium hysterophorus, known as Parthenium weed, is recognized as a noxious weed with detrimental effects on ecosystems. However, our study revealed that this invasive plant species possesses valuable properties in terms of antioxidant and insecticidal activities.

The chemical compounds found in *P. hysterophorus*, such as phenolics, steroids, alkaloids, flavonoids, and terpenoids, vary in concentration based on ecological factors and pathogen presence. These compounds exhibit bio-pesticidal properties, specifically targeting insect pests and pathogens. Importantly, they are biodegradable and do not pose significant harm to the environment [27, 46-48].

In addition to its antioxidant activity, the flower extract of *P. hysterophorus* exhibited promising insecticidal activity against *Brevicoryne brassicae*, a notorious sucking pest. This finding suggests that the extract has the potential to be used as a natural insecticide. The observed LC50 values indicate the concentration at which the extract is lethal to 50% of the exposed insects. The lower LC50 value at 96 hours compared to 72 hours indicates the increased effectiveness of the extract over time. These results are consistent with previous studies that have reported the insecticidal properties of plant extracts against various pests [49]. The assessment of antioxidant and

polyphenolic activity in medicinal products is crucial for understanding the therapeutic potential of natural plants. Kumar and Mishra [44] identified alkaloids, flavonoids, terpenoids, and cardiac glycosides in the extract of *P. hysterophorus*, while the flower extracts exhibited phenolic contents ranging from 86.69-320.17 mg propyl gallate equivalent (PGE)/g. These findings align with our study, highlighting the significant antioxidant activity of the bioactive compounds present in *P. hysterophorus* extracts. Previous research on *P. hysterophorus* leaves extract reported a 50% radical scavenging potential at 250 µg/mL in the DPPH assay and 30% inhibition at 100 µg/mL in hydrogen peroxide assays [50], indicating the antioxidant potential of the plant extract. This antioxidant activity in the hydro-ethanolic extracts of *P. hysterophorus* leaves is likely attributed to the presence of polyphenolic compounds [11, 51]. The flower extract of *P. hysterophorus* exhibited significant antioxidant activity, as evidenced by its potent radical scavenging ability. This finding is in line with previous studies that have reported the antioxidant potential of various plant extracts [52]. The presence of phenolic and flavonoid compounds in the extract may contribute to its antioxidant activity. Phenols and flavonoids are well-known antioxidant compounds that can effectively neutralize free radicals and protect against oxidative stress [53]. The strong correlation observed between antioxidant activity, phenolic/flavonoid content, and extract yield further supports the potential of *P. hysterophorus* as a source of natural antioxidants.

Our findings on the antioxidant activity of *P. hysterophorus* are consistent with previous studies. Sinha and Paul [54] reported similar scavenging properties (54.5%) and IC50 values (60.2) via the DPPH assay, as well as high scavenging activity (93.2%) and IC50 values (33.6) via the ABTS assay. Likewise, Bashir [55] demonstrated the presence of significant phenolic and flavonoid contents (105.44 mg

GAE/g and 41.50 mg RE/g, respectively) in a 60% ethanolic leaf extract of *P. hysterophorus*, along with comparable IC50 values for DPPH radical scavenging activity (87.55 µg/mL) and ABTS (98.22 µg/mL). Furthermore, the phytochemical profiling of *P. hysterophorus* revealed the presence of terpenes, fatty acids, hydrocarbons, and phytosterols, with the root extract exhibiting the highest antioxidant potential [56]. These findings align with Sinha and Paul [54], who reported substantial antioxidant activity in crude leaf extracts of *P. hysterophorus*, with IC50 values of 60.24% and 33.60% using the DPPH and ABTS methods, respectively..

Previous research has demonstrated that exposure of natural fauna to harmful chemicals can have significant impacts on biological activities. For instance, estrogen compounds have been found to cause acute toxicity in edible crops and vegetables, particularly when long-term irrigation with estrogen-containing water is involved. Adeel, Zain [57] reported similar results, showing the uptake and accumulation of l7β-estradiol (17β-E2) and ethinylestradiol (EE2) in lettuce (Lactuca sativa) grown under laboratory conditions. These findings emphasize the potential risks associated with the contamination of agricultural systems by estrogen compounds.

The utilization of secondary metabolites, including terpenoids, alkaloids, and flavonoids, as pest control agents has gained attention due to historical practices involving nicotine and pyrethrum [58]. Our findings align with previous studies by Tesfu and Emana [59], who observed significant mortality rates of *Callosobruchus chinensis* after 96 hours of exposure to different parts of *P. hysterophorus* extract. Inflorescence and stem powder demonstrated mortality rates of 76.67% and 56.67%, respectively, while the leaves extract exhibited repellent effects of 70-100% on *Aedes aegypti* adults. Additionally, the diethyl ether extracts showed exceptional repellency

at 99.7%.The insecticidal effects of plant extracts can be attributed to their repellency and stomach poisoning properties, which hinder insect movement and cause death by constricting their spiracles [60]. Chemical analysis of *P. hysterophorus* revealed the presence of sesquiterpene lactones, particularly parthenin derivatives, which exhibited insecticidal and nematicidal activities against *Callosobruchus maculatus* adults and *Meloidogyne incognita* root knot nematodes. Notably, the pyrazoline compound demonstrated high efficacy as an insecticide, with an LC50 value of 32 mg/L after 72 hours of exposure, and also exhibited strong nematicidal activity, with an LC50 value of 512 mg/L for parthenin after 72 hours [37]. The chemical profiling of the *P. hysterophorus* flower extract using GCMS analysis revealed the presence of several chemical compounds, indicating its complex chemical composition. These compounds could be responsible for the observed biological activities, including antioxidant and insecticidal effects. Further investigation is warranted to isolate and purify these bioactive compounds and evaluate their specific biological properties. The identification and characterization of these compounds can contribute to the development of natural products for antioxidant and insecticidal purposes.

P. hysterophorus extract exhibited remarkable effectiveness against *Lipaphis erysimi*, reducing infestation by 29%, possibly due to the presence of phenolic acids [61]. Ahmad, Bagheri [56] reported mortality rates of 26.70% and 27% in mustard aphids treated with *P. hysterophorus* extract concentrations of 10% and 20% respectively, after 21 days. Additionally, Wasu Yogesh H [62] demonstrated high mortality (100% with freshly prepared solution, 80% with stored stock) of *Aedes aegypti* larvae when exposed to *P. hysterophorus* leaf and stem extract (200g/500 ml). Another study highlighted significant mortality rates of 73.33%, 86.67%, and 83.33% against *B. brassicae* and moderate mortality rates of 26.67%, 46.67%, and 63.33% against

Myzus persicae at 10% concentration and exposure times of 24, 48, and 72 hours respectively [58]. Furthermore, Wasu Yogesh H [62] reported promising effects of crude extracts from fresh *P. hysterophorus* leaves against *Spodoptera littura* and *Spodoptera littorallis*, with LC50 values of 7.96% and 8.14% respectively.

Gas chromatography-mass spectrometry (GC-MS) analysis revealed the presence of various phytochemicals in different parts of *P. hysterophorus*. Wang, Gan [63] identified fourteen chemical compounds, including phytosterols, stigmasterol, daucosterol, dtigmasterol-3-O-glucopyranoside, and Stigmasteryl-3β-arachidate. Similarly, Białoń, Krzyśko-Łupicka [64] found diverse terpenoids in Lavender oil, exhibiting antibacterial and antifungal properties. The chemical composition of *Satureja montana* and its tincture demonstrated high polyphenol, phenolic, and flavonoid contents, suggesting potential antimicrobial and antioxidant activities for further clinical investigation [65]. GC-MS coupled with the NIST library enabled the identification of known compounds in extracts, facilitating the separation and exploration of unidentified compounds. In another study, phenolic compounds from *Cladofora glomerata* algae were isolated and characterized using colorimetric, chromatographic, and HPLC methods, revealing the presence of nine phenolic compounds, phenolic acids, and flavonoids [66]. In the leaf extracts of *P. hysterophorus*, a significant abundance of parthenin, a key component, was observed, showing a positive correlation with cytotoxicity but not with phytotoxicity. Bajwa, Weston [67] identified chlorogenic acid and ambrosin as compounds positively correlated with germination inhibition, while vatirenene was found in high concentrations (19.8%) in the roots. Vatirenene was also detected in breath tests, reported by Koo, Thomas [68], potentially linked to aspergillosis. Additionally, caryophyllene oxide demonstrates effective fungicidal properties [69] and can induce resistance in reproductive parts [70, 71], while globulol

exhibits strong antibacterial activity [72]. Our current investigation highlights the flower extract of *P. hysterophorus* as a rich source of chemical compounds responsible for antioxidant and insecticidal activities.

While various studies have explored the phytochemistry, pharmacology, and other biological activities of *P. hysterophorus*, there is limited research on its antioxidant activity, insecticidal properties against *B. brassicae*, and chemical analysis of the flower extract. Given the growing interest in botanical-based natural antioxidants for the food, medical, and agrochemical industries, this study fills a critical research gap by investigating the antioxidant and insecticidal potential of *P. hysterophorus* flower extract against *B. brassicae*, providing valuable insights into its novel applications.

Conclusions

Parthenium hysterophorus, despite being a noxious weed, exhibits significant beneficial properties for the medical and agrochemical industries. The flower extract displays remarkable antioxidant and insecticidal activities, particularly against *Brevicoryne brassicae*, a widespread sucking pest. The extract's potency is further supported by its LC50 values of 1446µg/mL and 750µg/mL at 72 and 96 hours, respectively. The strong correlation between antioxidant activity, phenolic/flavonoid content, and extract yield highlights its potential. Chemical analysis reveals the presence of various bioactive compounds responsible for these activities. Therefore, *P. hysterophorus* extract shows promise as an alternative to synthetic chemicals for antioxidant and insecticidal purposes. However, further research is necessary to optimize extraction, purification, and evaluate the biological potential of these compounds.

Acknowledgements

The support and supervision provided by Professor Zumin Gu, Ji Mingshan and experimental guidelines of lab mates of Biopesticides Laboratory, Plant Protection College Shenyang is greatly acknowledged.

Conflicts of Interest: “The authors declare no conflict of interest.”

Data Availability: All relevant data are within the manuscript

Funding: This research was supported by National Key Research & Development Program of China (Grant No. 0201300). However, the funders had no role in study design, data collection and analysis, decision to publish, or preparation of the manuscript. Moreover, No any support was received by any third party in conducting this research, analyzing the data or preparing the manuscript for submission

Author Contributions: Conceptualization, M.J. and Z.G.; methodology, M.A.; T.A.; A.J., S; A.S. and F.A.; software, X.B. and T.u.H; validation, Z.G. formal analysis, M.J., Y.L. and Z.G.; investigation, M.J. and Z.G; resources, Z.G.; data curation, M.A. Writing—original draft, M.A.; writing—review & editing, M.J. visualization, Z.G., supervision, Z.G. and M.J.; funding acquisition, Z.G.

All authors have read and agreed to the published version of the manuscript

References

1. Sukanya S, Sudisha J, Hariprasad P, Niranjana S, Prakash H, Fathima S. Antimicrobial activity of leaf extracts of Indian medicinal plants against clinical and phytopathogenic bacteria. Afr J. Biotechnol. 2009;8(23).

2. Adkins SW, Shabbir A, Dhileepan K. Parthenium weed: biology, ecology and management: CABI; 2018.

3. Adkins S, Shabbir A. Biology, ecology and management of the invasive parthenium weed (*Parthenium hysterophorus* L.). Pest Manage Sci. 2014;70(7):1023-9.

4. Ray P, Gour HN. Puja Ray and H.N. Gour (2012). Integrated Management of *Parthenium hysterophorus* L. (Asteraceae): A weed of worldwide significance. Ind J Mycol Pl Path. 2012;5:605–32.

5. Kapoor RT. Awareness related survey of an invasive alien weed, *Parthenium hysterophorus* L. in Gautam Budh Nagar district, Uttar Pradesh, India. J Agric Technol. 2012;8(3):1129-40.

6. Khan N, Bibi K, Ullah R. Distribution pattern and ecological determinants of an invasive plant *Parthenium hysterophorus* L., in Malakand division of Pakistan. J Mt Sci. 2020;17:1670-83.

7. Kushwaha VB, Maurya S. Biological utilities of *Parthenium hysterophorus*. J Appl Nat Sci. 2012;4(1):137-43.

8. Kaur M, Aggarwal NK, Kumar V, Dhiman R. Effects and management of Parthenium hysterophorus: A weed of global significance. Int Sch Res Notices. 2014;2014.

9. Javaid A, Jabeen T, Khan IH, Jabeen K, Akbar M. Herbicidal potential of *Alternaria citri* Ellis and Pierce metabolites against *Parthenium hysterophorus*. L. Allelopathy J. 2022;55(1):25-34.

10. Khan IH, Javaid A, Ahmad S. Metabolites of *Penicillium citrinum* as potent herbicides against parthenium weed. Pak J Weed Sci Res. 2021;28(1):77-85.

11. Raza S, Chaudhary A, Mumtaz M, Bashir S. Antioxidant, iron chelating, lipase inhibition activities and metabolite's prediction of hyrdoethanolic leaf extract of Conocarpus erectus. Food Res. 2019;4:482-7.

12. Seufi AM, Mohammed HA, Moussa FI, El-Din AAS, El-Saadani M, Taha TH, et al. Protective Effects of the Aqueous Extract of Black Mulberry Leaves, Morus nigra, on Chlorpyrifos Toxicity in Male Albino Rats. Jordan J Biol Sci. 2019;12(4).

13. Kapoor RT. Farmers'perception about *Parthenium hysterophorus* l.-a case study of handia and phulpur tehsils of allahabad district, uttar pradesh. 2016.

14. Picman AK, Elliott R, Towers G. Cardiac-inhibiting properties of the sesquiterpene lactone, parthenin, in the migratory grasshopper, *Melanoplus sanguinipes*. Can J Zool. 1981;59(2):285-92.

15. Lakshmi C, Srinivas C. Parthenium: A wide angle view. Indian J Dermatol Venereol Leprol. 2007;73(5):296.

16. Batish DR, Singh H, Kohli R, Saxena D, Kaur S. Allelopathic effects of parthenin against two weedy species, Avena fatua and Bidens pilosa. Environ Exp Bot. 2002;47(2):149-55.

17. Batish D, Singh H, Saxena D, Kohli R. Weed suppressing ability of parthenin-a sesquiterpene lactone from *Parthenium hysterophorus*. N Z Plant Prot. 2002;55:218-21.

18. Patel VS, Chitra V, Prasanna PL, Krishnaraju V. Hypoglycemic effect of aqueous extract of *Parthenium hysterophorus* L. in normal and alloxan induced diabetic rats. Indian J Pharmacol. 2008;40(4):183.

19. Selmi S, Rtibi K, Grami D, Sebai H, Marzouki L. Rosemary (*Rosmarinus officinalis*) essential oil components exhibit anti-hyperglycemic, anti-hyperlipidemic and antioxidant effects in experimental diabetes. Pathophysiology. 2017;24(4):297-303.

20. Asghari B, Zengin G, Bahadori MB, Abbas-Mohammadi M, Dinparast L. Amylase, glucosidase, tyrosinase, and cholinesterases inhibitory, antioxidant effects, and GC-MS analysis of wild mint (*Mentha longifolia* var. calliantha) essential oil: A natural remedy. Eur J Integr Med. 2018;22:44-9.

21. Borah A, Paw M, Gogoi R, Loying R, Sarma N, Munda S, et al. Chemical composition, antioxidant, anti-inflammatory, anti-microbial and in-vitro cytotoxic efficacy of essential oil of Curcuma caesia Roxb. leaves: An endangered medicinal plant of North East India. Ind Crops Prod. 2019;129:448-54.

22. Manssouri M, Znini M, Majidi L. Studies on the antioxidant activity of essential oil and various extracts of Ammodaucus leucotrichus Coss. & Dur. Fruits from Morocco. J Taibah Uni Sci. 2020;14(1):124-30.

23. Zhang C, Zhao J, Famous E, Pan S, Peng X, Tian J. Antioxidant, hepatoprotective and antifungal activities of black pepper (Piper nigrum L.) essential oil. Food Chem. 2021;346:128845.

24. Dwivedi S, Dwivedi A, Dwivedi S. Folk lore uses of some plants by the tribes of Madhya Pradesh with special reference to their conservation. Ethnobot leafl. 2008;2008(1):105.

25. Shabbir A, Javaid A. Effect of aqueous extracts of alien weed *Parthenium hysterophorus* and two native asteraceous species on germination and growth of mungbean, Vigna radiata L. wilczek. J Agric Res. 2010;48(4):483-8.

26. Towers G, Subba Rao P, editors. Impact of the pan-tropical weed, *Parthenium hysterophorus* L. on human affairs. Proceedings of the first international weed control congress; 1992: Weed Science Society of Victoria Melbourne.

27. Espinosa-Rivero J, Rendón-Huerta E, Romero I. Inhibition of Helicobacter pylori growth and its colonization factors by *Parthenium hysterophorus* extracts. J Ethnopharmacol. 2015;174:253-60.

28. Tanvir R, Sajid I, Hasnain S. Screening of endophytic Streptomycetes isolated from *Parthenium hysterophorus* L. against nosocomial pathogens. Pak J Pharm Sci. 2013;26(2):277-83.

29. Deka B, Jiten M, Bhattacharyya P. Acaricidal activities of *Parthenium hysterophorus* L. against red spider mite, *Oligonychus coffeae* Nietner (Acarina: Tetranychidae) of tea. Int J Sci Res. 2013;4(5):2319-7064.

30. Krishnaveni M. Air pollution tolerance index and antioxidant activity of *Parthenium hysterophorus*. J Pharm Res. 2013;7(4):296-8.

31. Nabi SA, Kasetti RB, Sirasanagandla S, Tilak TK, Kumar MVJ, Rao CA. Antidiabetic and antihyperlipidemic activity of Piper longum root aqueous extract in STZ induced diabetic rats. BMC Complement Altern Med. 2013;13(1):37.

32. Panwar R, Sharma AK, Dutt D, Pruthi V. Phenolic acids from *Parthenium hysterophorus*: evaluation of bioconversion potential as free radical scavengers and anticancer agents. Adv Biosci Biotech. 2015;6(01):11.

33. Anup R, Sainath P, Padma S, Sukumaran M. In vitro cytotoxic activity of methanolic extract of *Parthenium hysterophorus* flowers on MCF-7 and HeLa cell lines. Int J Curr Microbiol Appl Sci. 2015;4(8):710-5.

34. Kumar S, Nair G, Singh AP, Batra S, Wahab N, Warikoo R. Evaluation of the larvicidal efficiency of stem, roots and leaves of the weed, *Parthenium hysterophorus* (Family: Asteraceae) against Aedes aegypti L. Asian Pac J Trop Dis. 2012;2(5):395-400.

35. Kumar VP, Chauhan NS, Padh H, Rajani M. Search for antibacterial and antifungal agents from selected Indian medicinal plants. J Ethnopharmacol. 2006;107(2):182-8.

36. Jha U, Chhajed PJ, Oswal RJ, Shelke TT. Skeletal muscle relaxant activity of methanolic extract of *Parthenium hysterophorus* L. leaves in swiss albino mice. Int J Pharm Life Sci. 2011;2(11).

37. Datta S, Saxena DB. Pesticidal properties of parthenin (from *Parthenium hysterophorus*) and related compounds. Pest Manage Sci. 2001;57(1):95-101.

38. Bharadwaja S, Singh S, Moholkar VS. Design and optimization of a sono-hybrid process for bioethanol production from *Parthenium hysterophorus*. J Taiwan Inst Chem Eng. 2015;51:71-8.

39. Parashar V, Parashar R, Sharma B, Pandey AC. Parthenium leaf extract mediated synthesis of silver nanoparticles: a novel approach towards weed utilization. Dig J Nanomater Biostructures. 2009;4(1).

40. Rajiv P, Rajeshwari S, Venckatesh R. Bio-Fabrication of zinc oxide nanoparticles using leaf extract of *Parthenium hysterophorus* L. and its size-dependent antifungal activity against plant fungal pathogens. Spectrochimica Acta Part A: Mol Biomol Spectrosc. 2013;112:384-7.

41. Yang J, Jiang F, Ma C, Rui Y, Rui M, Adeel M, et al. Alteration of crop yield and quality of wheat upon exposure to silver nanoparticles in a life cycle study. J Agric Food Chem. 2018;66(11):2589-97.

42. Ahmad MA, Yuesuo Y, Ao Q, Adeel M, Hui ZY, Javed R. Appraisal of comparative therapeutic potential of undoped and nitrogen-doped titanium dioxide nanoparticles. Molecules. 2019;24(21):3916.

43. Javed R, Ahmad MA, Gul A, Ahsan T, Cheema M. Comparison of chemically and biologically synthesized nanoparticles for the production of secondary metabolites, and growth and development of plants. Compr Anal Chem. 2021;94:303-29.

44. Ahmed M, Ji M, Qin P, Gu Z, Liu Y, Sikandar A, et al. Determination of phytochemicals, antioxidant activity and biochemical composition of chinese

mugwort (*Artemisia argyi* l.) leaf extract from northeast China. Appl Ecol Environ Res. 2019;17(6):15349-62.

45. Sparkman OD. Identification of essential oil components by gas chromatography/quadrupole mass spectroscopy Robert P. Adams. J Am Soc Mass Spectrom. 2005;16(11):1902-3.

46. McConnachie A, Strathie L, Mersie W, Gebrehiwot L, Zewdie K, Abdurehim A, et al. Current and potential geographical distribution of the invasive plant *Parthenium hysterophorus* (Asteraceae) in eastern and southern Africa. Weed Res. 2011;51(1):71-84.

47. Narasimhan T, Murthy BK, Harindranath N, Rao PS. Characterization of a toxin from*Parthenium hysterophorus* and its mode of excretion in animals. Weed Res. 1984;6(5):729-38.

48. Hasan N, Jain R. Biotoxicity of *Parthenium hysterophorus* extracts against *Meloidogyne javanica* and *Helicotylenchus dihystera*. Nematol Mediterr. 1984.

49. Attaullah ZM, Zahoor M, Mubarik M, Rizvi H, Majeed H, Zulhussnain M, et al. Insecticidal, biological and biochemical response of *Musca domestica* (Diptera: Muscidae) to some indigenous weed plant extracts. Saudi J Biol Sci. 2020;27(1):106-16.

50. Kalaiselvi M, Subbaiya R, Selvam M. Synthesis and characterization of silver nanoparticles from leaf extract of *Parthenium hysterophorus* and its anti-bacterial and antioxidant activity. Int J Curr Microbiol Appl Sci. 2013;2(6):220-7.

51. Arshad M, Mumtaz MW, Chaudhary AR, Rashid U, Ali M, Mukhtar H, et al. Metabolite profiling of Cycas revoluta leaf extract and docking studies on alpha-glucosidase inhibitory molecular targets by phytochemicals. PakJ Pharm Sci. 2019;32(2 (Supplementary)):871-4.

52. Yamaguchi KKdL, Souza AdO. Antioxidant, hypoglycemic and neuroprotective activities of extracts from fruits native to the Amazon region: A review. Biotech J Int. 2020;24(6):9-31.

53. Akbari B, Baghaei‐Yazdi N, Bahmaie M, Mahdavi Abhari F. The role of plant‐derived natural antioxidants in reduction of oxidative stress. BioFactors. 2022;48(3):611-33.

54. Sinha SN, Paul D. Antioxidant potentials of *Parthenium hysterophorus* L. leaf extracts. Sci Res J India. 2014;3(2):80-6.

55. Bashir S, Raza, S.A., Ahmad, M., Farhan, M., Ali, S. and William, J. Hydroethanolic leaf extract of *Parthenium hysterophorus* attenuates blood glucose in alloxan induced diabetic mice. Food Res. 2021;5(1):72-7.

56. Ahmad J, Bagheri R, Bashir H, Baig MA, Al-Huqail A, Ibrahim MM, et al. Organ-specific phytochemical profiling and antioxidant analysis of *Parthenium hysterophorus* L. BioMed Res Int. 2018;2018.

57. Adeel M, Zain M, Fahad S, Rizwan M, Ameen A, Yi H, et al. Natural and synthetic estrogens in leafy vegetable and their risk associated to human health. Environ Sci Pollut Res. 2018;25(36):36712-23.

58. Rajiv P, Rajeshwari S, Yadav RH, Rajendran V. Vermiremediation: Detoxification of Parthenin toxin from Parthenium weeds. J Hazard Mater. 2013;262:489-95.

59. Tesfu F, Emana G. Evaluation of Parthenium hysterophorus L. powder against *Callosobruchus chinensis* L.(Coleoptera: Bruchidae) on chickpea under laboratory conditions. Afri J Agri Res. 2013;8(44):5405-10.

60. Shaheen FA, Khaliq A. Management of Pulse Beetle, *Callosobruchus chinensis* L.(Coleoptera: Bruchidae) in stored Chickpea using ashes, red soil powder and turpentine oil. Pak Entomol. 2005;27(2):19-24.

61. Yadav SK, Patel S. Insecticidal and repellent activity of some plant extracts against *Myzus persicae* (Sulzer) and *Brevicoryne brassicae* (Linnaeus). J Entomol Zool Stud. 2017;5(2):1434-143.

62. Wasu Yogesh H GD, Patel Sagar B. Larvicidal effects of *Parthenium hysterophorus* againstpolyphagus pests *Spodoptera Litura* and *Spodopteralittoralis*. Indian J Appl Res. 2019;9(2):1-19.

63. Wang M-H, Gan J-J, Chen W-H, Guan Y-L. Chemical Constituents of Parthenium hysterophorus. Chem Nat Comp. 2020;56:556-8.

64. Białoń M, Krzyśko-Łupicka T, Nowakowska-Bogdan E, Wieczorek PP. Chemical composition of two different lavender essential oils and their effect on facial skin microbiota. Molecules. 2019;24(18):3270.

65. Hudz N, Makowicz E, Shanaida M, Białoń M, Jasicka-Misiak I, Yezerska O, et al. Phytochemical Evaluation of Tinctures and Essential Oil Obtained from Satureja montana Herb. Molecules. 2020;25(20):4763.

66. Korzeniowska K, Łęska B, Wieczorek PP. Isolation and determination of phenolic compounds from freshwater *Cladophora glomerata*. Algal Res. 2020;48:101912.

67. Bajwa AA, Weston PA, Gurusinghe S, Latif S, Adkins SW, Weston LA. Toxic Potential and Metabolic Profiling of Two Australian Biotypes of the Invasive Plant Parthenium Weed (*Parthenium hysterophorus* L.). Toxins. 2020;12(7):447.

68. Koo S, Thomas HR, Daniels SD, Lynch RC, Fortier SM, Shea MM, et al. A breath fungal secondary metabolite signature to diagnose invasive aspergillosis. Clin Infect Dis. 2014;59(12):1733-40.

69. Yang D, Michel L, Chaumont J-P, Millet-Clerc J. Use of caryophyllene oxide as an antifungal agent in an in vitro experimental model of onychomycosis. Mycopathologia. 2000;148(2):79-82.

70. Saifullah K. Callus induction and cell suspension culture production of *Catharanthus roseus* for biotransformation studies of (-)-caryophyllene oxide. Pak J Bot. 2011;43(1):467-73.

71. Halim SA, Uddin R, Madura JD. Benchmarking docking and scoring protocol for the identification of potential acetylcholinesterase inhibitors. J Mol Graph Model. 2010;28(8):870-82.

72. Mulyaningsih S, Sporer F, Reichling J, Wink M. Antibacterial activity of essential oils from Eucalyptus and of selected components against multidrug-resistant bacterial pathogens. Pharm Biol. 2011;49(9):893-9.

Tables

Table 1. Physical appearance of the flower's extract of *Parthenium hysterophorus*.

Extract	Visibility	Physical Nature	Consistency
EtOH	Bluish green	Oily	Consistent
EA	Greenish	Oily	Consistent
DCM	Brownish	Oily/Gummy	Non-consistent
DW	Brownish	Oily/Gummy	Non-consistent

EtOH; (Ethanol), EA; (Ethyl Acetate), DCM; (Dichloromethane), DW; (Distilled water).

Table 2. Total Phenol, Flavonoid content and DPPH radical scavenging activity of the flower's extract of *Parthenium hysterophorus*.

Extracts	Phenol (GAE/g)	Flavonoid (QE/g)	DPPH Inhibition (%)
EtOH	27.55 ± 1.46^{c}	14.23 ± 0.10^{b}	88.53 ± 0.11^{b}
EA	47.79 ± 0.85^{b}	138.67 ± 2.20^{a}	67.21 ± 0.19^{c}
DCM	30.50 ± 0.00^{d}	15.38 ± 0.04^{c}	95.03 ± 1.58^{a}
DW	103.30 ± 1.16^{a}	4.81 ± 0.01^{c}	18.73 ± 0.89^{d}
Statics	S.S= 24268	S.S= 248063	S.S= 13880
	M.S=8069	M.S= 82687	M.S= 4626
	Df= 3	Df= 3	M.S= 3
	f= 2564***	*f*= 22488***	*f*= 1031***

Data in the columns is described as mean values ± standard deviations with various superscripts are significantly different according to DMRT" *P*> 0.05). S.S (Sum of square); M.S (Mean square); Df (Degree of freedom); *f* (Significance); *** (significance level).

Table 3. Correlation of the antioxidant activity of *P. hysterophorus* extract versus Phenols, Flavonoids and extract yield.

Treatments	Phenols	Flavonoids	DPPH	Yield
Phenols	1	-0.16	-0.99**	-0.11
Flavonoids	-0.16	1	-0.71	-0.90
DPPH	-0.99**	-0.71	1	-0.10
Yield	-0.11	-0.09	-0.10	1

Note: * Correlation is significant at 0.05 levels; ** Correlation is highly significant at 0.05 levels

Table 4. **Toxicity of *P. hysterophorus* extracts against *B. brassicae* at 12, 24, 48, 72 and 96 hours exposure**

Time	LC_{50} μgmL^{-1}	95% F.L		Slope± S.E	χ2
		Lower	Upper		
12	3598	1421	35512	1.09±0.38	0.25
24	2569	1321	27758	1.51±0.46	0.99
48	2345	1153	25517	1.31±0.53	0.85
72	1446	852	21721	1.56±0.63	0.22
96	750	468	1276	1.89±0.58	0.04

Note: LC_{50} (Lethal concentration); S.E. (standard error); χ2 (chi-square); F.L. (Fiducial limit)

Table 5. Chemical composition of the flower's extract of *Parthenium hysterophorus*.

Peak #	R. T	Area %	Chemical Compounds	M.F	M.W g/mol
1	3.22	7.05	Benzene, 1,3-dimethyl-	C_8H_{10}	106.17
2	3.92	3.47	2,4-Dimethylamphetamine	$C_{11}H_{17}N$	163.26
3	4.43	2.04	3-Piperidinol	$C_5H_{11}NO$	101.15
4	5.05	2.74	2-Pentene, 4,4-dimethyl-, (E)-	C_7H_{14}	98.186
5	5.52	3.55	2-Butanamine	$C\ _6H\ _5N$	101.19
6	12.96	25.93	2-Naphthalenemethanol,decahydro.alpha.,.α.,4a-trimethyl-8-methylene-,[2R (2 α.,4a.α.,8a.beta.)]-	$C_{15}H_{26}O$	222.36
7	14.29	11.27	3-Methoxy-3,4-dimethyl-1-heptyne	$C_{10}H_{18}O$	154.25
8	15.21	4.76	Undecane	$C_{11}H_{24}$	156.31
9	15.85	3.82	2-Pyrrolidinone, 1-methyl-	C_5H_9NO	99.13
10	16.62	7.26	Glycinamide hydrochloride	$C_2H_7C_lN_2O$	110.54
11	17.58	2.18	1,2-Benzenedicarboxylic acid, butyl 2-ethylhexyl ester	$C_{20}H_{30}O_4$	334.45
12	18.1	6.67	Hexadecanoic acid, ethyl ester	$C_{18}H_{36}O_2$	284.48
13	21.85	19.25	Histidine, 1,N-dimethyl-4-nitro-	$C_8H_{10}N_2O_2$	166.18

R.T (Retention time); M.F (Molecular formula); M.W (Molecular Weight).

Figure Click here to access/download;Figure;Figures R.docx

Figure legends

Fig 1. Yield of extract obtained by using different solvents. Data presented in bars is described as mean values ± standard error with superscripts is significantly different according to DMRT” *P*> 0.05). EtOH; (Ethanol), EA; (Ethyl Acetate), DCM; (Dichloromethane), DW; (Distilled Water).

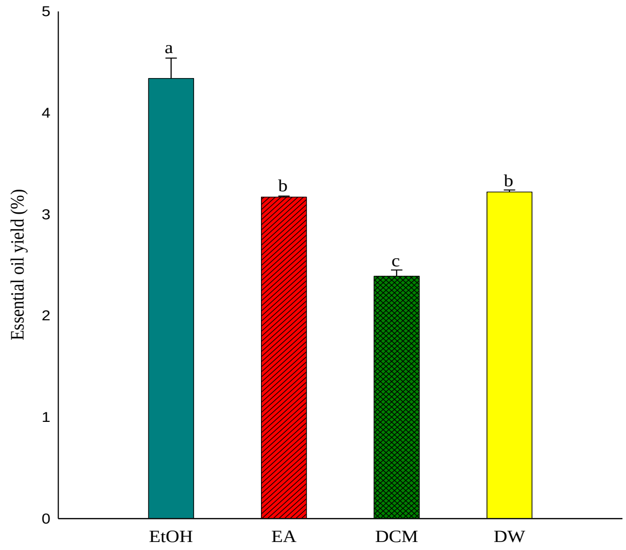

Fig 2. Insecticidal activity of extract of *Parthenium hysterophorus* flowers. Data in the bars is described as mean values ± standard error with various superscripts is significantly different according to DMRT" P> 0.05). Whereas; A (100 µgmL-1); B (200 µgmL-1); C (400 µgmL-1); D (800 µgmL-1); E (1000 µgmL-1); F (-iv Control); G (+iv Control).

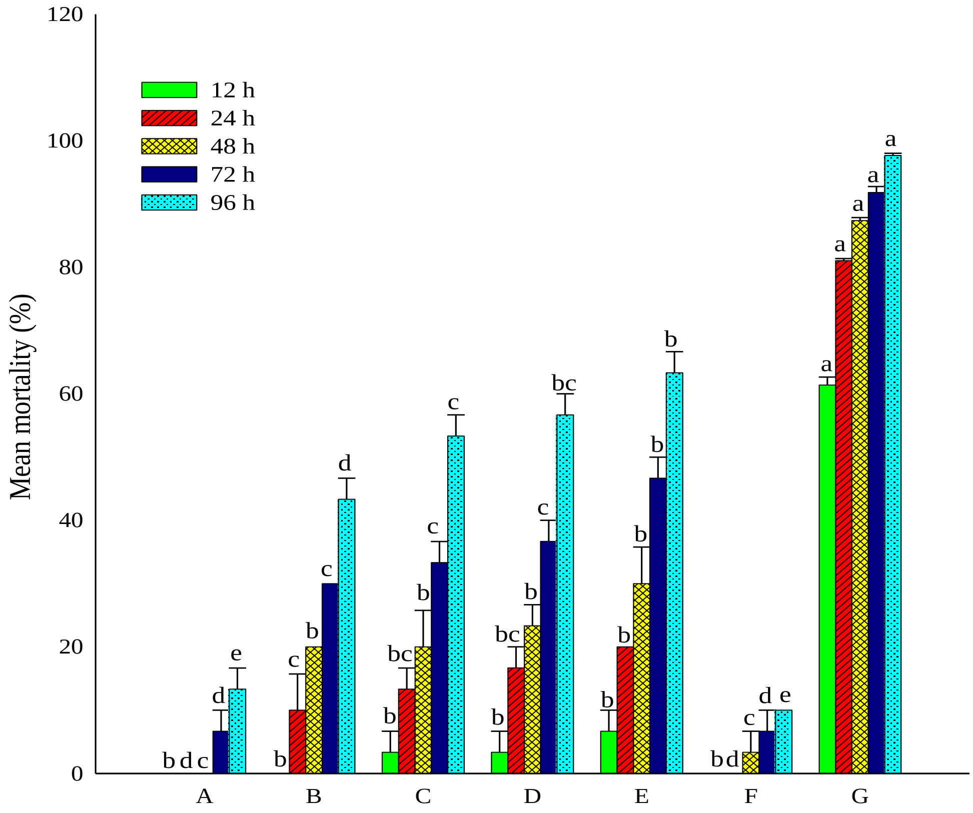

Fig 3. GC-MS chromatogram of *Parthenium hysterophorus* flower's extract.

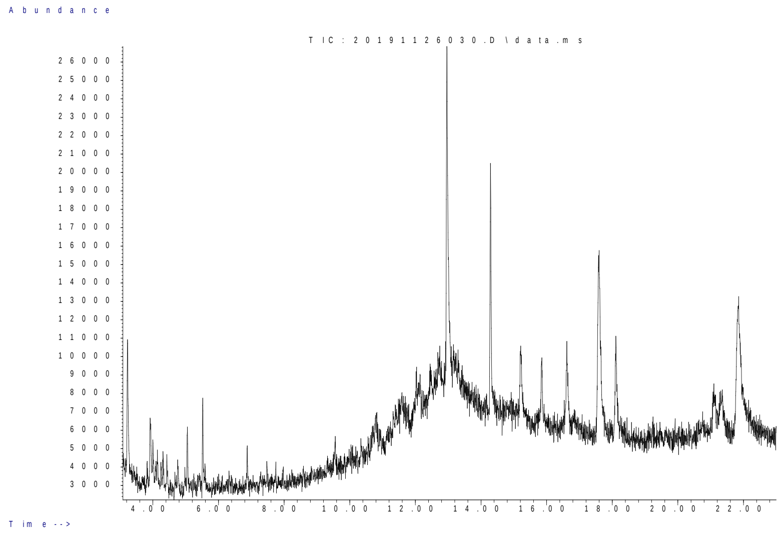

Nextgenediting Template PLOS_One_Word_Template.docx
www.nextgenediting.com [double click header to delete]

Original Manuscript

Antioxidant, Insecticidal Activity and Chemical Profiling of Flower's Extract of Carrot Grass (*Parthenium hysterophorus* L.)

Maqsood Ahmed[1,2], Ansar Javeed[3], Samiullah[2], Talfoor-ul-Hassan[4], Aatika Sikandar[1], Taswar Ahsan[5], Faqir Ahmed[6], Ji Mingshan[1], Xuejing Bai[7], Ying Li[8] and Zumin Gu[1,*]

[1]College of Plant Protection, Shenyang Agricultural University, Shenyang, 110866, P.R. China. aatika_sikander@yahoo.com (A.S.); jimingshan@syau.edu.cn (M.J.)
[2]Department of Agriculture (Plant Protection), Pest Warning and Quality Control of Pesticides, Gujrat, 50700, Pakistan. maqsoodahmed200@hotmail.com
[3]Henan University, Jinming Campus, Kaifeng City Henan China. ansarjaveed@henu.edu.cn (A.J)
[4]Department of Agriculture (Plant Protection), Pest Warning and Quality Control of Pesticides, Gujranwala, 52250, Pakistan. ddappgujranwala@gmail.com (T.u.H.)
[5]Institute of Plant Protection Liaoning Academy of Agricultural Sciences, Shenyang, 110866, P.R. China. taswarahsan@163.com (T.H)
[6]Directorate General of Agriculture, Pest Warning and Quality Control of Pesticides, Lahore, 54800, Pakistan. faqirahmad63@yahoo.com (F.A)
[7]Shenyang Academy of Landscape- Gardening Shenyang 110016, P.R. China. baixuejingnv@163.com (X.B.)
[8]Fushun Modern Agriculture and Poverty Alleviation Development Promotion Centre, Fushun 113006 P.R. China. liying_1973@163.com (Y.L.)

Corresponding author: *guzumin1212@163.com *(G.Z); Tel: +86-24- 8848- 7148; Fax: +86-24-8834-2315*

Abstract

Parthenium hysterophorus L. an invasive alien species is considered a noxious weed and confers numerous benefits in medical and agrochemical industries. The present study evaluated the flower extract's antioxidant and insecticidal activity and chemical profiling to appraise the existence of phytoconstituents responsible for biological activities. Assay was performed by 1,1-diphenyl-2-picrylhydrazyl (DPPH) for antioxidant activity, whereas, chemical configuration was evaluated by Gas Chromatography Mass Spectrometry (GCMS) analysis. Our findings report that the extract of *P. hysterophorus* possesses potential radical scavenging activity (95.03%) when extracted via dichloromethane (DCM). Additionally, phytochemical analysis revealed a considerable amount of phenols and flavonoid contents when extracted via distilled water ~~(D.W)~~ and ethyl acetate~~ (E.A)~~; (103.30 GAE~~/~~g^{-1} and 138.67 QE~~/~~ g^{-1}) respectively. Maximum mortality 63.33% and 46.67% was observed at 1000 µgmL^{-1} and 800 µgmL^{-1} after 96 h exposure followed by 46.67% and 36.67% at 72 h at the same concentration. On the other hand, toxicity results displayed that *P. hysterophorus* extracts showed LC_{50} values of 1446 and 750 µgmL^{-1} at 72 and 96 h exposure, respectively. However, Imidacloprid being a positive control afforded maximum mortality at 96 h and 72 h exposure 97.67% and 91.82%, respectively. Moreover, antioxidant activity of *P. hysterophorus* extracts displayed strong correlation versus phenols, flavonoids and extract yield. GCMS analysis revealed the existence of 13 chemical compounds corresponding to 99.99% of whole extract. Moreover, maximum extract was afforded by Ethanol ~~(EtOH)~~ followed by distilled water, ethyl acetate and dichloromethane ~~D.W, E.A and DCM~~ as 4.34, 3.22, 3.17 and 2.39%, respectively. *P. hysterophorus* flower's extract therefore showed good antioxidant and aphicidal activity which also exhibited valuable chemical compounds accountable for biological activity, making them suitable candidates for an alternative to synthetic agents. However, our results provide novel and fundamental basis for further exploration on purification of chemical compounds for their biological activities.

Keywords: *Parthenium hysterophorus*; phytochemicals; Antioxidant activity; GCMS; DPPH

Introduction

~~The World Conservation Union stated second utmost substantial threat to biodiversity and habitat are invasive alien species. Moreover, they exert~~ The invasive alien species exert devastating impacts on ~~innate~~inherent biota and deleteriously affecting ecosystems by destructions of native species. Likewise, such distressing impacts on natural biodiversity could lead towards escalation of climate change, environmental pollution, and ecological destruction of natural habitat. However, identifying the beneficial~~ts~~ and harmful impacts of *Parthenium hysterophorus* ~~being~~ introduced as an invasive alien species, might lead towards ~~better understanding and~~ control of biological invasions. ~~In brief, the benefits and drawbacks are contradictory towards the sides of the same coins.~~ Invasive alien plant species are the worldwide risk to biodiversity, functioning of ecosystem and farm~~ed~~ production [1]~~ Adkins, Shabbir ([1]~~. Invasive weeds species are undesirable self-grown plants that compete with the cultivated and non-cultivated crops for nutrients, sunlight and water. They also produce a wide range of bioactive particles that are rich sources of various types of medicines. ~~Today~~Currently, most of the medications in the pharmaceutical industry depend upon natural plants and or in the herbal traditional systems [1].

Parthenium hysterophorus L. is an extremely invasive plant species belonging to family Asteraceae that has invaded almost fifty countries in sub-continent Africa, Asia, and Oceania, infesting forests, feeding lands, and urban landscapes [2, 3]. It is a devastating and hazardous weed of various economically important crops [4]. ~~(Ray and Gour, 2012).~~ Moreover, it is amongst the top ten worst weeds in the world and is included in the world catalogue of destructive species [5, 6]. ~~(Kapoor, 2012; Khan et al., 2020).~~ In Europe *P. hysterophorus* is considered as an ephemeral species. ~~*P. hysterophorus*~~ It is generally recognized by different names such as carrot grass,

Formatted: Font: Font color: Black, English (United States), Highlight

Formatted: Font: Font color: Black, English (United States), Highlight

Formatted: Font: Font color: Black, English (United States), Highlight

congress grass, Gajjar botti, white head and white top [7]. It is native to Gulf of Mexico and United States. However, it accidently introduced in Indian subcontinent [8]. This weed has been considered as a major weed of high rank ~~already taken major rank as many indigenous weeds and is~~ spreading at a much faster pace to takeover abandoned land, canal, watercourse banks, roads, and fields to challenge crop productivity [9, 10]. ~~[Jabeen et al. 2022; Arshad Javaid et al 2022]~~.

Medicinal plants are the fundamentals of traditional therapeutic systems based on the trusts and observations comprised of hundreds of years [11]. The phytochemical constituents such as phenolic and flavonoid are secondary metabolites involved in therapeutic system, because of the existence of these functional phytochemicals mainly parthenin [12]. Moreover, *P. hysterophorus* renowned to contain vital biochemical compounds such as alkaloids, tannins, and saponins, which are important from the medicinal viewpoint [13]. It also exhibited allelopathic effects and important phytochemicals such as parthenon and coronopilin which are auto-toxic to its own seed germination and seedling growth [14]. Furthermore, serious health consequences ~~of health~~ on human beings and livestock have been reported [15]. The a~~A~~griculturists who are come in contact ~~associated~~ with *P. hysterophorus* ~~because~~; the pollen and dust produced by this weed provoke allergic contact dermatitis in human beings. Major chemical compounds, parthenin's contained by *P. hysterophorus* exhibited phytotoxic properties against a variety of plants, including weeds and various crops such as Slender amaranth (*Amaranthus viridis*), Billygoat weed (*Ageratum conyzoides*), common wild oat. (*Avena fatua*), nettleleaf goosefoot (*Chenopodium murale*), Foetid cassia (*Cassia tora*), mung bean *Phaseolus aureus* [16, 17]. Moreover, parthenin's the main biochemical compounds showed insecticidal activity against termites, cockroaches and migratory grasshoppers, *Melanoplus sanguinipes*

Formatted: Font: English (United States), Highlight

Formatted: Font: Font color: Black, English (United States), Highlight

Formatted: Font: English (United States), Highlight

[7]. Similarly, Patel and Chitra [13] Patel, Chitra (18) reported pesticidal activity of the ethanol extract of *P. hysterophorus* which showed substantial lethal effect against stored grain pest like *Tribolium* sp. and *Oryzaephilus* sp. Essential oils from the natural plants also have antioxidant effects. In recent years several reports have been breakout, the essential oil from Rosemary [19], wild mint (*Mentha longifolia* var. *Calliantha*) [20], black turmeric (*Curcuma caesia*) [21] *Ammodaucus leucotrichus* [22] and black pepper (*Piper nigrum*) [23].

Pharmacologically, it is involved in treating numerous diseases like allergies, anemia, sores, skin diseases, facial neuralgia, fever, vermifuge, blood purifier, and insecticide [7, 24]. This plant species is enriched with vital phytoconstituents however; sesquiterpene lactones are the most copious phytochemicals exhibited by this plant which are accountable for injurious effects such as strong allelopathic [25], toxic to livestock [26], It also possess many advantageous effects like antibacterial [27, 28], acaricidal [29], antioxidant [30, 31], cytotoxic [32, 33], larvicidal potential against *Aedes aegypti* L. [34, 35], skeletal muscle relaxant [36], pesticidal agent [37], producing bioethanol [38], as reducing agent in the formation of silver nanoparticles [39] and in production of zinc nanoparticles [40]. However, various factors ~~effect~~affect the biological profile of the plants and from some recent studies it has been reported that nanoparticle influenced the physiological and biological properties of the plants. It has been reported that metal-based nanoparticle showed a decrease in protein and amino acid and micronutrient contents in wheat crop grown under such conditions [41]. Moreover, nitrogen doped titanium dioxide nanoparticle enhanced antioxidant activity via reducing power assay and percent inhibition of DPPH against *Streptomyces* [42]. Likewise, synthetically and biologically prepared nanoparticle

significantly exerted effects on plant growth and secondary metabolites which ultimately triggered the biological activity of the plants [43].

As some earlier researches have been conducted on this plant species but, detailed studies on chemical and quantitative phytochemical analysis and assessment of antioxidant activities from different solvents extract as well as aphicidal activity against cabbage aphid *Brevicoryne brassicae* from flower's extracts has not been explored before. Hence, keeping in view the diverse biological properties of *P. hysterophorus*, current study was designed to determine the antioxidant and insecticidal activity of flower's extract against *B. brassicae* with appraisal of quantitative phytochemical analysis as well as assessment of chemical profiling of extract. at Biopesticide Lab of Plant Protection College Shenyang Agricultural University, Shenyang Liaoning China during the year 2019.

Materials and Methods

Collection and Samples Preparation

The whole plant samples were collected from District Sialkot Punjab Pakistan during the month of June-July 2019 and were identified as *Parthenium hysterophorus* L., from Ayub Agricultural Research Institute (AARI) Faisalabad Pakistan by Dr. Dilbar Hussain. Flowers were manually separated from the plants and left over for 20 days to dry under shade. These dried flowers were powdered by electric grinder and then extracted with different solvents (Ethanolethanol, Ethyl ethyl Acetateacetate, Dichloromethane dichloromethane and Distilled distilled water) by cold extraction method for seventy two hours at fixed temperature 28°C and at 100 rpm in an incubator shaker (ZWY-1102C). Extracted contents were filtered and concentrated to lessen the volume on rotary evaporator model R-210 BUCHI Labortechnik AG, CH-

9230 Flawil 1/Switzerland). Obtained extract's yield was measured using following Equation (Eq. 1).

$$\text{Yield (\%)} = \frac{\text{Weight of the extract}}{\text{Weight of the dried sample}} \times 100\% \qquad \text{(Eq. 1)}$$

Quantitative Analysis for Total Phenolic and Flavonoids Content

To assess the phytochemicals quantitatively, 1 mg of extract from each solvent was dissolved in 1 mL Methanol separately. Consequent mixture was vortexed and separated for analysis. For the assessment of total content for phenols Folin–Ciocalteu reagent test was used. In brief, 1 mL solution (1 mgmL^{-1}) was added to 2.5 mL of (10%) Folin–Ciocalteu with supplementary addition of 2 mL solution of (2%) sodium carbonate (Na_2CO_3). The consequential blend was incubated for fifteen min in the dark to measure absorbance at 765 nm in 96-well ELISA plate using (SpectraMax 190, Meigu molecular International Co. Ltd., Shanghai China). To build a standard curve, Gallic acid was used (1 mgmL^{-1}) at different concentrations such as 1, 0.50, 0.25, 0.10, 0.05, 0.02, 0.01, 0 mg mL^{-1} to describe the obtained results as Gallic acid equivalent (GAE) mg g^{-1} of total extract. Each treatment was replicated ten times to analyze the data.

To examine the total flavonoids content, aluminium chloride colourimetric method was adopted. Briefly, 1 mL solution (1mgmL^{-1}) was poured into 3 mL methanol, with addition of 0.2 mL of 1 M Potassium acetate (CH_3COOK), 0.2 mL of (10%) aluminium chloride ($AlCl_3$) and at the end, distilled water 5.6 mL was added to the consequent mixture. The incubation of solution was performed for half an hour in the darkness and absorbance at 420 nm was measured. A standard curve was built using quercetin at different concentrations such as 1, 0.50, 0.25, 0.10, 0.05, 0.02, 0.01, 0 mgmL^{-1} to represent the results as quercetin equivalent (QE) mg g^{-1} of total extract. The procedure was replicated 10 times for each treatment.

Assessment of Free Radical Scavenging Activity

Antioxidant activity of extracts obtained by different solvents from *Parthenium hysterophorus* flowers was accomplished in 1% solution of tween 20 by using 1,1-diphenyl-2-picrylhydrazyl (DPPH) ($C_{18}H_{13}N_5O_6$) a free radical. In brief, to assess the free radical scavenging activity, all dried solvent extracts were mixed together in equal proportion (5 mg each) and were dissolved in HPLC grade methanol and filtered. The filtrate was formerly used to assess the antioxidant activity. Similarly, DPPH solution was prepared in same grade Methanol @ 0.001g 25 mL^{-1}. Then, into 3.5 mL freshly prepared DPPH solution and 0.25 mL extract prepared in Methanol was added. The consequent mixture was shacked for complete mixing and then the resulting mixture was incubated in darkness at 28°C for 30 min [44]. After incubation of resulting mixture for stipulated period of time, the absorbance was assessed at 517nm by using (SpectraMax 190, Meigu molecular International Co. Ltd., Shanghai China). The inhibition percentage of primed DPPH solution was predicted on reduction of absorbance through following Equation (Eq. 2). Each treatment was replicated ten times to analyze the data.

$$\text{Inhibition (\%)} = \frac{A_{blank} - A_{sample}}{A_{blank}} \times 100 \qquad \text{(Eq. 2)}$$

Where: A_{blank} = (control absorbance); A_{Sample} = (samples absorbance).

Correlation of Antioxidant Activity versus Phenols, Flavonoids and extract yield

Correlation of antioxidant activity *P. hysterophorus* versus Phenols, Flavonoids and extract yield was carried out using SPSS statistics 25.0 version with significant values at (P>0.05) level.

Assessment of Insecticidal Activity

Residual toxicity method was adopted for the assessment of insecticidal activity of extract of *P. hysterophorus* flowers. Samples for bioassay study were prepared to

obtain mother mixture by mixing 0.1g of each solvent's extract and dissolved in Acetone, because it is aprotic solvent having intermediate polarity comparable to polar parotic solvents and it also evaporates immediately from the extract when exposed to air. The resulting mixture was placed in the fume hood for six hours for complete drying of Acetone. Serial concentrations *viz*. 100, 200, 400, 800 and 1000 µg mL^{-1} ~~were~~was prepared in Tween-20 (1%) solution from mother mixture. Briefly, fresh cabbage leaf discs 5cm in diameter were cut off and dipped for 10 s in the respective concentration and then dried for 5 min at room temperature. Formerly, 10 adult wingless aphids were carefully released onto the leaf disc using a fine-haired brush contained in the Petri dish and incubated for 96 h at 65% R.H., 25°C and with a 16:8 (light: dark) photoperiod. Imidacloprid 25% WP was used as the positive control at a rate of 0.0025 mL mL^{-1} of water, and control (CK) with a 1% Tween 20 solution. Positive controls and CKs were placed in a greenhouse for 96 h. Mortality data was collected at 12, 24, 48, 72 and 96 h using binocular microscope and the response of aphids was observed by needle probing, and the aphids who offered no response on stimulation were considered dead. Each treatment was replicated five times.

Chemical Analysis

Samples for analysis were prepared by mixing 0.1 g of each solvent extract into 2 mL of HPLC grade methanol to obtain mother solution. Consequent mixture was vortexed for 1 min following the addition of graphitized carbon black "GCB" (0.005 g). Mixture was centrifuged for 10 min at 10000 rpm at 25°C and colorless material was separated and collected in (1.5 mL) centrifuges tubes. Work for GC-MS analysis was accomplished using an Agilent Model 6890-5973N accompanied with gas chromatograph established on HP1 capillary column TG-5MS polydimethylsiloxane (length 30m × diameter 250 µm × film thickness 0.25 µm) interfaced with mass

selective detector Hewlett Packard (5973N). The established parameters were; preliminary temperature was adjusted at 70°C for 0 min however, final temperature was extended to 200 °C for 10°C min^{-1} whereas, inlet temperature was adjusted at 250°C with split ratio 10:1. MS quadruple temperatures were fixed at 150°C while thermal aux temperatures were 285°C. The Scan range for MS was 35–520 units however; as a carrier, helium gas was used bearing stream frequency of 1.0mLmin^{-1}. The existing compounds in the extract were identified by comparison of their retention time and the mass spectra of the NIST 08 and Wiley 7 of the data base and confirmed by comparing with GC-MS literature data at NIST and TMS database Wiley/NIST.1998.1 [45].

Statistical Analysis

All calculated data was analysed by one way analysis of variance (ANOVA), mean difference between treatments was calculated for significance test by Duncan Multiple Range Test "DMRT " at $P > 0.05$) with IBM-SPSS statistics 25.0 version software. Probit analysis was performed using EPA Probit analysis program version 1.5.

Results

The current study carried out on the flower's extract of *Parthenium hysterophorus* revealed the existence of medicinally and agro-chemically active compounds. The phytochemical constituents of *P. hysterophorus* were qualitatively analyzed for antioxidant and insecticidal activities.

Extract Yield (%)

Yield of extract by the solvent extraction technique from *P. hysterophorus* flowers was calculated and presented in (Figure 1). The maximum extract was obtained by

ethanol (4.34%) whereas; minimum extract quantity (2.39%) was afforded by dichloromethane. The extract obtained via ethanol and ethyl acetate was bluish green and greenish in color and exhibited consistency, respectively. The same oil was brownish in color and non-consistent via dichloromethane and distilled water, respectively, (Table 1).

Analysis for Phenol and Flavonoid and Antioxidant Activity

The total contents for phenol and flavonoid as well as DPPH scavenging activity afforded by *P. hysterophorus* flower's extract via different solvents are presented in (Table 2). Results confirmed that the ~~maximum~~ highest total phenol were reported from distilled water 103.30 GAE/g followed by Ethyl acetate and Ethanol' extract, 47.79 and 27.55 GAE/g, respectively. On the other hand ~~maximum~~ highest flavonoid was reported from Ethyl acetate extract 138.67 QE/g followed by Ethanol 14.23 QE/g respectively.

Inhibition (%) was determined by 1,1-Diphenyl-1-picrylhydrazyl (DPPH) displayed color absorption at 517nm on using spectrophotometer. When DPPH trap free radicals, the color of the solution changed to light yellow and, subsequently, resulted in decrease of the absorbance. Maximum DPPH radical scavenging activity was afforded by Dichloromethane extract 95.03% followed by Ethanol and Ethyl acetate 88.53% and 67.21%, respectively.

Correlation of Antioxidant Activity versus Phenols, Flavonoids and extract yield

In our study, the Pearson's correlation regarding antioxidant activity of *P. hysterophorus* showed positive relationship (Table 3). Results showed highly significant ($P<0.01$) negative effect of phenol with DPPH as well as DPPH showed strongly highly significant negatively correlation and similar results were found

reciprocally in the studied parameters. However, yield extracted by different solvents did not affect the Antioxidant Activity versus phenols, flavonoids quantitatively.

Assessment of Insecticidal Activity

The mortality data of cabbage aphid *B. brassicae* at specific period of time using *P. hysterophorus* flower's extract is presented in (Figure 2). It was perceived that the percent mortality was directly correlated to the concentration and the time. The results demonstrated that the maximum mortality was recorded at 1000 µgmL^{-1} at 96 h and 72 h with 63.33% and 46.67%, respectively. Same trend of mortality was also observed at 800 µgmL^{-1} with 56.67% and 36.67%, respectively. Imidacloprid 25% WP being a positive control produced the highest rates of mortality after 96 h of exposure 97.67%. Likewise, Imidacloprid also produced significant mortality after 72 h and 48 h exposure 91.82% and 87.38% mortality, respectively.

Analysis for probability of the data showed the LC_{50} values, slope, chi-square, and fiducial limits at confidence interval of 95% for *P. hysterophorus* extracts. Mortality response using plant extracts showed sensitivity of *B. brassicae* to various concentrations which is represented in (Table 4).

Chemical Analysis

The existence of chemical compounds in *P. hysterophorus* flower's extract was analyzed by GCMS techniques and data is presented in (Table 5). Thirteen biochemical compounds equivalent to 99.99% of entire extract by GC fraction were identified. Moreover,2-~~Naphthalenemethanol~~naphthalenemethanol,decahydro-.alpha.,.alpha.,4a-trimethyl-8- methylene-, [2R--(2.alpha., 4a.alpha.,8a.beta.)]-(25.93%), ~~Histidine~~histidine, 1,N-dimethyl-4-nitro- (19.25%) and 3-~~Methoxy~~methoxy-3, 4-dimethyl-1-heptyne (11.27%), were the major chemical

compounds while, 10 others chemical compounds were identified in minor amounts having areas with peak range 2.04–3.7~~.05~~%. GC-MS chromatogram of *P. hysterophorus* flower extract is presented in (Figure 3).

Discussion

~~The extractives are comprised of secondary compounds metabolized by natural plants are noxious which stored by plant cells vacuoles. Such~~ The chemical compounds include phenolic, steroid, alkaloids, flavonoids and terpenoids depends their concentration on ecological conditions and occurrence of the pathogens. They also possessed bio-pesticidal properties and are target specific against insect pests and pathogens. Moreover, these compounds are biodegradable, without triggering severe harm to the concerns of the environment [27, 46-48].

Appraisal of antioxidant and polyphenols activity of medicinal products has become vital device to recognize the medicinal possessions of natural plants. An antioxidant is a molecule which exhibited the ability to inhibit the oxidation of other chemical elements. Kumar and Mishra [44] ~~Kumar, Mishra [44](44)~~ reported that alkaloids, flavonoids, terpenoids, and cardiac glycosides from the extract of *Parthenium hysterophorus*. However, the phenolic contents in flower extracts were ranging from 86.69-320.17 mg propyl gallate equivalent (PGE)/g. Study established that bio-chemicals possessed by *P. hysterophorus* extracts bears significant antioxidant activity consistent to our current findings. Similarly, 50% radical scavenging potential of *P. hysterophorus* extract at 250 µgmL^{-1} using DPPH assay as well as 30% inhibition at 100 µgmL^{-1} via hydrogen per oxide assays was reported from *P. hysterophorus* leaves extract [49]. Antioxidant activities of hydro-ethanolic

extracts of *P. hysterophorus* leaves was perhaps because of the existence of polyphenols contents [11, 50].

Likewise, Sinha and Paul (51) found that extract from *P. hysterophorus* possess valuable antioxidant activity with 54.5% scavenging property and 60.2 IC_{50} via DPPH assay while, 93.2% and 33.6 IC_{50} via ABTS assay which are consistent with our findings on antioxidant activity. Bashir et al. [52] ~~Bashir (52)~~ evaluated leaf extract of *P. hysterophorus* for the presence of total phenolic and flavonoid contents as 105.44mg GAE/g and 41.50mg RE/g respectively exhibited by 60% ethanolic extract with IC_{50} value of 87.55 μgmL^{-1} and 98.22 μgmL^{-1} for DPPH radical scavenging activity respectively supported our investigations on antioxidant as well as phenol and flavonoid contents from *P. hysterophorus*. Phytochemical profile of *P. hysterophorus* root, stem, leaf and phyllary identified terpenes, fatty acids, hydrocarbons and phytosterols however; root extracts showed highest antioxidant potential [53]. The results obtained on antioxidant activity of *P. hysterophorus* are in agreement with Sinha and Paul (51) who reported that its leaves crude extract bears considerable antioxidant activity being IC^{50} 60.24 and 33.60% by DPPH and ABTS method respectively.

As it is evident from previous findings that exposure of natural fauna to harmful chemicals or particle can altered the biological activities, consequently, sometimes estrogen compounds cause acute toxicity in edible crops and vegetables which may occur via long-term application of estrogen-containing irrigation water. Comparable results were reported by Adeel and Zain [54] ~~Adeel, Zain (54)~~ that uptake and accumulation of l7β-estradiol (17β-E2) and ethinylestradiol (EE2) in lettuce (*Lactua sativa*) grown under lab conditions.

The historic practice of using ~~Nicotine~~ nicotine and ~~Pyrethrum~~ pyrethrum for pest control measures has encouraged scientists to draw their attention to secondary metabolites like terpenoids, alkaloids, and flavonoids as pest regulator agents [55]. So, the outcomes of the current exploration are in agreement with other workers like [56] who evaluated different parts of *P. hysterophorus* extract against *Callosobruchus chinensis* and establish that 73.33% mortality was caused after 96 h exposure followed by 76.67% and 56.67% via inflorescence powder and stem powder respectively. Moreover, their leaves extract afforded repellent affects as 70–100% for oviposition performance of the *Aedes aegypti* adults. However, the diethyl ether extracts resulting in extreme repellency as 99.7%.

The insecticidal impact of plant extracts may attribute to the following belongings including repellency and stomach poisoning might decrease insect movement and also cause death through constriction of the insect spiracles [57]. A study reported the chemical examination of parthenium has specified that whole plant exhibited toxins called as sesquiterpene lactones responsible for insecticidal activities however; the major constituent of these toxins is parthenin derivatives. These derivatives showed insecticidal as well as nematicidal activity against the *Callosobruchus maculatus* adults and root knot nematode *Meloidogyne incognita*. Similarly, the pyrazoline was found highly effective being an insecticide, with LC_{50} value 32 mgl^{-1} with exposure of 72 h, and found extremely effective as nematicidal activity with LC_{50} values being 512 mgl^{-1} perceived for parthenin at 72 h exposure [37].

Parthenium *hysterophorus* ~~leave's~~ extract used against mustard aphid, *Lipaphis erysimi* under field conditions exhibited incredible decrease of 29% of the infestation which may be because of the effect of phenolic acids [58]. Ahmad and Bagheri [53] ~~Ahmad, Bagheri (53)~~ reported the efficacy of *P. hysterophorus* extract with 10 and

20% concentration found 26.70% and 27% mortality at 21 day after treatment. Consequently, Wasu and Yogesh [59] Wasu Yogesh H (59) in their study reported the activity of leaves and stem extract of *P. hysterophorus* against different larval instars of *Aedes aegypti*. Stored stock and freshly prepared solution caused 80% and 100% mortality, respectively with concentration prepared in 200g/500 ml. Another study reported that crude aqueous extracts of whole plant of *P. hysterophorus* caused a significant mortality with 73.33, 86.67 and 83.33% of *B. brassicae* as well as moderate mortality being 26.67, 46.67 and 63.33% of *Myzus persicae* at 10% concentration at 24, 48 and 72h exposure, respectively [55]. Consequently, [59] in a study, predicted that crude extracts from fresh leaves of *P. hysterophorus* showed significant effects against *Spodoptera littura* and *Spodoptera littorallis* with LC_{50} values as 7.96% and 8.14%, respectively.

Gas Chromatographic- Mass Spectrometric (GC-MS) analyses offered the existence of various phytochemicals in different parts of the plants. In a systematic chemical study Wang and Gan [60] Wang, Gan (60), reported that solvent fraction of *P. hysterophorus*, contained fourteen chemical compounds were plant phytosterols, stigmasterol, daucosterol, dtigmasterol-3-O-glucopyranoside, and Stigmasteryl-3β-arachidate. The other six isolated terpenoids were parthenin, oleanolic acid, oleanolic acid 28-O-β-D-glucopyranoside, and dichrocepholide D. Moreover, Białoń, Krzyśko-Łupicka (61) assessed the chemical composition of Lavender oil and found various types of terpenoids (monoterpene, monoterpenoids, sesquiterpene and sesquiterenoids) with valuable anti-bacterial as well as anti-fungal activities. Likewise, analytical aspects of *Satureja montana* and tincture were evaluated by chromatographic techniques (HPTLC) which showed its richness in polyphenols, phenolic and flavonoids contents could indicates its valuable anti-microbial and

antioxidant activities and also provides basis for further clinical studies [62]. However, for unknown compounds which are likely to be found in the extract/essential oil, it is simpler to start with the GC-MS, associated with (NIST) library will help to identify at least a part of compounds which are composing the extract and then it would be easier to separate and investigate the still unidentified compounds. Moreover, in a study, isolation and determination of phenolic compounds from fresh water algae *Cladofora glomerata* by using Clourimetric method, chromatographic and HPLC techniques and identified nine phenolic compounds along with phenolic acids and flavonoids [63].

Parthenin a chief component was found in profusion in leaf extracts of *P. hysterophorus* which was positively correlated with cytotoxicity but not with phytotoxicity. Bajwa and Weston [64] ~~Bajwa, Weston (64)~~ perceived two compounds like chlorogenic acid and ambrosin were positively correlated with germination inhibition however, among identified phytochemicals vatirenene was present in high concentration as 19.8% in root. In a study Koo and Thomas [65] Koo, Thomas (65) reported the existence of vatirenene in breath test which may be the possible cause of aspergillosis. Moreover, caryophyllene oxide is considered as an effective fungicidal mediator [66], and creation of resistance in reproductive parts [67, 68]. Likewise, globulol displays strong antibacterial activity [69]. However, the chemical compounds assessed during our current investigations from flower's extract of *P. hysterophorus* responsible towards antioxidant and insecticidal activity.

Although different studies has been conducted on phytochemistery, pharmacology as well as other biological activities but, studies on antioxidant activity, insecticidal activity against *B. brassicae* and chemical analysis of flower's extract is limited so for. The increasing interest for food, medical and agrochemical industries and to

develop botanical based natural antioxidants led towards ne investigations. Thus, this research represents the first investigation on antioxidant and insecticidal activity of flower's extract of *P. hysterophorus* against *B. brassicae*.

Conclusions

Parthenium hysterophorus is a noxious weed but also possessed numerous beneficial aspects as medical and agrochemical industries. The flower's extract of *P. hysterophorus* identified significant antioxidant activities with appreciable amount of phenolic and flavonoid contents. Similarly, the extract displayed prominent insecticidal activity against *Brevicoryne brassicae* a notorious sucking pest worldwide. Toxicity results displayed that *P. hysterophorus* extracts showed LC_{50} values of 1446 and 750µgmL^{-1} at 72 and 96 h exposure, respectively showed its effectiveness against *B. brassicae* . Moreover, antioxidant activity of *P. hysterophorus* extracts displayed strong correlation versus phenols, flavonoids and extract yield. Chemical analysis revealed the existence of various chemical compounds accountable for biological activities. Therefore, extract of *P. hysterophorus* might be used as antioxidant and insecticidal purposes as an alternative to synthetic chemical agents. However, more detailed study is required on extraction and purification of bioactive compounds and their evaluation as biological perspectives.

Acknowledgements

The support and supervision provided by Professor Zumin Gu, Ji Mingshan and experimental guidelines of lab mates of Biopesticides Laboratory, Plant Protection College Shenyang is greatly acknowledged.

Funding

This research was funded by National Key Research & Development Program of China (Grant No. (0201300)

Conflicts of Interest: "The authors declare no conflict of interest."

Author Contributions:

Conceptualization, M.J. and Z.G.; methodology, M.A.; T.A.; A.J., S; A.S. and F.A.; software, X.B. and T.u.H; validation, Z.G. formal analysis, M.J., Y.L. and Z.G.; investigation, M.J. and Z.G; resources, Z.G.; data curation, M.A. Writing—original draft, M.A.; writing—review & editing, M.J. visualization, Z.G., supervision, Z.G. and M.J.; funding acquisition, Z.G.;

All authors have read and agreed to the published version of the manuscript

References

1. Sukanya S, Sudisha J, Hariprasad P, Niranjana S, Prakash H, Fathima S. Antimicrobial activity of leaf extracts of Indian medicinal plants against clinical and phytopathogenic bacteria. Afr J. Biotechnol. 2009;8(23).

2. Adkins SW, Shabbir A, Dhileepan K. Parthenium weed: biology, ecology and management: CABI; 2018.

3. Adkins S, Shabbir A. Biology, ecology and management of the invasive parthenium weed (Parthenium hysterophorus L.). Pest Manage Sci. 2014;70(7):1023-9.

4. Ray P, Gour HN. Puja Ray and H.N. Gour (2012). Integrated Management of *Parthenium hysterophorus* L. (Asteraceae): A weed of worldwide significance. Ind J Mycol Pl Path. 2012;5:605–32.

5. Kapoor RT. Awareness related survey of an invasive alien weed, *Parthenium hysterophorus* L. in Gautam Budh Nagar district, Uttar Pradesh, India. J Agric Technol. 2012;8(3):1129-40.

6. Khan N, Bibi K, Ullah R. Distribution pattern and ecological determinants of an invasive plant *Parthenium hysterophorus* L., in Malakand division of Pakistan. J Mt Sci. 2020;17:1670-83.

7. Kushwaha VB, Maurya S. Biological utilities of Parthenium hysterophorus. J Appl Nat Sci. 2012;4(1):137-43.

8. Kaur M, Aggarwal NK, Kumar V, Dhiman R. Effects and management of Parthenium hysterophorus: A weed of global significance. Int Sch Res Notices. 2014;2014.

9. Javaid A, Jabeen T, Khan IH, Jabeen K, Akbar M. Herbicidal potential of *Alternaria citri* Ellis and Pierce metabolites against *Parthenium hysterophorus*. L. Allelopathy J. 2022;55(1):25-34.

10. Khan IH, Javaid A, Ahmad S. Metabolites of *Penicillium citrinum* as potent herbicides against parthenium weed. Pak J Weed Sci Res. 2021;28(1):77-85.

11. Raza S, Chaudhary A, Mumtaz M, Bashir S. Antioxidant, iron chelating, lipase inhibition activities and metabolite's prediction of hyrdoethanolic leaf extract of Conocarpus erectus. Food Res. 2019;4:482-7.

12. Seufi AM, Mohammed HA, Moussa FI, El-Din AAS, El-Saadani M, Taha TH, et al. Protective Effects of the Aqueous Extract of Black Mulberry Leaves, Morus nigra, on Chlorpyrifos Toxicity in Male Albino Rats. Jordan J Biol Sci. 2019;12(4).

13. Kapoor RT. Farmers'perception about *Parthenium hysterophorus* l.-a case study of handia and phulpur tehsils of allahabad district, uttar pradesh. 2016.

14. Picman AK, Elliott R, Towers G. Cardiac-inhibiting properties of the sesquiterpene lactone, parthenin, in the migratory grasshopper, Melanoplus sanguinipes. Can J Zool. 1981;59(2):285-92.

15. Lakshmi C, Srinivas C. Parthenium: A wide angle view. Indian J Dermatol Venereol Leprol. 2007;73(5):296.

16. Batish DR, Singh H, Kohli R, Saxena D, Kaur S. Allelopathic effects of parthenin against two weedy species, Avena fatua and Bidens pilosa. Environ Exp Bot. 2002;47(2):149-55.

17. Batish D, Singh H, Saxena D, Kohli R. Weed suppressing ability of parthenin-a sesquiterpene lactone from Parthenium hysterophorus. N Z Plant Prot. 2002;55:218-21.

18. Patel VS, Chitra V, Prasanna PL, Krishnaraju V. Hypoglycemic effect of aqueous extract of Parthenium hysterophorus L. in normal and alloxan induced diabetic rats. Indian J Pharmacol. 2008;40(4):183.

19. Selmi S, Rtibi K, Grami D, Sebai H, Marzouki L. Rosemary (Rosmarinus officinalis) essential oil components exhibit anti-hyperglycemic, anti-hyperlipidemic and antioxidant effects in experimental diabetes. Pathophysiology. 2017;24(4):297-303.

20. Asghari B, Zengin G, Bahadori MB, Abbas-Mohammadi M, Dinparast L. Amylase, glucosidase, tyrosinase, and cholinesterases inhibitory, antioxidant effects, and GC-MS analysis of wild mint (Mentha longifolia var. calliantha) essential oil: A natural remedy. Eur J Integr Med. 2018;22:44-9.

21. Borah A, Paw M, Gogoi R, Loying R, Sarma N, Munda S, et al. Chemical composition, antioxidant, anti-inflammatory, anti-microbial and in-vitro cytotoxic efficacy of essential oil of Curcuma caesia Roxb. leaves: An endangered medicinal plant of North East India. Ind Crops Prod. 2019;129:448-54.

22. Manssouri M, Znini M, Majidi L. Studies on the antioxidant activity of essential oil and various extracts of Ammodaucus leucotrichus Coss. & Dur. Fruits from Morocco. J Taibah Uni Sci. 2020;14(1):124-30.

23. Zhang C, Zhao J, Famous E, Pan S, Peng X, Tian J. Antioxidant, hepatoprotective and antifungal activities of black pepper (Piper nigrum L.) essential oil. Food Chem. 2021;346:128845.

24. Dwivedi S, Dwivedi A, Dwivedi S. Folk lore uses of some plants by the tribes of Madhya Pradesh with special reference to their conservation. Ethnobot leafl. 2008;2008(1):105.

25. Shabbir A, Javaid A. Effect of aqueous extracts of alien weed Parthenium hysterophorus and two native asteraceous species on germination and growth of mungbean, Vigna radiata L. wilczek. J Agric Res. 2010;48(4):483-8.

26. Towers G, Subba Rao P, editors. Impact of the pan-tropical weed, Parthenium hysterophorus L. on human affairs. Proceedings of the first international weed control congress; 1992: Weed Science Society of Victoria Melbourne.

27. Espinosa-Rivero J, Rendón-Huerta E, Romero I. Inhibition of Helicobacter pylori growth and its colonization factors by Parthenium hysterophorus extracts. J Ethnopharmacol. 2015;174:253-60.

28. Tanvir R, Sajid I, Hasnain S. Screening of endophytic Streptomycetes isolated from Parthenium hysterophorus L. against nosocomial pathogens. Pak J Pharm Sci. 2013;26(2):277-83.

29. Deka B, Jiten M, Bhattacharyya P. Acaricidal activities of Parthenium hysterophorus L. against red spider mite, Oligonychus coffeae Nietner (Acarina: Tetranychidae) of tea. Int J Sci Res. 2013;4(5):2319-7064.

30. Krishnaveni M. Air pollution tolerance index and antioxidant activity of Parthenium hysterophorus. J Pharm Res. 2013;7(4):296-8.

31. Nabi SA, Kasetti RB, Sirasanagandla S, Tilak TK, Kumar MVJ, Rao CA. Antidiabetic and antihyperlipidemic activity of Piper longum root aqueous extract in STZ induced diabetic rats. BMC Complement Altern Med. 2013;13(1):37.

32. Panwar R, Sharma AK, Dutt D, Pruthi V. Phenolic acids from Parthenium hysterophorus: evaluation of bioconversion potential as free radical scavengers and anticancer agents. Advances in Bioscience and Biotechnology. 2015;6(01):11.

33. Anup R, Sainath P, Padma S, Sukumaran M. In vitro cytotoxic activity of methanolic extract of Parthenium hysterophorus flowers on MCF-7 and HeLa cell lines. Int J Curr Microbiol Appl Sci. 2015;4(8):710-5.

34. Kumar S, Nair G, Singh AP, Batra S, Wahab N, Warikoo R. Evaluation of the larvicidal efficiency of stem, roots and leaves of the weed, Parthenium hysterophorus (Family: Asteraceae) against Aedes aegypti L. Asian Pac J Trop Dis. 2012;2(5):395-400.

35. Kumar VP, Chauhan NS, Padh H, Rajani M. Search for antibacterial and antifungal agents from selected Indian medicinal plants. J Ethnopharmacol. 2006;107(2):182-8.

36. Jha U, Chhajed PJ, Oswal RJ, Shelke TT. Skeletal muscle relaxant activity of methanolic extract of Parthenium hysterophorus L. leaves in swiss albino mice. Int J Pharm Life Sci. 2011;2(11).

37. Datta S, Saxena DB. Pesticidal properties of parthenin (from Parthenium hysterophorus) and related compounds. Pest Manag Sci. 2001;57(1):95-101.

38. Bharadwaja S, Singh S, Moholkar VS. Design and optimization of a sono-hybrid process for bioethanol production from Parthenium hysterophorus. J Taiwan Inst Chem Eng. 2015;51:71-8.

39. Parashar V, Parashar R, Sharma B, Pandey AC. Parthenium leaf extract mediated synthesis of silver nanoparticles: a novel approach towards weed utilization. Dig J Nanomater Biostructures. 2009;4(1).

40. Rajiv P, Rajeshwari S, Venckatesh R. Bio-Fabrication of zinc oxide nanoparticles using leaf extract of Parthenium hysterophorus L. and its size-dependent antifungal activity against plant fungal pathogens. Spectrochimica Acta Part A: Mol Biomol Spectrosc. 2013;112:384-7.

41. Yang J, Jiang F, Ma C, Rui Y, Rui M, Adeel M, et al. Alteration of crop yield and quality of wheat upon exposure to silver nanoparticles in a life cycle study. J Agric Food Chem. 2018;66(11):2589-97.

42. Ahmad MA, Yuesuo Y, Ao Q, Adeel M, Hui ZY, Javed R. Appraisal of comparative therapeutic potential of undoped and nitrogen-doped titanium dioxide nanoparticles. Molecules. 2019;24(21):3916.

43. Javed R, Ahmad MA, Gul A, Ahsan T, Cheema M. Comparison of chemically and biologically synthesized nanoparticles for the production of secondary metabolites, and growth and development of plants. Compr Anal Chem. 2021;94:303-29.

44. Ahmed M, Ji M, Qin P, Gu Z, Liu Y, Sikandar A, et al. Determination of phytochemicals, antioxidant activity and biochemical composition of chinese mugwort (*Artemisia argyi* l.) leaf extract from northeast China. Appl Ecol Environ Res. 2019;17(6):15349-62.

45. Sparkman OD. Identification of essential oil components by gas chromatography/quadrupole mass spectroscopy Robert P. Adams. J Am Soc Mass Spectrom. 2005;16(11):1902.

46. McConnachie A, Strathie L, Mersie W, Gebrehiwot L, Zewdie K, Abdurehim A, et al. Current and potential geographical distribution of the invasive plant Parthenium hysterophorus (Asteraceae) in eastern and southern Africa. Weed Res. 2011;51(1):71-84.

47. Narasimhan T, Murthy BK, Harindranath N, Rao PS. Characterization of a toxin fromParthenium hysterophorus and its mode of excretion in animals. J Bios. 1984;6(5):729-38.

48. Hasan N, Jain R. Biotoxicity of Parthenium hysterophorus extracts against Meloidogyne javanica and Helicotylenchus dihystera. Nematol Mediterr. 1984.

49. Kalaiselvi M, Subbaiya R, Selvam M. Synthesis and characterization of silver nanoparticles from leaf extract of Parthenium hysterophorus and its anti-bacterial and antioxidant activity. Int J Curr Microbiol Appl Sci. 2013;2(6):220-7.

50. Arshad M, Mumtaz MW, Chaudhary AR, Rashid U, Ali M, Mukhtar H, et al. Metabolite profiling of Cycas revoluta leaf extract and docking studies on alpha-glucosidase inhibitory molecular targets by phytochemicals. PakJ Pharm Sci. 2019;32(2 (Supplementary)):871-4.

51. Sinha SN, Paul D. Antioxidant potentials of parthenium hysterophorus L. leaf extracts. Sci Res J India. 2014;3(2):80-6.

52. Bashir S, Raza, S.A., Ahmad, M., Farhan, M., Ali, S. and William, J. Hydroethanolic leaf extract of Parthenium hysterophorus attenuates blood glucose in alloxan induced diabetic mice. Food Res 2021;5(1):72-7.

53. Ahmad J, Bagheri R, Bashir H, Baig MA, Al-Huqail A, Ibrahim MM, et al. Organ-specific phytochemical profiling and antioxidant analysis of Parthenium hysterophorus L. BioMed Res Int. 2018;2018.

54. Adeel M, Zain M, Fahad S, Rizwan M, Ameen A, Yi H, et al. Natural and synthetic estrogens in leafy vegetable and their risk associated to human health. Environ Sci Pollut Res. 2018;25(36):36712-23.

55. Rajiv P, Rajeshwari S, Yadav RH, Rajendran V. Vermiremediation: Detoxification of Parthenin toxin from Parthenium weeds. J Hazard Mater. 2013;262:489-95.

56. Tesfu F, Emana G. Evaluation of Parthenium hysterophorus L. powder against Callosobruchus chinensis L.(Coleoptera: Bruchidae) on chickpea under laboratory conditions. Afri J Agri Res. 2013;8(44):5405-10.

57. Shaheen FA, Khaliq A. Management of Pulse Beetle, Callosobruchus chinensis L.(Coleoptera: Bruchidae) in stored Chickpea using ashes, red soil powder and turpentine oil. Pak Entomol. 2005;27(2):19-24.

58. Yadav SK, Patel S. Insecticidal and repellent activity of some plant extracts against Myzus persicae (Sulzer) and Brevicoryne brassicae(Linnaeus). J Entomol Zool Stud. 2017;5(2):1434-143.

59. Wasu Yogesh H GD, Patel Sagar B. Larvicidal effects of *Parthenium hysterophorus* against polyphagus pests *Spodoptera Litura* and *Spodoptera littoralis*. Indian J Appl Res. 2019;9(2):1-19.

60. Wang M-H, Gan J-J, Chen W-H, Guan Y-L. Chemical Constituents of Parthenium hysterophorus. Chem Nat Comp. 2020;56:556-8.

61. Białoń M, Krzyśko-Łupicka T, Nowakowska-Bogdan E, Wieczorek PP. Chemical composition of two different lavender essential oils and their effect on facial skin microbiota. Molecules. 2019;24(18):3270.

62. Hudz N, Makowicz E, Shanaida M, Białoń M, Jasicka-Misiak I, Yezerska O, et al. Phytochemical Evaluation of Tinctures and Essential Oil Obtained from Satureja montana Herb. Molecules. 2020;25(20):4763.

63. Korzeniowska K, Łęska B, Wieczorek PP. Isolation and determination of phenolic compounds from freshwater Cladophora glomerata. Algal Res. 2020;48:101912.

64. Bajwa AA, Weston PA, Gurusinghe S, Latif S, Adkins SW, Weston LA. Toxic Potential and Metabolic Profiling of Two Australian Biotypes of the Invasive Plant Parthenium Weed (Parthenium hysterophorus L.). Toxins. 2020;12(7):447.

65. Koo S, Thomas HR, Daniels SD, Lynch RC, Fortier SM, Shea MM, et al. A breath fungal secondary metabolite signature to diagnose invasive aspergillosis. Clin Infect Dis. 2014;59(12):1733-40.

66. Yang D, Michel L, Chaumont J-P, Millet-Clerc J. Use of caryophyllene oxide as an antifungal agent in an in vitro experimental model of onychomycosis. Mycopathologia. 2000;148(2):79-82.

67. Saifullah K. Callus induction and cell suspension culture production of Catharanthus roseus for biotransformation studies of (-)-caryophyllene oxide. Pak J Bot. 2011;43(1):467-73.

68. Halim SA, Uddin R, Madura JD. Benchmarking docking and scoring protocol for the identification of potential acetylcholinesterase inhibitors. J Mol Graph Model. 2010;28(8):870-82.

69. Mulyaningsih S, Sporer F, Reichling J, Wink M. Antibacterial activity of essential oils from Eucalyptus and of selected components against multidrug-resistant bacterial pathogens. Pharm Biol. 2011;49(9):893-9.

Tables

Table 1. Physical appearance of the flower's extract of *Parthenium hysterophorus*.

Extract	Visibility	Physical Nature	Consistency
EtOH	Bluish green	Oily	Consistent
EA	Greenish	Oily	Consistent
DCM	Brownish	Oily/Gummy	Non-consistent
DW	Brownish	Oily/Gummy	Non-consistent

EtOH; (Ethanol), EA; (Ethyl Acetate), DCM; (Dichloromethane), DW; (Distilled water).

Table 2. Total Phenol, Flavonoid content and DPPH radical scavenging activity of the flower's extract of *Parthenium hysterophorus*.

Extracts	Phenol (GAE/g)	Flavonoid (QE/g)	DPPH Inhibition (%)
EtOH	27.55±1.46[c]	14.23±0.10[b]	88.53±0.11[b]
EA	47.79±0.85[b]	138.67±2.20[a]	67.21±0.19[c]
DCM	30.50±0.00[d]	15.38±0.04[c]	95.03±1.58[a]
DW	103.30±1.16[a]	4.81±0.01[c]	18.73±0.89[d]
Statics			
	S.S= 24268	S.S= 248063	S.S= 13880
	M.S=8069	M.S= 82687	M.S= 4626
	Df= 3	Df= 3	M.S= 3
	f= 2564***	*f*= 22488***	*f*= 1031***

Data in the columns is described as mean values ± standard deviations with various superscripts are significantly different according to DMRT" *P*> 0.05). S.S (Sum of square); M.S (Mean square); Df (Degree of freedom); *f* (Significance); *** (significance level).

Table 3. Correlation of the antioxidant activity of *P. hysterophorus* extract versus Phenols, Flavonoids and extract yield.

Treatments	Phenols	Flavonoids	DPPH	Yield
Phenols	1	-0.16	-0.99**	-0.11
Flavonoids	-0.16	1	-0.71	-0.90
DPPH	-0.99**	-0.71	1	-0.10
Yield	-0.11	-0.09	-0.10	1

Note: * Correlation is significant at 0.05 levels; ** Correlation is highly significant at 0.05 levels

Table 4. Toxicity of *P. hysterophorus* extracts against *B. brassicae* at 12, 24, 48, 72 and 96 hours exposure

Time	LC_{50} µgmL^{-1}	95% CF.L		Slope± S.E	χ2
		Lower	Upper		
12	3598	1421	35512	1.09±0.38	0.25
24	2569	1321	27758	1.51±0.46	0.99
48	2345	1153	25517	1.31±0.53	0.85
72	1446	852	21721	1.56±0.63	0.22
96	750	468	1276	1.89±0.58	0.04

Note: LC_{50} (Lethal concentration); S.E. (standard error); χ2 (chi-square); F.L. (Fiducial limit)

Table 5. Chemical composition of the flower's extract of *Parthenium hysterophorus*.

Peak #	R. T	Area %	Chemical Compounds	M.F	M.W g/mol
1	3.22	7.05	Benzene, 1,3-dimethyl-	C_8H_{10}	106.17
2	3.92	3.47	2,4-Dimethylamphetamine	$C_{11}H_{17}N$	163.26
3	4.43	2.04	3-Piperidinol	$C_5H_{11}NO$	101.15
4	5.05	2.74	2-Pentene, 4,4-dimethyl-, (E)-	C_7H_{14}	98.186
5	5.52	3.55	2-Butanamine	C_6H_5N	101.19
6	12.96	25.93	2-Naphthalenemethanol,decahydro.alpha.,.α.,4a-trimethyl-8-methylene-,[2R (2 α.,4a.α.,8a.beta.)]-	$C_{15}H_{26}O$	222.36
7	14.29	11.27	3-Methoxy-3,4-dimethyl-1-heptyne	$C_{10}H_{18}O$	154.25
8	15.21	4.76	Undecane	$C_{11}H_{24}$	156.31
9	15.85	3.82	2-Pyrrolidinone, 1-methyl-	C_5H_9NO	99.13
10	16.62	7.26	Glycinamide hydrochloride	$C_2H_7C_lN_2O$	110.54
11	17.58	2.18	1,2-Benzenedicarboxylic acid, butyl 2-ethylhexyl ester	$C_{20}H_{30}O_4$	334.45
12	18.1	6.67	Hexadecanoic acid, ethyl ester	$C_{18}H_{36}O_2$	284.48
13	21.85	19.25	Histidine, 1,N-dimethyl-4-nitro-	$C_8H_{10}N_2O_2$	166.18

R.T (Retention time); M.F (Molecular formula); M.W (Molecular Weight).

Original Manuscript

Antioxidant, Insecticidal Activity and Chemical Profiling of Flower's Extract of ~~Carrot Grass~~Parthenium weed (*Parthenium hysterophorus* L.)

Maqsood Ahmed[1,2], Ansar Javeed[3], Samiullah[2], Talfoor-ul-Hassan[4], Aatika Sikandar[1], Taswar Ahsan[5], Faqir Ahmed[6], Ji Mingshan[1], Xuejing Bai[7], Ying Li[8] and Zumin Gu[1,*]

[1]College of Plant Protection, Shenyang Agricultural University, Shenyang, 110866, P.R. China. aatika_sikander@yahoo.com (A.S.); jimingshan@syau.edu.cn (M.J.)
[2]Department of Agriculture (Plant Protection), Pest Warning and Quality Control of Pesticides, Gujrat, 50700, Pakistan. maqsoodahmed200@hotmail.com
[3]Henan University, Jinming Campus, Kaifeng City Henan China. ansarjaveed@henu.edu.cn (A.J)
[4]Department of Agriculture (Plant Protection), Pest Warning and Quality Control of Pesticides, Gujranwala, 52250, Pakistan. ddappgujranwala@gmail.com (T.u.H.)
[5]Institute of Plant Protection Liaoning Academy of Agricultural Sciences, Shenyang, 110866, P.R. China. taswarahsan@163.com (T.H)
[6]Directorate General of Agriculture, Pest Warning and Quality Control of Pesticides, Lahore, 54800, Pakistan. faqirahmad63@yahoo.com (F.A)
[7]Shenyang Academy of Landscape- Gardening Shenyang 110016, P.R. China. baixuejingnv@163.com (X.B.)
[8] Fushun Modern Agriculture and Poverty Alleviation Development Promotion Centre, Fushun 113006 P.R. China. liying_1973@163.com (Y.L.)

Corresponding author: *guzumin1212@163.com *(G.Z); Tel: +86-24- 8848- 7148; Fax: +86-24-8834-2315*

Abstract

Parthenium hysterophorus L. an invasive alien species is considered a noxious weed and confers numerous benefits in medical and agrochemical industries. The present study evaluated the flower extract's antioxidant and insecticidal activity and chemical profiling to appraise the existence of phytoconstituents responsible for biological activities. Assay was performed by 1,1-diphenyl-2-picrylhydrazyl (DPPH) for antioxidant activity, whereas, chemical configuration was evaluated by Gas Chromatography Mass Spectrometry (GCMS) analysis. Our findings report that the extract of *P. hysterophorus* possesses potential radical scavenging activity (95.03%) when extracted via dichloromethane (DCM). Additionally, phytochemical analysis revealed a considerable amount of phenols and flavonoid contents when extracted via distilled water and ethyl acetate; (103.30 $GAEg^{-1}$ and 138.67 $QE\text{-}g^{-1}$) respectively. Maximum mortality 63.33% and 46.67% was observed at 1000 μgmL^{-1} and 800 μgmL^{-1} after 96 h exposure followed by 46.67% and 36.67% at 72 h at the same concentration. On the other hand, toxicity results displayed that *P. hysterophorus* extracts showed LC_{50} values of 1446 and 750 μgmL^{-1} at 72 and 96 h exposure, respectively. However, Imidacloprid being a positive control afforded maximum mortality at 96 h and 72 h exposure 97.67% and 91.82%, respectively. Moreover, antioxidant activity of *P. hysterophorus* extracts displayed strong correlation versus phenols, flavonoids and extract yield. GCMS analysis revealed the existence of 13 chemical compounds corresponding to 99.99% of whole extract. Moreover, maximum extract was afforded by Ethanol followed by distilled water, ethyl acetate and dichloromethane as 4.34, 3.22, 3.17 and 2.39%, respectively. *P. hysterophorus* flower's extract therefore showed good antioxidant and aphicidal activity which also exhibited valuable chemical compounds accountable for biological activity, making them suitable candidates for an alternative to synthetic agents. However, our results provide novel and fundamental basis for further exploration on purification of chemical compounds for their biological activities.

Keywords: *Parthenium hysterophorus*; phytochemicals; Antioxidant activity; GCMS; DPPH

Introduction

The invasive alien species exert devastating impacts on inherent biota and deleteriously affecting ecosystems by destructions of native species. Likewise, such distressing impacts on natural biodiversity could lead towards escalation of climate change, environmental pollution, and ecological destruction of natural habitat. However, identifying the beneficial and harmful impacts of *Parthenium hysterophorus* introduced as an invasive alien species, might lead towards control of biological invasions. Invasive alien plant species are the worldwide risk to biodiversity, functioning of ecosystem and farm production [1]. Invasive weeds species are undesirable self-grown plants that compete with the cultivated and non-cultivated crops for nutrients, sunlight and water. They also produce a wide range of bioactive particles that are rich sources of various types of medicines. Currently, most of the medications in the pharmaceutical industry depend upon natural plants and or in the herbal traditional systems [1].

Parthenium hysterophorus L. is an extremely invasive plant species belonging to family Asteraceae that has invaded almost fifty countries in sub-continent Africa, Asia, and Oceania, infesting forests, feeding lands, and urban landscapes [2, 3]. It is a devastating and hazardous weed of various economically important crops [4]. Moreover, it is amongst the top ten worst weeds in the world and is included in the world catalogue of destructive species [5, 6]. In Europe *P. hysterophorus* is considered as an ephemeral species. It is generally recognized by different names such as ~~carrot grass~~parthenium weed, congress grass, Gajjar botti, white head and white top [7]. It is native to Gulf of Mexico and United States. However, it accidently introduced in Indian subcontinent [8]. This weed has been considered as a major weed

of high rank spreading at a much faster pace to takeover abandoned land, canal, watercourse banks, roads, and fields to challenge crop productivity [9, 10].

Medicinal plants are the fundamentals of traditional therapeutic systems based on the trusts and observations comprised of hundreds of years [11]. The phytochemical constituents such as phenolic and flavonoid are secondary metabolites involved in therapeutic system, because of the existence of these functional phytochemicals mainly parthenin [12]. Moreover, *P. hysterophorus* renowned to contain vital biochemical compounds such as alkaloids, tannins, and saponins, which are important from the medicinal viewpoint [13]. It also exhibited allelopathic effects and important phytochemicals such as parthenon and coronopilin which are auto-toxic to its own seed germination and seedling growth [14]. Furthermore, serious health consequences on human beings and livestock have been reported [15]. The agriculturists who are come in contact with *P. hysterophorus*; the pollen and dust produced by this weed provoke allergic contact dermatitis in human beings. Major chemical compounds, parthenin's contained by *P. hysterophorus* exhibited phytotoxic properties against a variety of plants, including weeds and various crops such as Slender amaranth (*Amaranthus viridis*), Billygoat weed (*Ageratum conyzoides*), common wild oat. (*Avena fatua*), nettleleaf goosefoot (*Chenopodium murale*), Foetid cassia (*Cassia tora*), mung bean *Phaseolus aureus* [16, 17]. Moreover, parthenin's the main biochemical compounds showed insecticidal activity against termites, cockroaches and migratory grasshoppers, *Melanoplus sanguinipes* [7]. Similarly, Patel, Chitra [18] reported pesticidal activity of the ethanol extract of *P. hysterophorus* which showed substantial lethal effect against stored grain pest like *Tribolium* sp. and *Oryzaephilus* sp. Essential oils from the natural plants also have antioxidant effects. In recent years several reports have been breakout, the essential oil from Rosemary [19], wild mint

(*Mentha longifolia* var. *Calliantha*) [20], black turmeric (*Curcuma caesia*) [21] *Ammodaucus leucotrichus* [22] and black pepper (*Piper nigrum*) [23].

Pharmacologically, it is involved in treating numerous diseases like allergies, anemia, sores, skin diseases, facial neuralgia, fever, vermifuge, blood purifier, and insecticide [7, 24]. This plant species is enriched with vital phytoconstituents however; sesquiterpene lactones are the most copious phytochemicals exhibited by this plant which are accountable for injurious effects such as strong allelopathic [25], toxic to livestock [26], It also possess many advantageous effects like antibacterial [27, 28], acaricidal [29], antioxidant [30, 31], cytotoxic [32, 33], larvicidal potential against *Aedes aegypti* L. [34, 35], skeletal muscle relaxant [36], pesticidal agent [37], producing bioethanol [38], as reducing agent in the formation of silver nanoparticles [39] and in production of zinc nanoparticles [40]. However, various factors affect the biological profile of the plants and from some recent studies it has been reported that nanoparticle influenced the physiological and biological properties of the plants. It has been reported that metal-based nanoparticle showed a decrease in protein and amino acid and micronutrient contents in wheat crop grown under such conditions [41]. Moreover, nitrogen doped titanium dioxide nanoparticle enhanced antioxidant activity via reducing power assay and percent inhibition of DPPH against *Streptomyces* [42]. Likewise, synthetically and biologically prepared nanoparticle significantly exerted effects on plant growth and secondary metabolites which ultimately triggered the biological activity of the plants [43].

As some earlier researches have been conducted on this plant species but, detailed studies on chemical and quantitative phytochemical analysis and assessment of antioxidant activities from different solvents extract as well as aphicidal activity against cabbage aphid *B. brassicae* from flower’s extracts has not been explored

before. Hence, keeping in view the diverse biological properties of *P. hysterophorus*, current study was designed to determine the antioxidant and insecticidal activity of flower's extract against *B. brassicae* with appraisal of quantitative phytochemical analysis as well as assessment of chemical profiling of extract.

Materials and Methods

Collection and Samples Preparation

The whole plant samples were collected from District Sialkot Punjab Pakistan during the month of June-July 2019 and were identified as *Parthenium hysterophorus* L., from Ayub Agricultural Research Institute (AARI) Faisalabad Pakistan by Dr. Dilbar Hussain. Flowers were manually separated from the plants and left over for 20 days to dry under shade. These dried flowers were powdered by electric grinder and then extracted with different solvents (ethanol, ethyl acetate, dichloromethane and distilled water) by cold extraction method for seventy two hours at fixed temperature 28°C and at 100 rpm in an incubator shaker (ZWY-1102C). Extracted contents were filtered and concentrated to lessen the volume on rotary evaporator model R-210 BUCHI Labortechnik AG, CH-9230 Flawil 1/Switzerland). Obtained extract's yield was measured using following Equation (Eq. 1).

$$\text{Yield (\%)} = \frac{\text{Weight of the extract}}{\text{Weight of the dried sample}} \times 100 \qquad \text{(Eq. 1)}$$

Quantitative Analysis for Total Phenolic and Flavonoids Content

To assess the phytochemicals quantitatively, 1 mg of extract from each solvent was dissolved in 1 mL Methanol separately. Consequent mixture was vortexed and separated for analysis. For the assessment of total content for phenols Folin–Ciocalteu reagent test was used. In brief, 1 mL solution (1 $mgmL^{-1}$) was added to 2.5 mL of (10%) Folin–Ciocalteu with supplementary addition of 2 mL solution of (2%) sodium carbonate (Na_2CO_3). The consequential blend was incubated for fifteen min in the

dark to measure absorbance at 765 nm in 96-well ELISA plate using (SpectraMax 190, Meigu molecular International Co. Ltd., Shanghai China). To build a standard curve, Gallic acid was used (1 $mgmL^{-1}$) at different concentrations such as 1, 0.50, 0.25, 0.10, 0.05, 0.02, 0.01, 0 mg mL^{-1} to describe the obtained results as Gallic acid equivalent (GAE) mg g^{-1} of total extract. Each treatment was replicated ten times to analyze the data.

To examine the total flavonoids content, aluminium chloride colourimetric method was adopted. Briefly, 1 mL solution (1$mgmL^{-1}$) was poured into 3 mL methanol, with addition of 0.2 mL of 1 M Potassium acetate (CH_3COOK), 0.2 mL of (10%) aluminium chloride ($AlCl_3$) and at the end, distilled water 5.6 mL was added to the consequent mixture. The incubation of solution was performed for half an hour in the darkness and absorbance at 420 nm was measured. A standard curve was built using quercetin at different concentrations such as 1, 0.50, 0.25, 0.10, 0.05, 0.02, 0.01, 0 $mgmL^{-1}$ to represent the results as quercetin equivalent (QE) mg g^{-1} of total extract. The procedure was replicated 10 times for each treatment.

Assessment of Free Radical Scavenging Activity

Antioxidant activity of extracts obtained by different solvents from *Parthenium hysterophorus* flowers was accomplished in 1% solution of tween 20 by using 1,1-diphenyl-2-picrylhydrazyl (DPPH) ($C_{18}H_{13}N_5O_6$) a free radical. In brief, to assess the free radical scavenging activity, all dried solvent extracts were mixed together in equal proportion (5 mg each) and were dissolved in HPLC grade methanol and filtered. The filtrate was formerly used to assess the antioxidant activity. Similarly, DPPH solution was prepared in same grade Methanol @ 0.001g 25 mL^{-1}. Then, into 3.5 mL freshly prepared DPPH solution and 0.25 mL extract prepared in Methanol was added. The consequent mixture was shacked for complete mixing and then the

resulting mixture was incubated in darkness at 28°C for 30 min [44]. After incubation of resulting mixture for stipulated period of time, the absorbance was assessed at 517nm by using (SpectraMax 190, Meigu molecular International Co. Ltd., Shanghai China). The inhibition percentage of primed DPPH solution was predicted on reduction of absorbance through following Equation (Eq. 2). Each treatment was replicated ten times to analyze the data.

$$\text{Inhibition (\%)} = \frac{A_{\text{blank}} - A_{\text{sample}}}{A_{\text{blank}}} \times 100 \qquad \text{(Eq. 2)}$$

Where: A_{blank} = (control absorbance); A_{Sample} = (samples absorbance).

Correlation of Antioxidant Activity versus Phenols, Flavonoids and extract yield

Correlation of antioxidant activity *P. hysterophorus* versus Phenols, Flavonoids and extract yield was carried out using SPSS statistics 25.0 version with significant values at (P>0.05) level.

Assessment of Insecticidal Activity

Residual toxicity method was adopted for the assessment of insecticidal activity of extract of *P. hysterophorus* flowers. Samples for bioassay study were prepared to obtain mother mixture by mixing 0.1g of each solvent's extract and dissolved in Acetone, because it is aprotic solvent having intermediate polarity comparable to polar parotic solvents and it also evaporates immediately from the extract when exposed to air. The resulting mixture was placed in the fume hood for six hours for complete drying of Acetone. Serial concentrations *viz*. 100, 200, 400, 800 and 1000 µg mL^{-1} was prepared in Tween-20 (1%) solution from mother mixture. Briefly, fresh cabbage leaf discs 5cm in diameter were cut off and dipped for 10 s in the respective concentration and then dried for 5 min at room temperature. Formerly, 10 adult wingless aphids were carefully released onto the leaf disc using a fine-haired brush contained in the Petri dish and incubated for 96 h at 65% R.H., 25°C and with a 16:8

(light: dark) photoperiod. Imidacloprid 25% WP was used as the positive control at a rate of 0.0025 mL mL^{-1} of water, and control (CK) with a 1% Tween 20 solution. Positive controls and CKs were placed in a greenhouse for 96 h. Mortality data was collected at 12, 24, 48, 72 and 96 h using binocular microscope and the response of aphids was observed by needle probing, and the aphids who offered no response on stimulation were considered dead. Each treatment was replicated five times.

Chemical Analysis

Samples for analysis were prepared by mixing 0.1 g of each solvent extract into 2 mL of HPLC grade methanol to obtain mother solution. Consequent mixture was vortexed for 1 min following the addition of graphitized carbon black “GCB” (0.005 g). Mixture was centrifuged for 10 min at 10000 rpm at 25°C and colorless material was separated and collected in (1.5 mL) centrifuges tubes. Work for GC-MS analysis was accomplished using an Agilent Model 6890-5973N accompanied with gas chromatograph established on HP1 capillary column TG-5MS polydimethylsiloxane (length 30m × diameter 250 µm × film thickness 0.25 µm) interfaced with mass selective detector Hewlett Packard (5973N). The established parameters were; preliminary temperature was adjusted at 70°C for 0 min however, final temperature was extended to 200 °C for 10°C min^{-1} whereas, inlet temperature was adjusted at 250°C with split ratio 10:1. MS quadruple temperatures were fixed at 150°C while thermal aux temperatures were 285°C. The Scan range for MS was 35–520 units however; as a carrier, helium gas was used bearing stream frequency of 1.0mLmin^{-1}. The existing compounds in the extract were identified by comparison of their retention time and the mass spectra of the NIST 08 and Wiley 7 of the data base and confirmed by comparing with GC-MS literature data at NIST and TMS database Wiley/NIST.1998.1 [45].

Statistical Analysis

All calculated data was analysed by one way analysis of variance (ANOVA), mean difference between treatments was calculated for significance test by Duncan Multiple Range Test “DMRT ” at $P > 0.05$) with IBM-SPSS statistics 25.0 version software. Probit analysis was performed using EPA Probit analysis program version 1.5.

Results

The current study carried out on the flower’s extract of *Parthenium hysterophorus* revealed the existence of medicinally and agro-chemically active compounds. The phytochemical constituents of *P. hysterophorus* were qualitatively analyzed for antioxidant and insecticidal activities.

Extract Yield (%)

Yield of extract by the solvent extraction technique from *P. hysterophorus* flowers was calculated and presented in (Figure 1). The maximum extract was obtained by ethanol (4.34%) whereas; minimum extract quantity (2.39%) was afforded by dichloromethane. The extract obtained via ethanol and ethyl acetate was bluish green and greenish in color and exhibited consistency, respectively. The same oil was brownish in color and non-consistent via dichloromethane and distilled water, respectively, (Table 1).

Analysis for Phenol and Flavonoid and Antioxidant Activity

The total contents for phenol and flavonoid as well as DPPH scavenging activity afforded by *P. hysterophorus* flower’s extract via different solvents are presented in (Table 2). Results confirmed that the highest total phenol were reported from distilled water 103.30 GAE/g followed by Ethyl acetate and Ethanol’ extract, 47.79 and 27.55 GAE/g, respectively. On the other hand highest flavonoid was reported from Ethyl acetate extract 138.67 QE/g followed by Ethanol 14.23 QE/g respectively.

Inhibition (%) was determined by 1,1-Diphenyl-1-picrylhydrazyl (DPPH) displayed color absorption at 517nm on using spectrophotometer. When DPPH trap free radicals, the color of the solution changed to light yellow and, subsequently, resulted in decrease of the absorbance. Maximum DPPH radical scavenging activity was afforded by Dichloromethane extract 95.03% followed by Ethanol and Ethyl acetate 88.53% and 67.21%, respectively.

Correlation of Antioxidant Activity versus Phenols, Flavonoids and extract yield

In our study, the Pearson's correlation regarding antioxidant activity of *P. hysterophorus* showed positive relationship (Table 3). Results showed highly significant ($P<0.01$) negative effect of phenol with DPPH as well as DPPH showed strongly highly significant negatively correlation and similar results were found reciprocally in the studied parameters. However, yield extracted by different solvents did not affect the Antioxidant Activity versus phenols, flavonoids quantitatively.

Assessment of Insecticidal Activity

The mortality data of cabbage aphid *B. brassicae* at specific period of time using *P. hysterophorus* flower's extract is presented in (Figure 2). It was perceived that the percent mortality was directly correlated to the concentration and the time. The results demonstrated that the maximum mortality was recorded at 1000 μgmL^{-1} at 96 h and 72 h with 63.33% and 46.67%, respectively. Same trend of mortality was also observed at 800 μgmL^{-1} with 56.67% and 36.67%, respectively. Imidacloprid 25% WP being a positive control produced the highest rates of mortality after 96 h of exposure 97.67%. Likewise, Imidacloprid also produced significant mortality after 72 h and 48 h exposure 91.82% and 87.38% mortality, respectively.

Analysis for probability of the data showed the LC_{50} values, slope, chi-square, and fiducial limits at confidence interval of 95% for *P. hysterophorus* extracts. Mortality response using plant extracts showed sensitivity of *B. brassicae* to various concentrations which is represented in (Table 4).

Chemical Analysis

The existence of chemical compounds in *P. hysterophorus* flower's extract was analyzed by GCMS techniques and data is presented in (Table 5). Thirteen biochemical compounds equivalent to 99.99% of entire extract by GC fraction were identified. Moreover,2-naphthalenemethanol,decahydro-.alpha.,.alpha.,4a-trimethyl-8-methylene-, [2R--(2.alpha., 4a.alpha.,8a.beta.)]-(25.93%), histidine, 1,N-dimethyl-4-nitro- (19.25%) and 3-methoxy-3, 4-dimethyl-1-heptyne (11.27%), were the major chemical compounds while, 10 others chemical compounds were identified in minor amounts having areas with peak range 2.04–3.7%. GC-MS chromatogram of *P. hysterophorus* flower extract is presented in (Figure 3).

Discussion

The chemical compounds include phenolic, steroid, alkaloids, flavonoids and terpenoids depends their concentration on ecological conditions and occurrence of the pathogens. They also possessed bio-pesticidal properties and are target specific against insect pests and pathogens. Moreover, these compounds are biodegradable, without triggering severe harm to the concerns of the environment [27, 46-48].

Appraisal of antioxidant and polyphenols activity of medicinal products has become vital device to recognize the medicinal possessions of natural plants. An antioxidant is a molecule which exhibited the ability to inhibit the oxidation of other chemical elements. Kumar and Mishra [44] reported that alkaloids, flavonoids, terpenoids, and cardiac glycosides from the extract of *P. hysterophorus*. However, the

phenolic contents in flower extracts were ranging from 86.69-320.17 mg propyl gallate equivalent (PGE)/g. Study established that bio-chemicals possessed by *P. hysterophorus* extracts bears significant antioxidant activity consistent to our current findings. Similarly, 50% radical scavenging potential of *P. hysterophorus* extract at 250 μgmL^{-1} using DPPH assay as well as 30% inhibition at 100 μgmL^{-1} via hydrogen per oxide assays was reported from *P. hysterophorus* leaves extract [49]. Antioxidant activities of hydro-ethanolic extracts of *P. hysterophorus* leaves was perhaps because of the existence of polyphenols contents [11, 50].

Likewise, Sinha and Paul [51] found that extract from *P. hysterophorus* possess valuable antioxidant activity with 54.5% scavenging property and 60.2 IC_{50} via DPPH assay while, 93.2% and 33.6 IC_{50} via ABTS assay which are consistent with our findings on antioxidant activity. Bashir [52] evaluated leaf extract of *P. hysterophorus* for the presence of total phenolic and flavonoid contents as 105.44mg GAE/g and 41.50mg RE/g respectively exhibited by 60% ethanolic extract with IC_{50} value of 87.55 μgmL^{-1} and 98.22 μgmL^{-1} for DPPH radical scavenging activity respectively supported our investigations on antioxidant as well as phenol and flavonoid contents from *P. hysterophorus*. Phytochemical profile of *P. hysterophorus* root, stem, leaf and phyllary identified terpenes, fatty acids, hydrocarbons and phytosterols however; root extracts showed highest antioxidant potential [53]. The results obtained on antioxidant activity of *P. hysterophorus* are in agreement with Sinha and Paul [51] who reported that its leaves crude extract bears considerable antioxidant activity being IC^{50} 60.24 and 33.60% by DPPH and ABTS method respectively.

As it is evident from previous findings that exposure of natural fauna to harmful chemicals or particle can altered the biological activities, consequently, sometimes estrogen compounds cause acute toxicity in edible crops and vegetables which may

occur via long-term application of estrogen-containing irrigation water. Comparable results were reported by Adeel, Zain [54] that uptake and accumulation of l7β-estradiol (17β-E2) and ethinylestradiol (EE2) in lettuce (*Lactua sativa*) grown under lab conditions.

The historic practice of using nicotine and pyrethrum for pest control measures has encouraged scientists to draw their attention to secondary metabolites like terpenoids, alkaloids, and flavonoids as pest regulator agents [55]. So, the outcomes of the current exploration are in agreement with other workers like Tesfu and Emana [56] ~~who~~ evaluated different parts of *P. hysterophorus* extract against *Callosobruchus chinensis* and establish that 73.33% mortality was caused after 96 h exposure followed by 76.67% and 56.67% via inflorescence powder and stem powder respectively. Moreover, their leaves extract afforded repellent affects as 70–100% for oviposition performance of the *Aedes aegypti* adults. However, the diethyl ether extracts resulting in extreme repellency as 99.7%.

The insecticidal impact of plant extracts may attribute to the following belongings including repellency and stomach poisoning might decrease insect movement and also cause death through constriction of the insect spiracles [57]. A study reported the chemical examination of parthenium has specified that whole plant exhibited toxins called as sesquiterpene lactones responsible for insecticidal activities however; the major constituent of these toxins is parthenin derivatives. These derivatives showed insecticidal as well as nematicidal activity against the *Callosobruchus maculatus* adults and root knot nematode *Meloidogyne incognita*. Similarly, the pyrazoline was found highly effective being an insecticide, with LC_{50} value 32 mgl^{-1} with exposure of 72 h, and found extremely effective as nematicidal activity with LC_{50} values being 512 mgl^{-1} perceived for parthenin at 72 h exposure [37].

Parthenium *hysterophorus* extract used against mustard aphid, *Lipaphis erysimi* under field conditions exhibited incredible decrease of 29% of the infestation which may be because of the effect of phenolic acids [58]. Ahmad, Bagheri [53] reported the efficacy of *P. hysterophorus* extract with 10 and 20% concentration found 26.70% and 27% mortality at 21 day after treatment. Consequently, Wasu Yogesh H [59] in their study reported the activity of leaves and stem extract of *P. hysterophorus* against different larval instars of *Aedes aegypti*. Stored stock and freshly prepared solution caused 80% and 100% mortality, respectively with concentration prepared in 200g/500 ml. Another study reported that crude aqueous extracts of whole plant of *P. hysterophorus* caused a significant mortality with 73.33, 86.67 and 83.33% of *B. brassicae* as well as moderate mortality being 26.67, 46.67 and 63.33% of *Myzus persicae* at 10% concentration at 24, 48 and 72h exposure, respectively [55]. Consequently, Wasu Yogesh H [59] in a study, predicted that crude extracts from fresh leaves of *P. hysterophorus* showed significant effects against *Spodoptera littura* and *Spodoptera littorallis* with LC_{50} values as 7.96% and 8.14%, respectively.

Gas Chromatographic- Mass Spectrometric (GC-MS) analyses offered the existence of various phytochemicals in different parts of the plants. In a systematic chemical study Wang, Gan [60], reported that solvent fraction of *P. hysterophorus*, contained fourteen chemical compounds were plant phytosterols, stigmasterol, daucosterol, dtigmasterol-3-O-glucopyranoside, and Stigmasteryl-3β-arachidate. The other six isolated terpenoids were parthenin, oleanolic acid, oleanolic acid 28-O-β-D-glucopyranoside, and dichrocepholide D. Moreover, Białoń, Krzyśko-Łupicka [61] assessed the chemical composition of Lavender oil and found various types of terpenoids (monoterpene, monoterpenoids, sesquiterpene and sesquiterenoids) with valuable anti-bacterial as well as anti-fungal activities. Likewise, analytical aspects of

Satureja montana and tincture were evaluated by chromatographic techniques (HPTLC) which showed its richness in polyphenols, phenolic and flavonoids contents could indicates its valuable anti-microbial and antioxidant activities and also provides basis for further clinical studies [62]. However, for unknown compounds which are likely to be found in the extract/essential oil, it is simpler to start with the GC-MS, associated with (NIST) library will help to identify at least a part of compounds which are composing the extract and then it would be easier to separate and investigate the still unidentified compounds. Moreover, in a study, isolation and determination of phenolic compounds from fresh water algae *Cladofora glomerata* by using Clourimetric method, chromatographic and HPLC techniques and identified nine phenolic compounds along with phenolic acids and flavonoids [63].

Parthenin a chief component was found in profusion in leaf extracts of *P. hysterophorus* which was positively correlated with cytotoxicity but not with phytotoxicity. Bajwa, Weston [64] perceived two compounds like chlorogenic acid and ambrosin were positively correlated with germination inhibition however, among identified phytochemicals vatirenene was present in high concentration as 19.8% in root. In a study Koo, Thomas [65] reported the existence of vatirenene in breath test which may be the possible cause of aspergillosis. Moreover, caryophyllene oxide is considered as an effective fungicidal mediator [66], and creation of resistance in reproductive parts [67, 68]. Likewise, globulol displays strong antibacterial activity [69]. However, the chemical compounds assessed during our current investigations from flower's extract of *P. hysterophorus* responsible towards antioxidant and insecticidal activity.

Although different studies has been conducted on phytochemistery, pharmacology as well as other biological activities but, studies on antioxidant activity, insecticidal

activity against *B. brassicae* and chemical analysis of flower's extract is limited so for. The increasing interest for food, medical and agrochemical industries and to develop botanical based natural antioxidants led towards ne investigations. Thus, this research represents the first investigation on antioxidant and insecticidal activity of flower's extract of *P. hysterophorus* against *B. brassicae*.

Conclusions

Parthenium hysterophorus is a noxious weed but also possessed numerous beneficial aspects as medical and agrochemical industries. The flower's extract of *P. hysterophorus* identified significant antioxidant activities with appreciable amount of phenolic and flavonoid contents. Similarly, the extract displayed prominent insecticidal activity against *Brevicoryne brassicae* a notorious sucking pest worldwide. Toxicity results displayed that *P. hysterophorus* extracts showed LC_{50} values of 1446 and 750µgmL^{-1} at 72 and 96 h exposure, respectively showed its effectiveness against *B. brassicae* . Moreover, antioxidant activity of *P. hysterophorus* extracts displayed strong correlation versus phenols, flavonoids and extract yield. Chemical analysis revealed the existence of various chemical compounds accountable for biological activities. Therefore, extract of *P. hysterophorus* might be used as antioxidant and insecticidal purposes as an alternative to synthetic chemical agents. However, more detailed study is required on extraction and purification of bioactive compounds and their evaluation as biological perspectives.

Acknowledgements

The support and supervision provided by Professor Zumin Gu, Ji Mingshan and experimental guidelines of lab mates of Biopesticides Laboratory, Plant Protection College Shenyang is greatly acknowledged.

Conflicts of Interest: "The authors declare no conflict of interest."

Data Availability: All relevant data are within the manuscript

Funding: This research was supported by National Key Research & Development Program of China (Grant No. 0201300). However, the funders had no role in study design, data collection and analysis, decision to publish, or preparation of the manuscript. Moreover, No any support was received by any third party in conducting this research, analyzing the data or preparing the manuscript for submission

Author Contributions: Conceptualization, M.J. and Z.G.; methodology, M.A.; T.A.; A.J., S; A.S. and F.A.; software, X.B. and T.u.H; validation, Z.G. formal analysis, M.J., Y.L. and Z.G.; investigation, M.J. and Z.G; resources, Z.G.; data curation, M.A. Writing—original draft, M.A.; writing—review & editing, M.J. visualization, Z.G., supervision, Z.G. and M.J.; funding acquisition, Z.G.

All authors have read and agreed to the published version of the manuscript

References

1. Sukanya S, Sudisha J, Hariprasad P, Niranjana S, Prakash H, Fathima S. Antimicrobial activity of leaf extracts of Indian medicinal plants against clinical and phytopathogenic bacteria. Afr J. Biotechnol. 2009;8(23).

2. Adkins SW, Shabbir A, Dhileepan K. Parthenium weed: biology, ecology and management: CABI; 2018.

3. Adkins S, Shabbir A. Biology, ecology and management of the invasive parthenium weed (Parthenium hysterophorus L.). Pest Manage Sci. 2014;70(7):1023-9.

4. Ray P, Gour HN. Puja Ray and H.N. Gour (2012). Integrated Management of *Parthenium hysterophorus* L. (Asteraceae): A weed of worldwide significance. Ind J Mycol Pl Path. 2012;5:605–32.

5. Kapoor RT. Awareness related survey of an invasive alien weed, *Parthenium hysterophorus* L. in Gautam Budh Nagar district, Uttar Pradesh, India. J Agric Technol. 2012;8(3):1129-40.

6. Khan N, Bibi K, Ullah R. Distribution pattern and ecological determinants of an invasive plant *Parthenium hysterophorus* L., in Malakand division of Pakistan. J Mt Sci. 2020;17:1670-83.

7. Kushwaha VB, Maurya S. Biological utilities of Parthenium hysterophorus. J Appl Nat Sci. 2012;4(1):137-43.

8. Kaur M, Aggarwal NK, Kumar V, Dhiman R. Effects and management of Parthenium hysterophorus: A weed of global significance. Int Sch Res Notices. 2014;2014.

9. Javaid A, Jabeen T, Khan IH, Jabeen K, Akbar M. Herbicidal potential of *Alternaria citri* Ellis and Pierce metabolites against *Parthenium hysterophorus*. L. Allelopathy J. 2022;55(1):25-34.

10. Khan IH, Javaid A, Ahmad S. Metabolites of *Penicillium citrinum* as potent herbicides against parthenium weed. Pak J Weed Sci Res. 2021;28(1):77-85.

11. Raza S, Chaudhary A, Mumtaz M, Bashir S. Antioxidant, iron chelating, lipase inhibition activities and metabolite’s prediction of hyrdoethanolic leaf extract of Conocarpus erectus. Food Res. 2019;4:482-7.

12. Seufi AM, Mohammed HA, Moussa FI, El-Din AAS, El-Saadani M, Taha TH, et al. Protective Effects of the Aqueous Extract of Black Mulberry Leaves, Morus nigra, on Chlorpyrifos Toxicity in Male Albino Rats. Jordan J Biol Sci. 2019;12(4).

13. Kapoor RT. Farmers’perception about *Parthenium hysterophorus* l.-a case study of handia and phulpur tehsils of allahabad district, uttar pradesh. 2016.

14. Picman AK, Elliott R, Towers G. Cardiac-inhibiting properties of the sesquiterpene lactone, parthenin, in the migratory grasshopper, Melanoplus sanguinipes. Can J Zool. 1981;59(2):285-92.

15. Lakshmi C, Srinivas C. Parthenium: A wide angle view. Indian J Dermatol Venereol Leprol. 2007;73(5):296.

16. Batish DR, Singh H, Kohli R, Saxena D, Kaur S. Allelopathic effects of parthenin against two weedy species, Avena fatua and Bidens pilosa. Environ Exp Bot. 2002;47(2):149-55.

17. Batish D, Singh H, Saxena D, Kohli R. Weed suppressing ability of parthenin-a sesquiterpene lactone from Parthenium hysterophorus. N Z Plant Prot. 2002;55:218-21.

18. Patel VS, Chitra V, Prasanna PL, Krishnaraju V. Hypoglycemic effect of aqueous extract of Parthenium hysterophorus L. in normal and alloxan induced diabetic rats. Indian J Pharmacol. 2008;40(4):183.

19. Selmi S, Rtibi K, Grami D, Sebai H, Marzouki L. Rosemary (Rosmarinus officinalis) essential oil components exhibit anti-hyperglycemic, anti-hyperlipidemic and antioxidant effects in experimental diabetes. Pathophysiology. 2017;24(4):297-303.

20. Asghari B, Zengin G, Bahadori MB, Abbas-Mohammadi M, Dinparast L. Amylase, glucosidase, tyrosinase, and cholinesterases inhibitory, antioxidant effects, and GC-MS analysis of wild mint (Mentha longifolia var. calliantha) essential oil: A natural remedy. Eur J Integr Med. 2018;22:44-9.

21. Borah A, Paw M, Gogoi R, Loying R, Sarma N, Munda S, et al. Chemical composition, antioxidant, anti-inflammatory, anti-microbial and in-vitro cytotoxic

efficacy of essential oil of Curcuma caesia Roxb. leaves: An endangered medicinal plant of North East India. Ind Crops Prod. 2019;129:448-54.

22. Manssouri M, Znini M, Majidi L. Studies on the antioxidant activity of essential oil and various extracts of Ammodaucus leucotrichus Coss. & Dur. Fruits from Morocco. J Taibah Uni Sci. 2020;14(1):124-30.

23. Zhang C, Zhao J, Famous E, Pan S, Peng X, Tian J. Antioxidant, hepatoprotective and antifungal activities of black pepper (Piper nigrum L.) essential oil. Food Chem. 2021;346:128845.

24. Dwivedi S, Dwivedi A, Dwivedi S. Folk lore uses of some plants by the tribes of Madhya Pradesh with special reference to their conservation. Ethnobot leafl. 2008;2008(1):105.

25. Shabbir A, Javaid A. Effect of aqueous extracts of alien weed Parthenium hysterophorus and two native asteraceous species on germination and growth of mungbean, Vigna radiata L. wilczek. J Agric Res. 2010;48(4):483-8.

26. Towers G, Subba Rao P, editors. Impact of the pan-tropical weed, Parthenium hysterophorus L. on human affairs. Proceedings of the first international weed control congress; 1992: Weed Science Society of Victoria Melbourne.

27. Espinosa-Rivero J, Rendón-Huerta E, Romero I. Inhibition of Helicobacter pylori growth and its colonization factors by Parthenium hysterophorus extracts. J Ethnopharmacol. 2015;174:253-60.

28. Tanvir R, Sajid I, Hasnain S. Screening of endophytic Streptomycetes isolated from Parthenium hysterophorus L. against nosocomial pathogens. Pak J Pharm Sci. 2013;26(2):277-83.

29. Deka B, Jiten M, Bhattacharyya P. Acaricidal activities of Parthenium hysterophorus L. against red spider mite, Oligonychus coffeae Nietner (Acarina: Tetranychidae) of tea. Int J Sci Res. 2013;4(5):2319-7064.

30. Krishnaveni M. Air pollution tolerance index and antioxidant activity of Parthenium hysterophorus. J Pharm Res. 2013;7(4):296-8.

31. Nabi SA, Kasetti RB, Sirasanagandla S, Tilak TK, Kumar MVJ, Rao CA. Antidiabetic and antihyperlipidemic activity of Piper longum root aqueous extract in STZ induced diabetic rats. BMC Complement Altern Med. 2013;13(1):37.

32. Panwar R, Sharma AK, Dutt D, Pruthi V. Phenolic acids from Parthenium hysterophorus: evaluation of bioconversion potential as free radical scavengers and anticancer agents. Advances in Bioscience and Biotechnology. 2015;6(01):11.

33. Anup R, Sainath P, Padma S, Sukumaran M. In vitro cytotoxic activity of methanolic extract of Parthenium hysterophorus flowers on MCF-7 and HeLa cell lines. Int J Curr Microbiol Appl Sci. 2015;4(8):710-5.

34. Kumar S, Nair G, Singh AP, Batra S, Wahab N, Warikoo R. Evaluation of the larvicidal efficiency of stem, roots and leaves of the weed, Parthenium hysterophorus (Family: Asteraceae) against Aedes aegypti L. Asian Pac J Trop Dis. 2012;2(5):395-400.

35. Kumar VP, Chauhan NS, Padh H, Rajani M. Search for antibacterial and antifungal agents from selected Indian medicinal plants. J Ethnopharmacol. 2006;107(2):182-8.

36. Jha U, Chhajed PJ, Oswal RJ, Shelke TT. Skeletal muscle relaxant activity of methanolic extract of Parthenium hysterophorus L. leaves in swiss albino mice. Int J Pharm Life Sci. 2011;2(11).

37. Datta S, Saxena DB. Pesticidal properties of parthenin (from Parthenium hysterophorus) and related compounds. Pest Manag Sci. 2001;57(1):95-101.

38. Bharadwaja S, Singh S, Moholkar VS. Design and optimization of a sono-hybrid process for bioethanol production from Parthenium hysterophorus. J Taiwan Inst Chem Eng. 2015;51:71-8.

39. Parashar V, Parashar R, Sharma B, Pandey AC. Parthenium leaf extract mediated synthesis of silver nanoparticles: a novel approach towards weed utilization. Dig J Nanomater Biostructures. 2009;4(1).

40. Rajiv P, Rajeshwari S, Venckatesh R. Bio-Fabrication of zinc oxide nanoparticles using leaf extract of Parthenium hysterophorus L. and its size-dependent antifungal activity against plant fungal pathogens. Spectrochimica Acta Part A: Mol Biomol Spectrosc. 2013;112:384-7.

41. Yang J, Jiang F, Ma C, Rui Y, Rui M, Adeel M, et al. Alteration of crop yield and quality of wheat upon exposure to silver nanoparticles in a life cycle study. J Agric Food Chem. 2018;66(11):2589-97.

42. Ahmad MA, Yuesuo Y, Ao Q, Adeel M, Hui ZY, Javed R. Appraisal of comparative therapeutic potential of undoped and nitrogen-doped titanium dioxide nanoparticles. Molecules. 2019;24(21):3916.

43. Javed R, Ahmad MA, Gul A, Ahsan T, Cheema M. Comparison of chemically and biologically synthesized nanoparticles for the production of secondary metabolites, and growth and development of plants. Compr Anal Chem. 2021;94:303-29.

44. Ahmed M, Ji M, Qin P, Gu Z, Liu Y, Sikandar A, et al. Determination of phytochemicals, antioxidant activity and biochemical composition of chinese

mugwort (*Artemisia argyi* l.) leaf extract from northeast China. Appl Ecol Environ Res. 2019;17(6):15349-62.

45. Sparkman OD. Identification of essential oil components by gas chromatography/quadrupole mass spectroscopy Robert P. Adams. J Am Soc Mass Spectrom. 2005;16(11):1902.

46. McConnachie A, Strathie L, Mersie W, Gebrehiwot L, Zewdie K, Abdurehim A, et al. Current and potential geographical distribution of the invasive plant Parthenium hysterophorus (Asteraceae) in eastern and southern Africa. Weed Res. 2011;51(1):71-84.

47. Narasimhan T, Murthy BK, Harindranath N, Rao PS. Characterization of a toxin fromParthenium hysterophorus and its mode of excretion in animals. J Bios. 1984;6(5):729-38.

48. Hasan N, Jain R. Biotoxicity of Parthenium hysterophorus extracts against Meloidogyne javanica and Helicotylenchus dihystera. Nematol Mediterr. 1984.

49. Kalaiselvi M, Subbaiya R, Selvam M. Synthesis and characterization of silver nanoparticles from leaf extract of Parthenium hysterophorus and its anti-bacterial and antioxidant activity. Int J Curr Microbiol Appl Sci. 2013;2(6):220-7.

50. Arshad M, Mumtaz MW, Chaudhary AR, Rashid U, Ali M, Mukhtar H, et al. Metabolite profiling of Cycas revoluta leaf extract and docking studies on alpha-glucosidase inhibitory molecular targets by phytochemicals. PakJ Pharm Sci. 2019;32(2 (Supplementary)):871-4.

51. Sinha SN, Paul D. Antioxidant potentials of parthenium hysterophorus L. leaf extracts. Sci Res J India. 2014;3(2):80-6.

52. Bashir S, Raza, S.A., Ahmad, M., Farhan, M., Ali, S. and William, J. Hydroethanolic leaf extract of Parthenium hysterophorus attenuates blood glucose in alloxan induced diabetic mice. Food Res 2021;5(1):72-7.

53. Ahmad J, Bagheri R, Bashir H, Baig MA, Al-Huqail A, Ibrahim MM, et al. Organ-specific phytochemical profiling and antioxidant analysis of Parthenium hysterophorus L. BioMed Res Int. 2018;2018.

54. Adeel M, Zain M, Fahad S, Rizwan M, Ameen A, Yi H, et al. Natural and synthetic estrogens in leafy vegetable and their risk associated to human health. Environ Sci Pollut Res. 2018;25(36):36712-23.

55. Rajiv P, Rajeshwari S, Yadav RH, Rajendran V. Vermiremediation: Detoxification of Parthenin toxin from Parthenium weeds. J Hazard Mater. 2013;262:489-95.

56. Tesfu F, Emana G. Evaluation of Parthenium hysterophorus L. powder against Callosobruchus chinensis L.(Coleoptera: Bruchidae) on chickpea under laboratory conditions. Afri J Agri Res. 2013;8(44):5405-10.

57. Shaheen FA, Khaliq A. Management of Pulse Beetle, Callosobruchus chinensis L.(Coleoptera: Bruchidae) in stored Chickpea using ashes, red soil powder and turpentine oil. Pak Entomol. 2005;27(2):19-24.

58. Yadav SK, Patel S. Insecticidal and repellent activity of some plant extracts against Myzus persicae (Sulzer) and Brevicoryne brassicae(Linnaeus). J Entomol Zool Stud. 2017;5(2):1434-143.

59. Wasu Yogesh H GD, Patel Sagar B. Larvicidal effects of *Parthenium hysterophorus* against polyphagus pests *Spodoptera Litura* and *Spodoptera littoralis*. Indian J Appl Res. 2019;9(2):1-19.

60. Wang M-H, Gan J-J, Chen W-H, Guan Y-L. Chemical Constituents of Parthenium hysterophorus. Chem Nat Comp. 2020;56:556-8.

61. Białoń M, Krzyśko-Łupicka T, Nowakowska-Bogdan E, Wieczorek PP. Chemical composition of two different lavender essential oils and their effect on facial skin microbiota. Molecules. 2019;24(18):3270.

62. Hudz N, Makowicz E, Shanaida M, Białoń M, Jasicka-Misiak I, Yezerska O, et al. Phytochemical Evaluation of Tinctures and Essential Oil Obtained from Satureja montana Herb. Molecules. 2020;25(20):4763.

63. Korzeniowska K, Łęska B, Wieczorek PP. Isolation and determination of phenolic compounds from freshwater Cladophora glomerata. Algal Res. 2020;48:101912.

64. Bajwa AA, Weston PA, Gurusinghe S, Latif S, Adkins SW, Weston LA. Toxic Potential and Metabolic Profiling of Two Australian Biotypes of the Invasive Plant Parthenium Weed (Parthenium hysterophorus L.). Toxins. 2020;12(7):447.

65. Koo S, Thomas HR, Daniels SD, Lynch RC, Fortier SM, Shea MM, et al. A breath fungal secondary metabolite signature to diagnose invasive aspergillosis. Clin Infect Dis. 2014;59(12):1733-40.

66. Yang D, Michel L, Chaumont J-P, Millet-Clerc J. Use of caryophyllene oxide as an antifungal agent in an in vitro experimental model of onychomycosis. Mycopathologia. 2000;148(2):79-82.

67. Saifullah K. Callus induction and cell suspension culture production of Catharanthus roseus for biotransformation studies of (-)-caryophyllene oxide. Pak J Bot. 2011;43(1):467-73.

68. Halim SA, Uddin R, Madura JD. Benchmarking docking and scoring protocol for the identification of potential acetylcholinesterase inhibitors. J Mol Graph Model. 2010;28(8):870-82.

69. Mulyaningsih S, Sporer F, Reichling J, Wink M. Antibacterial activity of essential oils from Eucalyptus and of selected components against multidrug-resistant bacterial pathogens. Pharm Biol. 2011;49(9):893-9.

Tables

Table 1. Physical appearance of the flower's extract of *Parthenium hysterophorus*.

Extract	Visibility	Physical Nature	Consistency
EtOH	Bluish green	Oily	Consistent
EA	Greenish	Oily	Consistent
DCM	Brownish	Oily/Gummy	Non-consistent
DW	Brownish	Oily/Gummy	Non-consistent

EtOH; (Ethanol), EA; (Ethyl Acetate), DCM; (Dichloromethane), DW; (Distilled water).

Table 2. Total Phenol, Flavonoid content and DPPH radical scavenging activity of the flower's extract of *Parthenium hysterophorus*.

Extracts	Phenol (GAE/g)	Flavonoid (QE/g)	DPPH Inhibition (%)
EtOH	27.55±1.46^{c}	14.23±0.10^{b}	88.53±0.11^{b}
EA	47.79±0.85^{b}	138.67±2.20^{a}	67.21±0.19^{c}
DCM	30.50±0.00^{d}	15.38±0.04^{c}	95.03±1.58^{a}
DW	103.30±1.16^{a}	4.81±0.01^{c}	18.73±0.89^{d}
Statics			
	S.S= 24268	S.S= 248063	S.S= 13880
	M.S=8069	M.S= 82687	M.S= 4626
	Df= 3	Df= 3	M.S= 3
	f= 2564***	*f*= 22488***	*f*= 1031***

Data in the columns is described as mean values ± standard deviations with various superscripts are significantly different according to DMRT" *P*> 0.05). S.S (Sum of square); M.S (Mean square); Df (Degree of freedom); *f* (Significance); *** (significance level).

Table 3. Correlation of the antioxidant activity of *P. hysterophorus* extract versus Phenols, Flavonoids and extract yield.

Treatments	Phenols	Flavonoids	DPPH	Yield
Phenols	1	-0.16	-0.99**	-0.11
Flavonoids	-0.16	1	-0.71	-0.90
DPPH	-0.99**	-0.71	1	-0.10
Yield	-0.11	-0.09	-0.10	1

Note: * Correlation is significant at 0.05 levels; ** Correlation is highly significant at 0.05 levels

Table 4. **Toxicity of *P. hysterophorus* extracts against *B. brassicae* at 12, 24, 48, 72 and 96 hours exposure**

Time	LC_{50} µgmL^{-1}	95% F.L		Slope± S.E	χ2
		Lower	Upper		
12	3598	1421	35512	1.09±0.38	0.25
24	2569	1321	27758	1.51±0.46	0.99
48	2345	1153	25517	1.31±0.53	0.85
72	1446	852	21721	1.56±0.63	0.22
96	750	468	1276	1.89±0.58	0.04

Note: LC_{50} (Lethal concentration); S.E. (standard error); χ2 (chi-square); F.L. (Fiducial limit)

Table 5. Chemical composition of the flower's extract of *Parthenium hysterophorus*.

Peak #	R. T	Area %	Chemical Compounds	M.F	M.W g/mol
1	3.22	7.05	Benzene, 1,3-dimethyl-	C_8H_{10}	106.17
2	3.92	3.47	2,4-Dimethylamphetamine	$C_{11}H_{17}N$	163.26
3	4.43	2.04	3-Piperidinol	$C_5H_{11}NO$	101.15
4	5.05	2.74	2-Pentene, 4,4-dimethyl-, (E)-	C_7H_{14}	98.186
5	5.52	3.55	2-Butanamine	$C\ _6H\ _5N$	101.19
6	12.96	25.93	2-Naphthalenemethanol,decahydro.alpha.,.α.,4a-trimethyl-8-methylene-,[2R (2 α.,4a.α.,8a.beta.)]-	$C_{15}H_{26}O$	222.36
7	14.29	11.27	3-Methoxy-3,4-dimethyl-1-heptyne	$C_{10}H_{18}O$	154.25
8	15.21	4.76	Undecane	$C_{11}H_{24}$	156.31
9	15.85	3.82	2-Pyrrolidinone, 1-methyl-	C_5H_9NO	99.13
10	16.62	7.26	Glycinamide hydrochloride	$C_2H_7ClN_2O$	110.54
11	17.58	2.18	1,2-Benzenedicarboxylic acid, butyl 2-ethylhexyl ester	$C_{20}H_{30}O_4$	334.45
12	18.1	6.67	Hexadecanoic acid, ethyl ester	$C_{18}H_{36}O_2$	284.48
13	21.85	19.25	Histidine, 1,N-dimethyl-4-nitro-	$C_8H_{10}N_2O_2$	166.18

R.T (Retention time); M.F (Molecular formula); M.W (Molecular Weight).

Reviewer Comments:

Reviewer 1

Review comments:

Page 9

Point 1: innate of what? do you mean native?

Response:

The word innate changed to inherent

Point 2.3; need to explain how?

Response:

Sentence was explained in detail to clarify the meaning

Point 4,5 No clear, check the grammar

Response: compliance has been done and sentence was revised

Point 6, 7 Again, this need to be explained how?

Response compliance has been done and explained to clear the meaning

Point 8, 9 Is this correct referencing style?

Response: reference style has been adjusted according the journal

Point, 10 not clear

Response sentence was revised to clarify the meaning

Page 10

Point 1,2,3 Do you mean parthenin?

Response: yes, compliance was done

Point 4,5,6 move 'health' before 'consequences'

Response: compliance has been done and sentence was revised as suggested

Point 7,8 Do you mean "come in contact'

Response: yes the same meaning

Point 8, add author citations with the sci. names of all plants when mentioned first time

Response: compliance has been made and instruction were followed

Page 16

Point 1 highest?

Response: compliance was done

Page 21

Point 1 leaves.?

Response: sentence was revised.

Point 2 Either use Parthenium or Parthenium hysterophorus, throughout

Response: compliance has been done and instruction was followed.

Page 36

Point C.L stands for?

Response: actually the word is F.L stands for Fiducial Limit

Page 39

Point 1G = +ve control missing in Figure

Response: Positive control was added in the figure

Reviewer 1

Point 1 Also add authority

Response: compliance was made and Authority was added

Point 2 Do not use such useless abbreviations. Write full words for these names or parameters.

Response: Instructions were followed and useless abbreviation were deleted

Point 3 Give space

Response: compliance was made

Point 4 showed.?

Response: compliance was made and the word changed to “showed”

Point: 5 No need of First paragraph.

Response: First paragraph deleted.

Point 6 CIte reference correctly. Delete the name of authors

Response: reference was cited in correct style

Point 7 This paragraph should be extended

Response: The suggested paragraph was extended.

Point 8 It is ......

Response Compliance has been made

Point 9 Add a references here.; Following latest references is suggested:

Response The suggested reference were added and cited in the manuscript

Point 10 Should not be a new paragraph

Response Compliance was made and Instructions were followed

Point: 11 No need to mention the Institute.

Response: Information about institute were deleted

Point: 12 Do not use capital letters

Response: suggestion were followed, and word were corrected

Point: 13 Delete %

Response: The symbol % was removed

Point: 14 Add space

Response: The space was added where needed

Point 15 Do not use upper case letters for the names of compounds

***Response:*:** Compliance has been done

Point 16 No need of this introductory paragraph

Response: A part of the paragraph has been deleted

Point 17 Format references uniformly and correctly

Response: References were formatted according to the journal style

Rebuttal Letter

Title:
[Antioxidant, Insecticidal Activity and Chemical Profiling of Flower's Extract of Parthenium weed (*Parthenium hysterophorus* L.]

[PlosOne]

[Submission Date: May 26, 2022]

Dear Editor

Thank you for inviting us to submit a revised draft of our manuscript entitled, "Antioxidant, Insecticidal Activity and Chemical Profiling of Flower's Extract of Parthenium weed (*Parthenium hysterophorus* L. PlosOne journal. We also appreciate the time and effort you and each of the reviewers have dedicated to providing insightful feedback on ways to strengthen our paper. Thus, it is with great pleasure that we resubmit our article for further consideration.

We have incorporated changes that reflect the detailed suggestions you have graciously provided. We also hope that our edits and the responses we provide below satisfactorily address all the issues and concern you and the reviewers have noted.
To facilitate your review of our revisions, the following is a point-by-point response to the questions and comments.

EDITOR COMMENTS [1st round]

The present manuscript is informative and interpretation of obtained results is satisfactory. But the presentation of the manuscript is poor and must be improved significantly as suggested by the reviewers. Moreover, detail method used for the Extraction and Purification of Biochemical Compound should be described in little detail or relavant reference should be provided.
Negative and positive control must be considered rationally in each assay as one of the major concerns of the reviewer.
Authors must be careful in presenting the units [unit of LC50 of residual assay in table 3 is µg/mL while in text (result section 2.3 Insecticidal Activity) mentioned mg/mL].

Response: **Compliance has been done**;

- Manuscript has edited by native English speaker/TOPEDIT SCIENTIFIC EDITING [Http://www.topeditsci.com]
- Negative and Positive control has been added where needed as suggested by the reviewers.
- In Table 3 is µg/mL has been corrected as point out by the reviewer.

Reviewer Comments:

Reviewer 1

1. Introduction:
1- Introduction part should be started by specific paragraph regarding the microbial resistance, infectious diseases, reactive species, oxidative stress, and the role of naturally occurring compounds in the treatment of such issues.

Instructions have been followed and a paragraph has been added at the start of the introduction as suggested by the reviewer along with references;

"Oxidative stress plays a double role in infections; the pathologies that arise during such infections can be attributed to oxidative trauma and the creation of reactive species, often with lethal consequences. Microbial resistance to conventional antibiotics poses a significant threat to the treatment of infectious diseases. However, phytochemicals exhibit latent biological activity towards both resistant and sensitive pathogens. Phytochemicals are a valuable source of bioactive compounds with antimicrobial activities. Among these phytochemicals, phenolics are diverse secondary metabolites such as tannins, flavonoids, and lignin that exhibit antioxidant properties and are abundant in plant tissues. Likewise, reactive oxygen species (ROS) are produced as typical products in plant cellular breakdown. Naturally occurring compounds play a vital role against microbial resistance in the management of infectious diseases."

2- The sentence "Natural plants are venerated source of…." should be typed as "Medicinal plants are venerated source of….".

Response: 2. Compliance has been done and stated as ***Medicinal plants*** are venerated source of phytochemical compounds………..

3. Discussions:

1- The sentence "Natural plants are God gifted treasures….." should be typed as "Medicinal plants are God gifted treasures…..".

Response: 1. Instructions have been followed and statement has been revised as ***Medicinal plants*** are God gifted treasures for humans which possess …..

2- Please, re-write this section "Moreover, all of the populations of C. colocynthis extract showed antibacterial activity against Pseudomonas aeruginosa and Escherichia coli, Enterococcus faecalis and Staphylococcus aureus and antifungal activity against four Candidia species i.e. Candida krusei, Candida glabrata, Candida parapsilosis and Candida albicans [32]", try to delete the repeated word (and).

Response: 2. Instructions have been followed and statement has been revised as suggested

Moreover, all of the populations of *C. colocynthis* extract showed antibacterial activity against *Pseudomonas aeruginosa*, *Escherichia coli*, *Enterococcus faecalis* and *Staphylococcus aureus* whereas, antifungal activity against four Candidia species i.e. *Candida krusei*, *Candida glabrata*, *Candida parapsilosis* and *Candida albicans* [32]

3- In the section "Similarly, a flavonoid4'-methoxy-5,7-dihydroxyflavone6-C-glucoside isolated.", the term (-C-) should be typed in italic font.

Response: 3. Instructions have been followed and the term has been revised as

Similarly, a flavonoid 4'-methoxy-5,7-dihydroxyflavone 6-*C*-glucoside isolated

Materials and Methods:

1- If allowed, a molecular docking study should be performed.

Response: 1. As labs are closed, shortage of time and mainly, COVID-19 Pandemic and Omicron etc. the docking study at this stage may not be possible; however in future this should be followed in another experiment

Abbreviations:

1- A list of abbreviations should be inserted by the end of the manuscript before references.

Response: 1. Compliance has been done as suggested by the reviewer

1. DPPH (1, 1-diphenyl-2-picrylhydrazyl)
2. EC_{50} (Half maximal effective concentration)
3. LC_{50}; (Lethal concentration)
4. ABTS (2, 2'-Azino-Bis-3-Ethylbenzothiazoline-6-Sulfonic Acid)
5. TEV (Tobacco Etch Virus)
6. CMV (Cucumber Mosaic Virus)
7. Conc. (Concentration)
8. PDA (Potatoes Dextrose Agar)
9. WP (Wettable Powder)

References:

1. All Scientific names of plants should be typed in italic fonts.

Response: 1. All the scientific names of the pants has been changed into italic fonts in the ref list

2. All Scientific names of microorganisms should be typed in italic fonts.

Response: 2. All the scientific names of microorganisms has been changed into italic fonts

3. In some references; page number, volume number, and issue number were missed.

Response: 3. Compliance has been done; page number, volume number, and issue number has been added as suggested.

4. Ref. No. 10: The word (linn.) should be typed as (Linn.), also the plant name should be typed as (Luffa acutangula).

Response: 4. Instructions have been followed and the word (linn.) typed as (Linn.), as well as re-typed as *Luffa acutangula*.

Ref No 10; Soam, P. S., Singh, T. & Vijayvergia, R. Short Communication *Citrullus colocynthis* (Linn.) and *Luffa acutangula* (l.) Roxb, schrad. Source of bioinsecticides and their contribution in managing climate change. in (2013).

5. Ref. No. 10: The word (And) should be typed as (and).

Response: 5. Instructions have been followed, And written as “and”

Ref No 10; Soam, P. S., Singh, T. & Vijayvergia, R. Short Communication *Citrullus colocynthis* (Linn.) *and Luffa acutangula* (l.) Roxb, schrad. Source of bioinsecticides and their contribution in managing climate change. in (2013).

6. In some references; the complete list of authors were missed except first author (e.g., Ref. No. 12; Ahmed, M. et al.).

Response: 6. Instructions have been followed and a complete list of authors has been added in the references list.

Supplementary Files S1:

1. Add the word "and" before the last 1H-NMR and 13C-NMR value and the chemical shift unit (ppm).

Response: 6. Compliance has been done as suggested like……………..
1D (^{1}H-NMR and ^{13}C-NMR),

REVIEWER 2

1. There are numerous typos and grammatical mistakes that I’m not able to point them one by one (for example, using Upper-Lower case for the name of compounds, disease and pathogens; repeated full name of pathogen; not using comma before "respectively", and so on)

Response: 1. Compliance has been done and the Manuscript also has been revised and edited by a English speaker/ TOPEDIT SCIENTIFIC EDITING [Http://www.topeditsci.com]

2. Spinasterol and 22,23-dihydrospinasterol presented as mixture. But the author does not give any evidence for a 5:4 ratio of this mixture. The MS and NMR spectra were not clear to determine the ratio of this mixture compounds.

Response: 2. The mass spectrometer examination showed molecular ion peak (M) at *m/z* 414 and 412 which suggested the molecular formula as a mixture of $C_{29}H_{50}O$, $C_{29}H_{48}O$ with the ratio of 5:4 calculated by the available results of mass spectrum; elemental analysis and nuclear magnetic resonance analysis (^{1}H-NMR and ^{13}C-NMR) suggested the ratio of 5:4.

3. These authors did not show the positive and negative control to indicate the activities of these compounds (Table 1, 3, 4, 5). So the data is not enough to conclude the biological activities of this mixture.

Response: 3. Compliance has been done and positive as well as negative control has been added in the table where needed as;

Table 1. For DPPH assay methanol was used as a negative control and the DPPH solution only (without the sample). Whereas, DPPH+ methanol was used as experimental control. Therefore, negative control was usually methanol and DPPH. Negative control was prepared to obtain the absorbance of the DPPH before reacting with the sample.
However, the positive control was prepared in order to compare the results of the samples with those obtained from a standard compound.
Table 4. Positive control was added in the Table 3 which was missed by mistake,
Table 3. As LC_{50} was calculated from the raw data obtained during calculation of mortality, So, it was not compulsory to calculate or mention the mortality of positive control. However, positive control was mentioned now in the Table 4.
Table 5. In the Table 5 Correlation of antioxidant activity versus antifungal and insecticidal activity was presented, as data was used from the already prepared tables, so, only correlation was calculated.

2nd round

ADDITIONAL EDITOR SUGGESTIONS: [2nd round]

1. [Manuscript is acceptable as the authors addressed the reviewer's comments. I suggest the author replace the common name 'carrot grass' with 'parthenium weed' in title and throughout the manuscript at proof stage.]

RESPONSE: [The common name 'carrot grass' has been replaced with 'parthenium weed' in title and throughout the manuscript]

REVIEWER 1 COMMENTS:

1[Innate of what? do you mean native?]
RESPONSE: [The word innate changed to inherent]
2[Point 2.3; need to explain how?]
RESPONSE: [Sentence was explained in detail to clarify the meaning]
3[Point 4,5 No clear, check the grammar]

RESPONSE: [compliance has been done and sentence was revised]
4[Point 6, 7 Again, this need to be explained how?]
RESPONSE: [compliance has been done and explained to clear the meaning]
5[Point 8, 9 Is this correct referencing style?]
RESPONSE: [reference style has been adjusted according the journal]
6 [Point, 10 not clear]
RESPONSE: [sentence was revised to clarify the meaning]
7[Point 1,2,3 Do you mean parthenin?]
RESPONSE [yes, compliance was done]
8[Point 4,5,6 move 'health' before 'consequences'?]
RESPONSE: [compliance has been done and sentence was revised as suggested]
9[Point 7,8 Do you mean "come in contact']
RESPONSE: [***:*** yes the same meaning]
10 [Point 8, add author citations with the sci. names of all plants when mentioned first time
RESPONSE: [compliance has been made and instruction were followed]
11 [Point 1 highest?
RESPONSE: [compliance was done]
12 [Point 1 leaves.?]
RESPONSE: [***:*** sentence was revised.]
13 [Point 2 Either use Parthenium or Parthenium hysterophorus, throughout
RESPONSE: compliance has been done and instruction was followed.
14 [Point C.L stands for?]
RESPONSE: actually the word is F.L stands for Fiducial Limit
15 [Point 1G = +ve control missing in Figure]
RESPONSE: Positive control was added in the figure

Reviewer 2

1 [Point 1 Also add authority]
RESPONSE: compliance was made and Authority was added
2 [*Point 2* Do not use such useless abbreviations. Write full words for these names or parameters.]
RESPONSE: Instructions were followed and useless abbreviation were deleted
3 [Point 3 Give space]
RESPONSE: compliance was made
4 [Point 4 showed.?]
RESPONSE: compliance was made and the word changed to "showed"
5 [Point: 5 No need of First paragraph.]
RESPONSE: First paragraph deleted.
6 [***Point 6*** CIte reference correctly. Delete the name of authors]
RESPONSE: reference was cited in correct style
7 [Point 7 This paragraph should be extended]
RESPONSE: The suggested paragraph was extended.
8 [Point 8 It is ......]
RESPONSE Compliance has been made
9 [Point 9 Add a references here.; Following latest references is suggested:]
RESPONSE The suggested reference were added and cited in the manuscript
10 [Point 10 Should not be a new paragraph]
RESPONSE Compliance was made and Instructions were followed
11 [Point: 11 No need to mention the Institute.]

RESPONSE*:* Information about institute were deleted
12 [Point: 12 Do not use capital letters]
RESPONSE*:* suggestion were followed, and word were corrected
13 [Point: 13 Delete %]
RESPONSE*:* The symbol % was removed
14 [Point: 14 Add space]
RESPONSE*:* The space was added where needed
15 [Point 15 Do not use upper case letters for the names of compounds]
RESPONSE: Compliance has been done
16 [Point 16 No need of this introductory paragraph]
RESPONSE*:* A part of the paragraph has been deleted
17 [Point 17 Format references uniformly and correctly]
RESPONSE*:* References were formatted according to the journal style

CONCLUDING REMARKS: Again, thank you for giving us the opportunity to strengthen our manuscript with your valuable comments and queries. We have worked hard to incorporate your feedback and hope that these revisions persuade you to accept our submission.

Sincerely,

Zumin Gu**.**

Corresponding Author
Associate Professor, College of Plant Protection
Shenyang Agricultural University, Shenyang
No.120 Dongling Road Shenhe District Liaoning, CHINA

guzumin1212@163.com
Tel: +86-24- 8848- 7148; Fax: +86-24-8834-2
