# Supplementary material for: Antioxidant, insecticidal activity and chemical profiling of flower’s extract of Parthenium weed (Parthenium hysterophorus L.)
Source: PLoS One. 2024 Jun 7;19(6):e0296321. doi: 10.1371/journal.pone.0296321 (PMC11161021; doi:10.1371/journal.pone.0296321)
Supplement: Attachment — Submitted filename: Rebuttal Letter.docx [file pone.0296321.s004.docx]

# Rebuttal Letter

**Title:**

[Antioxidant, Insecticidal Activity and Chemical Profiling of Flower’s Extract of Parthenium weed (*Parthenium hysterophorus* L.]

[PlosOne]

[Submission Date: May 26, 2022]

Dear Editor

Thank you for inviting us to submit a revised draft of our manuscript entitled, "Antioxidant, Insecticidal Activity and Chemical Profiling of Flower’s Extract of Parthenium weed (*Parthenium hysterophorus* L. PlosOne journal. We also appreciate the time and effort you and each of the reviewers have dedicated to providing insightful feedback on ways to strengthen our paper. Thus, it is with great pleasure that we resubmit our article for further consideration.

We have incorporated changes that reflect the detailed suggestions you have graciously provided. We also hope that our edits and the responses we provide below satisfactorily address all the issues and concern you and the reviewers have noted.

To facilitate your review of our revisions, the following is a point-by-point response to the questions and comments.

**EDITOR COMMENTS [1^st^ round]**

The present manuscript is informative and interpretation of obtained results is satisfactory. But the presentation of the manuscript is poor and must be improved significantly as suggested by the reviewers. Moreover, detail method used for the Extraction and Purification of Biochemical Compound should be described in little detail or relavant reference should be provided.

Negative and positive control must be considered rationally in each assay as one of the major concerns of the reviewer.

Authors must be careful in presenting the units [unit of LC50 of residual assay in table 3 is µg/mL while in text (result section 2.3 Insecticidal Activity) mentioned mg/mL].

***Response:* Compliance has been done**;

- Manuscript has edited by native English speaker/TOPEDIT SCIENTIFIC EDITING [Http://www.topeditsci.com]
- Negative and Positive control has been added where needed as suggested by the reviewers.
- In Table 3 is µg/mL has been corrected as point out by the reviewer.

**Reviewer Comments:**

**Reviewer 1**

*1. Introduction:*

1- Introduction part should be started by specific paragraph regarding the microbial resistance, infectious diseases, reactive species, oxidative stress, and the role of naturally occurring compounds in the treatment of such issues.

Instructions have been followed and a paragraph has been added at the start of the introduction as suggested by the reviewer along with references;

“Oxidative stress plays a double role in infections; the pathologies that arise during such infections can be attributed to oxidative trauma and the creation of reactive species, often with lethal consequences. Microbial resistance to conventional antibiotics poses a significant threat to the treatment of infectious diseases. However, phytochemicals exhibit latent biological activity towards both resistant and sensitive pathogens. Phytochemicals are a valuable source of bioactive compounds with antimicrobial activities. Among these phytochemicals, phenolics are diverse secondary metabolites such as tannins, flavonoids, and lignin that exhibit antioxidant properties and are abundant in plant tissues. Likewise, reactive oxygen species (ROS) are produced as typical products in plant cellular breakdown. Naturally occurring compounds play a vital role against microbial resistance in the management of infectious diseases.”

2- The sentence ''Natural plants are venerated source of….'' should be typed as ''Medicinal plants are venerated source of….''.

***Response: 2.*** Compliance has been done and stated as ***Medicinal plants*** are venerated source of phytochemical compounds….….….

**3. Discussions:**

1- The sentence ''Natural plants are God gifted treasures…..'' should be typed as ''Medicinal plants are God gifted treasures…..''.

***Response: 1***. Instructions have been followed and statement has been revised as ***Medicinal plants*** are God gifted treasures for humans which possess …..

2- Please, re-write this section ''Moreover, all of the populations of C. colocynthis extract showed antibacterial activity against Pseudomonas aeruginosa and Escherichia coli, Enterococcus faecalis and Staphylococcus aureus and antifungal activity against four Candidia species i.e. Candida krusei, Candida glabrata, Candida parapsilosis and Candida albicans ^32^'', try to delete the repeated word (and).

***Response: 2***. Instructions have been followed and statement has been revised as suggested

Moreover, all of the populations of *C. colocynthis* extract showed antibacterial activity against *Pseudomonas aeruginosa, Escherichia coli*, *Enterococcus faecalis* and *Staphylococcus aureus* whereas, antifungal activity against four Candidia species i.e. *Candida krusei*, *Candida glabrata*, *Candida parapsilosis* and *Candida albicans* ^32^

3- In the section ''Similarly, a flavonoid4’-methoxy-5,7-dihydroxyflavone6-C-glucoside isolated.'', the term (-C-) should be typed in italic font.

***Response: 3***. Instructions have been followed and the term has been revised as

Similarly, a flavonoid 4’-methoxy-5,7-dihydroxyflavone 6-*C*-glucoside isolated

**Materials and Methods:**

1- If allowed, a molecular docking study should be performed.

***Response: 1***. As labs are closed, shortage of time and mainly, COVID-19 Pandemic and Omicron etc. the docking study at this stage may not be possible; however in future this should be followed in another experiment

**Abbreviations:**

1- A list of abbreviations should be inserted by the end of the manuscript before references.

***Response: 1***. Compliance has been done as suggested by the reviewer

1. DPPH (1, 1-diphenyl-2-picrylhydrazyl)
2. EC_50_ (Half maximal effective concentration)
3. LC_50_; (Lethal concentration)
4. ABTS (2, 2'-Azino-Bis-3-Ethylbenzothiazoline-6-Sulfonic Acid)
5. TEV ([Tobacco Etch Virus](https://en.wikipedia.org/wiki/Tobacco_etch_virus) )
6. CMV ([Cucumber Mosaic Virus](https://en.wikipedia.org/wiki/Cucumber_mosaic_virus))
7. Conc. (Concentration)
8. PDA (Potatoes Dextrose Agar)
9. WP (Wettable Powder)

References:

1. All Scientific names of plants should be typed in italic fonts.

***Response: 1***. All the scientific names of the pants has been changed into italic fonts in the ref list

2. All Scientific names of microorganisms should be typed in italic fonts.

***Response: 2***. All the scientific names of microorganisms has been changed into italic fonts

3. In some references; page number, volume number, and issue number were missed.

***Response: 3***. Compliance has been done; page number, volume number, and issue number has been added as suggested.

4. Ref. No. 10: The word (linn.) should be typed as (Linn.), also the plant name should be typed as (Luffa acutangula).

***Response: 4***. Instructions have been followed and the word (linn.) typed as (Linn.), as well as re-typed as *Luffa acutangula*.

Ref No 10; Soam, P. S., Singh, T. & Vijayvergia, R. Short Communication *Citrullus colocynthis* (Linn.) and *Luffa acutangula* (l.) Roxb, schrad. Source of bioinsecticides and their contribution in managing climate change. in (2013).

5. Ref. No. 10: The word (And) should be typed as (and).

***Response: 5***. Instructions have been followed, And written as “and”

**Ref No 10;** Soam, P. S., Singh, T. & Vijayvergia, R. Short Communication *Citrullus colocynthis* (Linn.) *and* *Luffa acutangula* (l.) Roxb, schrad. Source of bioinsecticides and their contribution in managing climate change. in (2013).

6. In some references; the complete list of authors were missed except first author (e.g., Ref. No. 12; Ahmed, M. et al.).

***Response: 6***. Instructions have been followed and a complete list of authors has been added in the references list.

**Supplementary Files S1:**

1. Add the word ''and'' before the last 1H-NMR and 13C-NMR value and the chemical shift unit (ppm).

***Response: 6***. Compliance has been done as suggested like………………

1D (^1^H-NMR and ^13^C-NMR),

REVIEWER 2

1. There are numerous typos and grammatical mistakes that I’m not able to point them one by one (for example, using Upper-Lower case for the name of compounds, disease and pathogens; repeated full name of pathogen; not using comma before "respectively", and so on)

***Response: 1***. Compliance has been done and the Manuscript also has been revised and edited by a English speaker/ TOPEDIT SCIENTIFIC EDITING [Http://www.topeditsci.com]

2. Spinasterol and 22,23-dihydrospinasterol presented as mixture. But the author does not give any evidence for a 5:4 ratio of this mixture. The MS and NMR spectra were not clear to determine the ratio of this mixture compounds.

***Response: 2***. The mass spectrometer examination showed molecular ion peak (M) at *m*/*z* 414 and 412 which suggested the molecular formula as a mixture of C_29_H_50_O, C_29_H_48_O with the ratio of 5:4 calculated by the available results of mass spectrum; elemental analysis and nuclear magnetic resonance analysis (^1^H-NMR and ^13^C-NMR) suggested the ratio of 5:4.

3. These authors did not show the positive and negative control to indicate the activities of these compounds (Table 1, 3, 4, 5). So the data is not enough to conclude the biological activities of this mixture.

***Response: 3***. Compliance has been done and positive as well as negative control has been added in the table where needed as;

**Table 1.** For DPPH assay methanol was used as a negative control and the DPPH solution only (without the sample). Whereas, DPPH+ methanol was used as experimental control. Therefore, negative control was usually methanol and DPPH. Negative control was prepared to obtain the absorbance of the DPPH before reacting with the sample.

However, the positive control was prepared in order to compare the results of the samples with those obtained from a standard compound.

**Table 4.** Positive control was added in the Table 3 which was missed by mistake,

**Table 3.** As LC_50_ was calculated from the raw data obtained during calculation of mortality, So, it was not compulsory to calculate or mention the mortality of positive control. However, positive control was mentioned now in the Table 4.

**Table 5.** In the Table 5 Correlation of antioxidant activity versus antifungal and insecticidal activity was presented, as data was used from the already prepared tables, so, only correlation was calculated.

**2^nd^ round**

**ADDITIONAL EDITOR SUGGESTIONS: [2^nd^ round]**

1. [Manuscript is acceptable as the authors addressed the reviewer’s comments. I suggest the author replace the common name ‘carrot grass’ with ‘parthenium weed’ in title and throughout the manuscript at proof stage.]

**RESPONSE: [**The common name ‘carrot grass’ has been replaced with ‘parthenium weed’ in title and throughout the manuscript]

**REVIEWER 1 COMMENTS:**

1[Innate of what? do you mean native?]

**RESPONSE:** [The word innate changed to inherent]

2[Point 2.3; need to explain how?]

**RESPONSE:** [Sentence was explained in detail to clarify the meaning]

3[Point 4,5 No clear, check the grammar]

**RESPONSE:** [compliance has been done and sentence was revised]

4[Point 6, 7 Again, this need to be explained how?]

**RESPONSE:** [compliance has been done and explained to clear the meaning]

5[Point 8, 9 Is this correct referencing style?]

**RESPONSE:** [reference style has been adjusted according the journal]

6 [Point, 10 not clear]

**RESPONSE: [**sentence was revised to clarify the meaning]

7[Point 1,2,3 Do you mean parthenin?]

**RESPONSE [**yes, compliance was done]

8[Point 4,5,6 move 'health' before 'consequences'?]

**RESPONSE:** [compliance has been done and sentence was revised as suggested]

9[Point 7,8 Do you mean "come in contact']

**RESPONSE:** [***:*** yes the same meaning]

10 [Point 8, add author citations with the sci. names of all plants when mentioned first time

**RESPONSE: [**compliance has been made and instruction were followed]

11 [Point 1 highest?

**RESPONSE:** [compliance was done]

12 [Point 1 leaves.?]

**RESPONSE:** [***:*** sentence was revised.]

13 [Point 2 Either use Parthenium or Parthenium hysterophorus, throughout

**RESPONSE:** compliance has been done and instruction was followed.

14 [Point C.L stands for?]

**RESPONSE:** actually the word is F.L stands for Fiducial Limit

15 [Point 1G = +ve control missing in Figure]

**RESPONSE:** Positive control was added in the figure

**Reviewer 2**

1 [Point 1 Also add authority]

**RESPONSE**: compliance was made and Authority was added

**2 [*Point 2*** Do not use such useless abbreviations. Write full words for these names or parameters.]

**RESPONSE**: Instructions were followed and useless abbreviation were deleted

3 [Point 3 Give space]

**RESPONSE**: compliance was made

***4*** [Point 4 showed.?]

**RESPONSE**: compliance was made and the word changed to “showed”

5 [Point: 5 No need of First paragraph.]

**RESPONSE**: First paragraph deleted.

***6*** [***Point 6*** CIte reference correctly. Delete the name of authors]

**RESPONSE**: reference was cited in correct style

7 [Point 7 This paragraph should be extended]

**RESPONSE**: The suggested paragraph was extended.

8 [Point 8 It is ......]

**RESPONSE** Compliance has been made

9 [Point 9 Add a references here.; Following latest references is suggested:]

**RESPONSE** The suggested reference were added and cited in the manuscript

10 [Point 10 Should not be a new paragraph]

**RESPONSE** Compliance was made and Instructions were followed

11 [Point: 11 No need to mention the Institute.]

**RESPONSE*:*** Information about institute were deleted

12 [Point: 12 Do not use capital letters]

**RESPONSE*:*** suggestion were followed, and word were corrected

13 [Point: 13 Delete %]

**RESPONSE*:*** The symbol % was removed

14 [Point: 14 Add space]

**RESPONSE*:*** The space was added where needed

15 [Point 15 Do not use upper case letters for the names of compounds]

**RESPONSE:** Compliance has been done

16 [Point 16 No need of this introductory paragraph]

**RESPONSE*:*** A part of the paragraph has been deleted

17 [Point 17 Format references uniformly and correctly]

**RESPONSE*:*** References were formatted according to the journal style

**CONCLUDING REMARKS**: Again, thank you for giving us the opportunity to strengthen our manuscript with your valuable comments and queries. We have worked hard to incorporate your feedback and hope that these revisions persuade you to accept our submission.

Sincerely,

Zumin Gu**.**

Corresponding Author

Associate Professor, College of Plant Protection

Shenyang Agricultural University, Shenyang

No.120 Dongling Road Shenhe District Liaoning, CHINA

[guzumin1212@163.com](mailto:guzumin1212@163.com)

*Tel: +86-24- 8848‐7148; Fax: +86-24-8834-2*
