# Supplementary material for: Antioxidant, insecticidal activity and chemical profiling of flower’s extract of Parthenium weed (Parthenium hysterophorus L.)
Source: PLoS One. 2024 Jun 7;19(6):e0296321. doi: 10.1371/journal.pone.0296321 (PMC11161021; doi:10.1371/journal.pone.0296321)
Supplement: Attachment — Submitted filename: Point by Point Reviewer Comments.docx [file pone.0296321.s003.docx]

Reviewer Comments:

**Reviewer 1**

**Review comments:**

**Page 9**

Point 1: innate of what? do you mean native?

***Response:***

The word innate changed to inherent

Point 2.3; need to explain how?

***Response:***

Sentence was explained in detail to clarify the meaning

Point 4,5 No clear, check the grammar

***Response:*** compliance has been done and sentence was revised

Point 6, 7 Again, this need to be explained how?

***Response*** compliance has been done and explained to clear the meaning

Point 8, 9 Is this correct referencing style?

***Response:*** reference style has been adjusted according the journal

Point, 10 not clear

***Response*** sentence was revised to clarify the meaning

**Page 10**

Point 1,2,3 Do you mean parthenin?

***Response:*** yes, compliance was done

Point 4,5,6 move 'health' before 'consequences'

***Response:*** compliance has been done and sentence was revised as suggested

Point 7,8 Do you mean "come in contact'

***Response:*** yes the same meaning

Point 8, add author citations with the sci. names of all plants when mentioned first time

***Response:*** compliance has been made and instruction were followed

**Page 16**

Point 1 highest?

***Response:*** compliance was done

**Page 21**

Point 1 leaves.?

***Response:*** sentence was revised.

Point 2 Either use Parthenium or Parthenium hysterophorus, throughout

***Response:*** compliance has been done and instruction was followed.

Page 36

Point C.L stands for?

***Response:*** actually the word is F.L stands for Fiducial Limit

**Page 39**

Point 1G = +ve control missing in Figure

***Response:*** Positive control was added in the figure

**Reviewer 1**

Point 1 Also add authority

***Response***: compliance was made and Authority was added

***Point 2*** Do not use such useless abbreviations. Write full words for these names or parameters.

***Response***: Instructions were followed and useless abbreviation were deleted

Point 3 Give space

***Response***: compliance was made

***Point 4*** ***showed.?***

***Response***: compliance was made and the word changed to “showed”

Point: 5 No need of First paragraph.

***Response***: First paragraph deleted.

***Point 6*** CIte reference correctly. Delete the name of authors

***Response***: reference was cited in correct style

Point 7 This paragraph should be extended

***Response***: The suggested paragraph was extended.

Point 8 It is ......

***Response*** Compliance has been made

Point 9 Add a references here.; Following latest references is suggested:

***Response*** The suggested reference were added and cited in the manuscript

Point 10 Should not be a new paragraph

***Response*** Compliance was made and Instructions were followed

Point: 11 No need to mention the Institute.

***Response:*** Information about institute were deleted

Point: 12 Do not use capital letters

***Response:*** suggestion were followed, and word were corrected

Point: 13 Delete %

***Response:*** The symbol % was removed

Point: 14 Add space

***Response:*** The space was added where needed

Point 15 Do not use upper case letters for the names of compounds

***Response:*:** Compliance has been done

Point 16 No need of this introductory paragraph

***Response:*** A part of the paragraph has been deleted

Point 17 Format references uniformly and correctly

***Response:*** References were formatted according to the journal style
